# The advantages of using drones over space-borne imagery in the mapping of mangrove forests

**DOI:** 10.1371/journal.pone.0200288

**Published:** 2018-07-18

**Authors:** Monika Ruwaimana, Behara Satyanarayana, Viviana Otero, Aidy M. Muslim, Muhammad Syafiq A., Sulong Ibrahim, Dries Raymaekers, Nico Koedam, Farid Dahdouh-Guebas

**Affiliations:** 1 Systems Ecology and Resource Management, Université libre de Bruxelles (ULB), Brussels, Belgium; 2 Ecology and Biodiversity (APNA), Vrije Universiteit Brussel (VUB), Brussels, Belgium; 3 Department of Biology, Universitas Atma Jaya Yogyakarta, Yogyakarta, Indonesia; 4 Mangrove Research Unit (MARU), Institute of Oceanography and Environment, Universiti Malaysia Terengganu (UMT), Kuala Terengganu, Malaysia; 5 Vlaamse Instelling voor Technologisch Onderzoek (VITO), Mol, Belgium; Kerala Forest Research Institute, INDIA

## Abstract

Satellite data and aerial photos have proved to be useful in efficient conservation and management of mangrove ecosystems. However, there have been only very few attempts to demonstrate the ability of drone images, and none so far to observe vegetation (species-level) mapping. The present study compares the utility of drone images (DJI-Phantom-2 with SJ4000 RGB and IR cameras, spatial resolution: 5cm) and satellite images (Pleiades-1B, spatial resolution: 50cm) for mangrove mapping—specifically in terms of image quality, efficiency and classification accuracy, at the Setiu Wetland in Malaysia. Both object- and pixel-based classification approaches were tested (QGIS *v*.2.12.3 with Orfeo Toolbox). The object-based classification (using a manual rule-set algorithm) of drone imagery with dominant land-cover features (i.e. water, land, *Avicennia alba*, *Nypa fruticans*, *Rhizophora apiculata* and *Casuarina equisetifolia*) provided the highest accuracy (overall accuracy (OA): 94.0±0.5% and specific producer accuracy (SPA): 97.0±9.3%) as compared to the Pleiades imagery (OA: 72.2±2.7% and SPA: 51.9±22.7%). In addition, the pixel-based classification (using a maximum likelihood algorithm) of drone imagery provided better accuracy (OA: 90.0±1.9% and SPA: 87.2±5.1%) compared to the Pleiades (OA: 82.8±3.5% and SPA: 80.4±14.3%). Nevertheless, the drone provided higher temporal resolution images, even on cloudy days, an exceptional benefit when working in a humid tropical climate. In terms of the user-costs, drone costs are much higher, but this becomes advantageous over satellite data for long-term monitoring of a small area. Due to the large data size of the drone imagery, its processing time was about ten times greater than that of the satellite image, and varied according to the various image processing techniques employed (in pixel-based classification, drone >50 hours, Pleiades <5 hours), constituting the main disadvantage of UAV remote sensing. However, the mangrove mapping based on the drone aerial photos provided unprecedented results for Setiu, and was proven to be a viable alternative to satellite-based monitoring/management of these ecosystems. The improvements of drone technology will help to make drone use even more competitive in the future.

## Introduction

With their luxuriant growth in tropical and subtropical latitudes along the land-sea interface, bays, estuaries, lagoons and backwaters [1], mangroves provide important ecosystem services, ranging from coastal protection to fisheries production, ecotourism, phytoremediation, carbon sequestration, and other services [2–4]. The value of mangroves were estimated to be between 4,185 and 50,349 USD per km^2^ [5]^,^ with a major part of these coming from indirect services [6]. Because of the less-valued direct services like firewood, timber and honey [6], mangrove ecosystems are often unappreciated or underestimated [7,8], resulting in conversion into settlements, agriculture, and aquaculture areas in many parts of the world [9–11]. In light of the decreasing mangrove cover and subsequent consequences [2,12–14], the world’s remaining mangroves need appropriate conservation and management, for which accurate and updated information is necessary [15].

Remote sensing is indispensable for mangrove research due to its time saving and cost-effective nature compensating for the fieldwork, which is difficult to carry out, especially in areas of low accessibility [11,15–18]. Regular monitoring/mapping of the mangroves based on remote sensing can provide authentic information (along with spatial-temporal dynamics) that is needed for better management [19,20]. In addition, past and present vegetation maps are useful to reconstruct past events and predict future development scenarios [21–23]. To date, both satellite images and airplane-based aerial photographs have been used for mangrove mapping [17,19,21,23–26]. Various types of satellite images–obtained from very low- to very high-resolution (VHR) sensors, have been used in relation to the scientific targets, including estimation of global mangrove cover [27,28] and regional/local species’ level distribution [29–31]. While some low- to moderate-resolution satellite data (*e*.*g*. Landsat, Sentinel) have still been available for free [28], the VHR images are expensive, and researchers using them are often constrained by their budget [11,32]. In addition, availability of cloud-free satellite images for mangrove mapping is a known difficulty [15,33].

Unlike satellite imagery, aerial photos are not challenged by cloud cover because their acquisition (aircraft flight) time can be adjusted to local weather conditions [34]. Their spatial resolution ranges from submeter to meter to centimeter level, depending on the flight altitude [15,34,35]. Aerial photos have probably been the only source of image documentation prior to satellite technology for identifying the past land-use/cover, and to date still produce imagery with the best spatial resolution for retrospective studies [36–38]. The significance and usefulness of archived and recently taken aerial photographs for genus to species level classification of the mangroves is evident from diverse publications [15,21,39].

The main limitation of an aircraft’s aerial photography for vegetation mapping is its high operational cost [15]. However, this situation is changing, with the revolutionary mode of data acquisition through Unmanned Aerial Vehicles (UAVs) or drones, which are not only reducing the cost of aerial photography, but also the cost of equipment, due to the production of updated models in the market [34]. Besides having higher spatial resolution [40,41], the sequential drone images can provide point-cloud 3D views to estimate tree height and crown diameter [42,43]. Although drone images have been used for monitoring and management of the terrestrial vegetation—from tall canopy trees to invasive weeds, agricultural crop yields, and other purposes [34,44–48]—the implications of their use for mangrove research are still limited, except for a few recent publications on leaf area index and the inventory of production forests [43,49]

In this study, we mapped the mangroves at Setiu Wetland, Malaysia, based on drone (DJI-Phantom-2) and satellite (Pleiades-1B) images, and compared the respective results. Being one of the first attempts in scientific literature to apply a drone for mangrove species’ mapping, our objective was not only to identify the potential of drone technology for mangrove research, but also to compare both drone and satellite imagery in terms of image quality (i.e. spatial, spectral, radiometric and temporal resolution), efficiency (i.e. coverage area, data acquisition/processing time and user-cost), and land-use/cover (i.e. object- and pixel-based) classification accuracy.

## Materials and methods

### Study area

**Fig 1:**
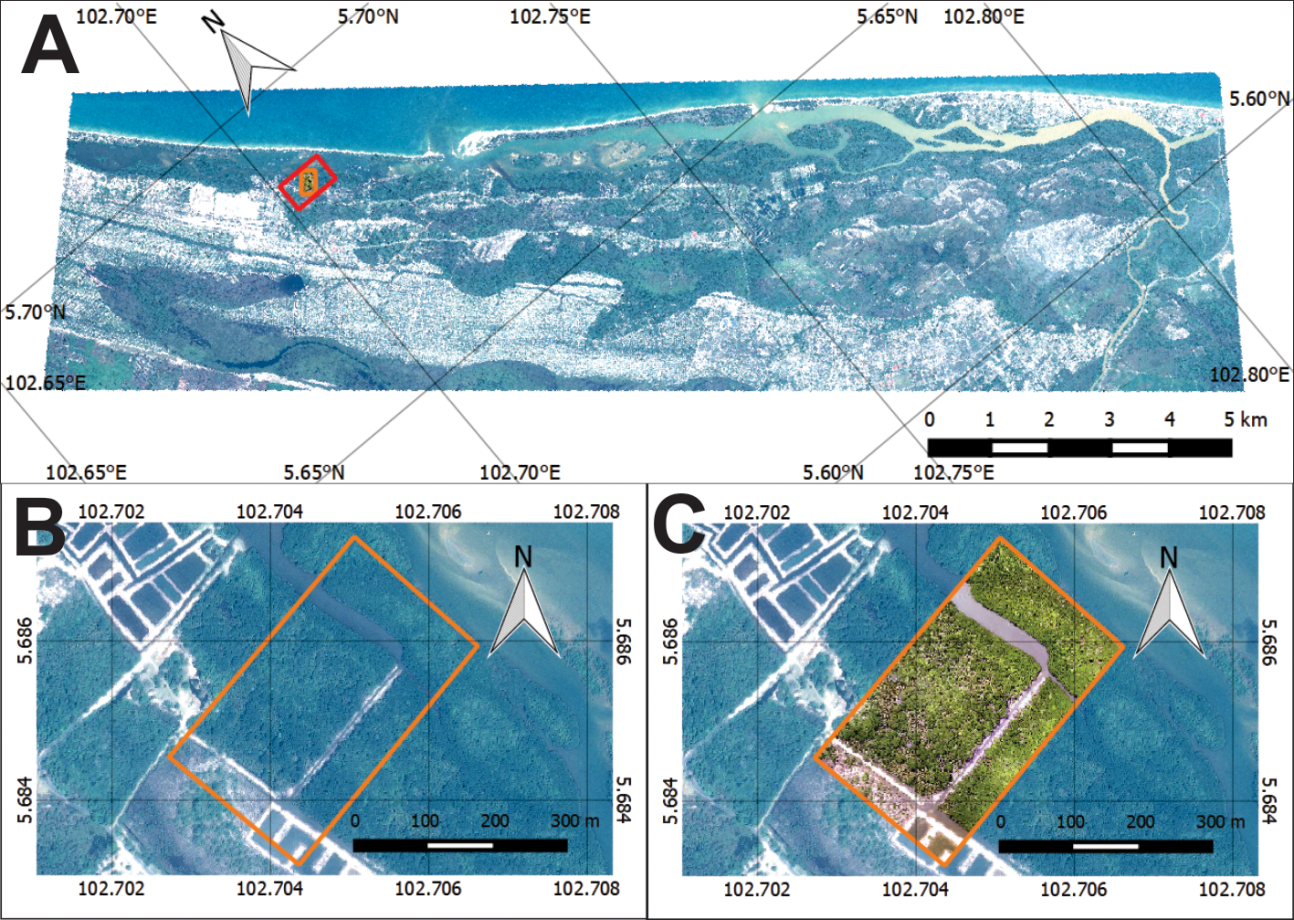
Research area in Setiu Wetland. (A) Setiu Wetland as shown by Pleiades-1B satellite imagery (acquired on 16 August 2013 at 11.43 AM). Red box indicates the location of mangroves being considered for vegetation mapping in the present study. (B) Pan-sharpened Pleiades-1B imagery (spatial resolution: 50cm) showing the zoomed-in portion of the mangroves selected for mapping (orange box). (C) DJI-Phantom-2 drone imagery (acquired on 3 July 2015 at 10.00AM) (spatial resolution: 5 cm) showingthe same mangrove coverage area as that of Pleiades for mapping (orange box) (background: Pleiades imagery).

Setiu Wetland is located in the State of Terengganu on the Peninsular Malaysia (05⁰36’30”-05⁰42’30”N, 102⁰40’30”-102⁰48’30”E) (Fig 1). The entire wetland covers 230km^2^ of non-mangrove wetland, 8.8km^2^ of water, and 4.18km^2^ of mangrove vegetation [50,51]. This ecosystem is represented by several coastal features, including beaches, sea, mudflats, lagoons, estuaries, rivers, islands, seagrass beds, and coastal vegetation, including mangroves [51]. The mangroves are composed of 23 true and 38 associate species [52]. Major mangrove species in this wetland include *Avicennia alba* Blume, *A*. *lanata* Ridley, *Ceriops decandra* (Griff.) Ding Hou, *Bruguiera sexangula* (Lour.) Poir., *B*. *gymnorrhiza* (L.) Lamk., *B*. *cylindrica* (L.) Blume, *Lumnitzera racemosa* Willd., *Rhizophora apiculata* Bl. and *Nypa fruticans* (Thunb.) Wurmb. [51], along with the critically endangered *B*. *hainesii* C.G. Rogers [7,16,53].

Setiu Wetland plays a significant role for local livelihoods, especially in terms of aquaculture and related activities [54,55]. The local communities visit the wetland regularly to collect clams (*e*.*g*. *Anadara* and *Placuna* spp.), crabs (*e*.*g*. *Scylla* spp.), and honey, and *Nypa* palm leaves for personal and commercial usage (pers. obs.). Currently, the local government is taking the necessary measures to declare this area as a State Park Reserve [56]. Of the 4.18km^2^ of mangrove cover at Setiu, the present analysis of vegetation mapping focused on an area of 0.12km^2^ (Fig 1) where the species composition varies, and could represent different spectral reflectance conditions. In recent years, the increase of oil palm plantations in the vicinity is believed to have decreased the freshwater input into this lagoon, especially on the northern side (Cik Azmi, village head, pers. comm.) where several muddy-sand areas with a suboptimal growth of the mangrove trees (height <3m) are noticeable (pers. obs.).

### Fieldwork

#### Ground inventory

The fieldwork was carried out in June-July 2015, under the permit from Institute of Oceanography and Environment as an authority which managed research on Setiu Wetland. (S4le). Plot-based (5×5m^2^) measurements were obtained from mangroves (from the waterfront to the terrestrial edge, based on a pre-determined grid), and the existing land-use/cover (*e*.*g*. *Casuarina*, *Pandanus*, aquaculture, building and other features) was recorded with camera and GPS. Altogether, 101 mangrove plots and 155 ground verification points were investigated from the entire wetland area. However, for the present paper, only the ground inventory details of the area that corresponded to drone data coverage/analysis were considered.

#### Aerial photos acquisition

**Fig 2:**
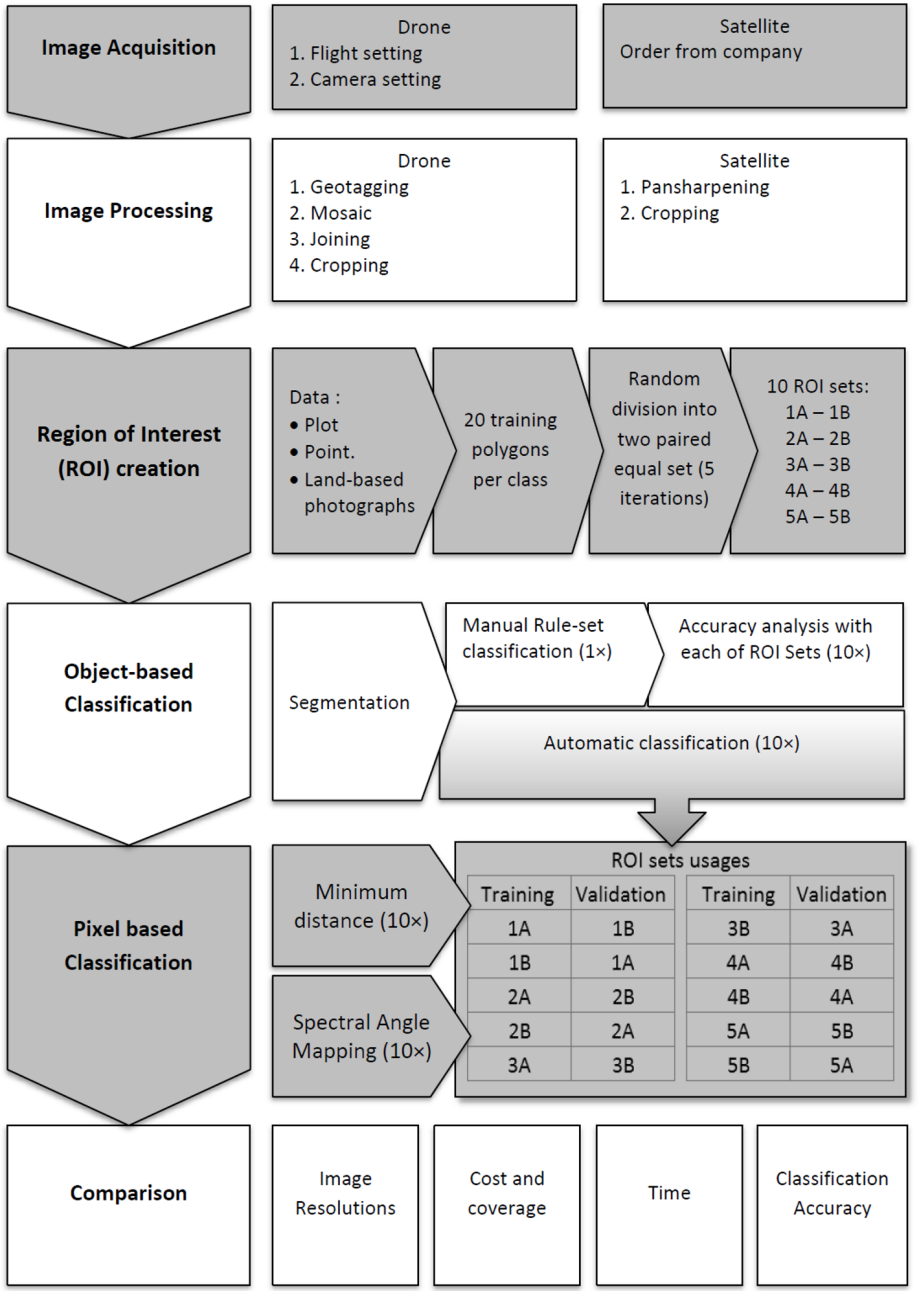
Stepwise protocol and the technical processes involved in drone and satellite remote sensing data analyses for mangrove mapping at the Setiu Wetland. The 10 ROI sets were named 1A-1B to 5A-5B. Except the manual rule-set algorithm, the remaining algorithms i.e., automatic, maximum likelihood and spectral angle mapping, were used 10 times (10×) for running the object- and pixel-based classification approaches (grey and white shades are for visualization purposes).

An overview of the remote sensing approaches, from mangrove aerial photos acquisition using a drone, to drone/satellite images processing/analysis and their results comparison, is represented in a schematic flowchart (Fig 2).

For aerial photography, a DJI-Phantom-2 drone and Zenmuse gimbal (to stabilize the camera) was used. Flight tracks at 100m altitude were planned in DJI Ground Station *v*.04 software, and uploaded through a DJI2.4G Datalink. Two cameras—a regular red-green-blue (RGB) SJ4000wifi (12megapixel, wide angle, focal length 24mm and CMOS sensor 22mm×36mm), and another infrared (IR) SJ4000 (modified by IRPro with 22Blu dual-pass-bands filter) were used. Besides the known spectral range of the CMOS sensor (i.e. 350–1100nm) [57], both RGB and IR cameras were tested again in the Laboratory of Photonics Research at the Vrije Universiteit Brussel using Spectro 320 of the Instrument Systems and Image J software [58]. As the CMOS sensor has no specific spectral range for individual RGB bands, we considered the RGB values of each pixel as its band values.

The IR camera was adjusted to take pictures through a fastest time lapse interval of 5s at 2.5m/s flying speed, while the RGB camera was adjusted for 2s at 4m/s flying speed. The difference in time lapse between IR and RGB cameras was due to model specifications/restrictions. External data logger, Flytrex Core 2, was installed to record the flight track information (i.e. altitude, geographic coordinates and time of acquisition) [59].

### Data processing

#### Drone images

Detailed image processing protocol is available in S2 File and protocol.io (https://dx.doi.org/10.17504/protocols.io.qh7dt9n). GPS log data was imported from the drone using Gpsvisualizer [60], the individual photos were tagged by synchronising camera time and flight track information in Geosetter [61]. The tagged images was georeferenced and mosaiced in Agisoft Photoscan [62], with at least five Ground Control Points (GCPs) of prominent features from Google Earth^TM^. The mosaic images were exported as a regular image with RGB values and Digital Elevation Model (DEM). The coordinate reference system was assigned to EPSG:32648 WGS84/UTMzone48N. The produced RGB, IR and DEM images were further rectified using Quantum Geographic Information System (QGIS *v*.2.12.3-Lyon software), with 12 GCPs from satellite image and visual observations based on field work. The images were then merged into a single image with seven bands (Table 1). In addition, edges of the image affected by the parallax effect (showing tall objects as elongated with invalid DEM) were cropped out.

**Table 1:** List of bands in a RGB, IR and DEM merged imagery of the DJI-Phantom-2 drone.

Band number	Source camera	Information	Digital value	Unit
Band 1	RGB	DEM	-10–30	Meter
Band 2	IR	NIR	0–255	8-bit colour value
Band 3	IR	NIR	0–255	8-bit colour value
Band 4	IR	NIR	0–255	8-bit colour value
Band 5	RGB	Red	0–255	8-bit colour value
Band 6	RGB	Green	0–255	8-bit colour value
Band 7	RGB	Blue	0–255	8-bit colour value

#### Satellite imagery

A Pleiades-1B image of the Setiu Wetland purchased and authorized by University of Malaysia Terangganu (S3 File), which is dated on 16 August 2013 with the spatial resolution of 2m –duly corrected by Astrium Services (the image distributor company) for radiometric and sensor distortions–was used for the present study. This multispectral image, covering 100km^2^, was first pan-sharpened in QGIS (via Ratio Component Substitution (RCS) algorithm), and then cropped to generate the same mangrove coverage area as that of the drone imagery for comparison of the results.

#### Region of Interest (ROI) creation

The ROIs for land cover training sites and classification accuracy assessment were generated based on the ground-truthing data (using Semi-Automatic Classification Plugin (SCP) in QGIS) [63]. Three ROI groups—one for Pleiades, and two for drone image—were considered for the classification. While two ROI groups—one meant for Pleiades and another meant for the drone, contained the same number of (dominant) land-cover classes, the third ROI group of drone images has extra classes representing other visible (non-dominant) features on the ground. According to Mather (2004) [64], the number of pixels per land-use/cover class selected for training sites must be at least 30 times the spectral dimension. Therefore, we selected 360 pixels per class in Pleiades. However, in the case of higher resolution drone data, each corresponding class was represented by 36,000 pixels. All training sites were then separated into 20 polygons of the same size in both images. By random selection, 50% of the polygons in each class were selected as training sites. The remaining 50% unselected polygons were used for accuracy assessment. This random selection was repeated to create at least five paired ROI sets (named after 1A-1B to 5A-5B as shown in Fig 2), resulting in ten iterations of cross validation. [65,66].

#### Object-based classification

Both the Pleiades and drone images were segmented by Large-Scale Mean-shift Classification (LSMC) region growing algorithm [67,68] in QGIS (Orfeo toolbox). The vector segments were classified based on OpenGIS simple features reference implementation (OGR) classifier with a Support Vector Machine (SVM) algorithm, as well as a Manual rule-set (MAN) classification. While OGR is a free automatic (AUT) application to run the classification [67], the MAN is based on signature details and spectral distances, together with visual interpretation. The MAN was applied to assign a class for each object/segment, and produced the classification. With the AUT, both Pleiades and drone images were classified ten times by each ROI set, but only once using the MAN (Fig 2). The classified images were exported into raster files for accuracy assessment.

#### Pixel-based classification

Pixel-based classification was carried out in SCP QGIS using the ROI training sites. Since the accuracy of a handheld GPS (Garmin 45, USA) is *ca*. 5–6 m, field data were superimposed on the drone imagery, and visually checked for authenticity of the training sites. After the classification attempts using three algorithms i.e., Minimum Distance, Spectral Angle Mapping (SAM), and Maximum Likelihood (MLI), the results obtained from SAM and MLI were found to be appropriate for this study [63,69]. Both Pleiades and drone images were classified ten times by each ROI set under the MLI and the SAM classification scenarios (Fig 2), and exported for accuracy assessment.

#### Accuracy assessment

Accuracy analysis in the form of an error matrix was generated by comparing the classified (object- and pixel-based) images against the paired ROIs as cross validation (using SCP QGIS). For instance, the image classified by ROI 1A was validated by ROI 1B and *vice versa* (Fig 2) [65,66]. Based on this error matrix, overall accuracy (OA) and Kappa index were derived for the entire classification of each image; and specific producer accuracy (SPA) and specific user accuracy (SUA) for each land-cover class [63,69]. The error matrix was also incorporated into the Pontius Matrix to estimate ‘quantity’, ‘exchange’ and ‘shift’ parameters for identifying the source of classification error [70,71]. While ‘quantity’ (%) represents the amount of pixels that differed between training sites and classification per class, ‘exchange’ (%) shows the allocated error by the number of pixels that interchanged between two classes, and ‘shift’ (%) denotes the other allocation differences that were not included in the quantity and exchange differences [71].

### Results comparison

The capability of Pleiades-1B and DJI-Phantom-2 was evaluated through their image quality in terms of spatial, spectral, radiometric and temporal resolution; efficiency in terms of area coverage, data acquisition/processing time and user-cost, and accuracy in terms of object- and pixel-based classification approaches. Results of the accuracy assessment (i.e., OA, Kappa, SPA, SUA and Pontius Matrix—quantity, exchange and shift) were represented by boxplots using the R-Studio [72]. Statistical variations among the classification accuracies and approaches were identified through Kruskal-Wallis and Mann-Whitney tests using Past *v*.3.14 software [73].

## Results

### Image quality

**Fig 3:**
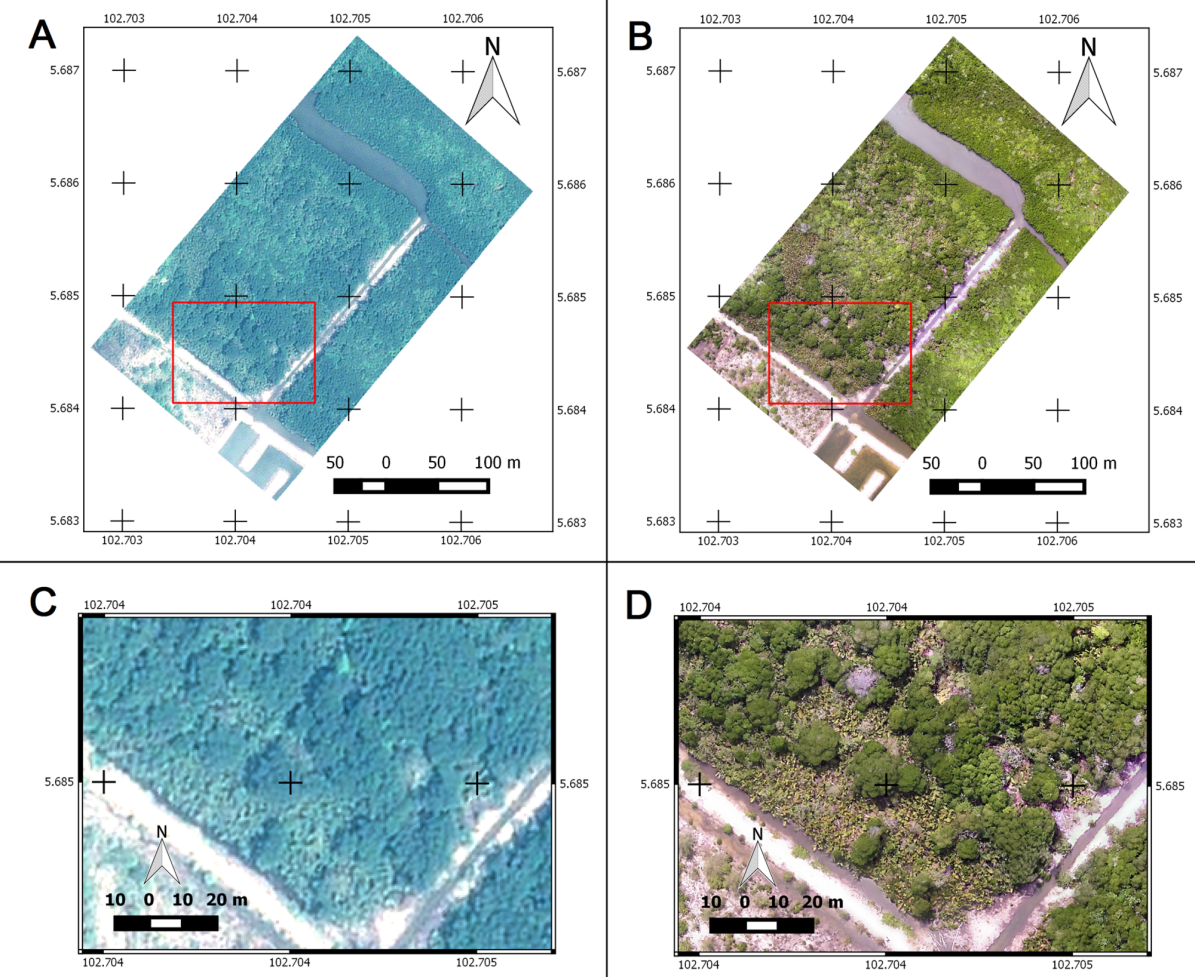
Comparison of cropped true colour composite images from Pleiades-1B satellite and DJI-Phantom-2 drone. (A-B) The images for mangrove vegetation mapping at the Setiu Wetland. Red boxes show the zoomed-in subsets of–(C) Pleiades-1B and, (D) DJI-Phantom-2 drone, revealing mangrove and non-mangrove details on the ground at 50cm and 5cm spatial resolutions respectively.

The drone image had a higher spatial resolution compared to the pan-sharpened Pleiades image (2.8cm vs 50cm, Table 2 & Fig 3). In term of spectral resolution, the satellite image performed better due to specific sensors with a definite wavelength. Although spectral range of the drone cameras were tested (RGB = 450–675nm and IR = 875–1100nm), each specific band wavelength was not known. Pleiades has a higher radiometric resolution compared to drones (12bit vs. 8bit). With a daily revisit schedule of the Pleiades satellite, the data is available for every day. In addition, a drone can deliver the aerial photos daily, but not under rainy or stormy conditions.

**Table 2:** Spatial, spectral, radiometric and temporal resolutions of the Pleiades-1B satellite and DJI-Phantom-2 drone images (source for Pleiades-1B information: Pleiades user guide [74]).

	Spatial resolution (cm)	Spectral resolution (nm)	Radiometric resolution	Temporal resolution
**Pan-sharpened Pleiades imagery**	50	Blue: 430–550 Green: 500–620 Red: 590–710 NIR: 740–940	12-bit (0–4095)	Daily
**DJI-Phantom-2 drone imagery:**				
i) Red-Green-Blue (RGB) bands (SJ4000wifi camera)	2.8	450–675	8-bit (0–255)	Daily if no rain/storm
ii) Infrared (IR) (SJ4000 camera)	3.5	875–1100	8-bit (0–255)	
iii) Digital Elevation Model (DEM)	10	-	-	

### Data efficiency

#### Cost and coverage

**Fig 4:**
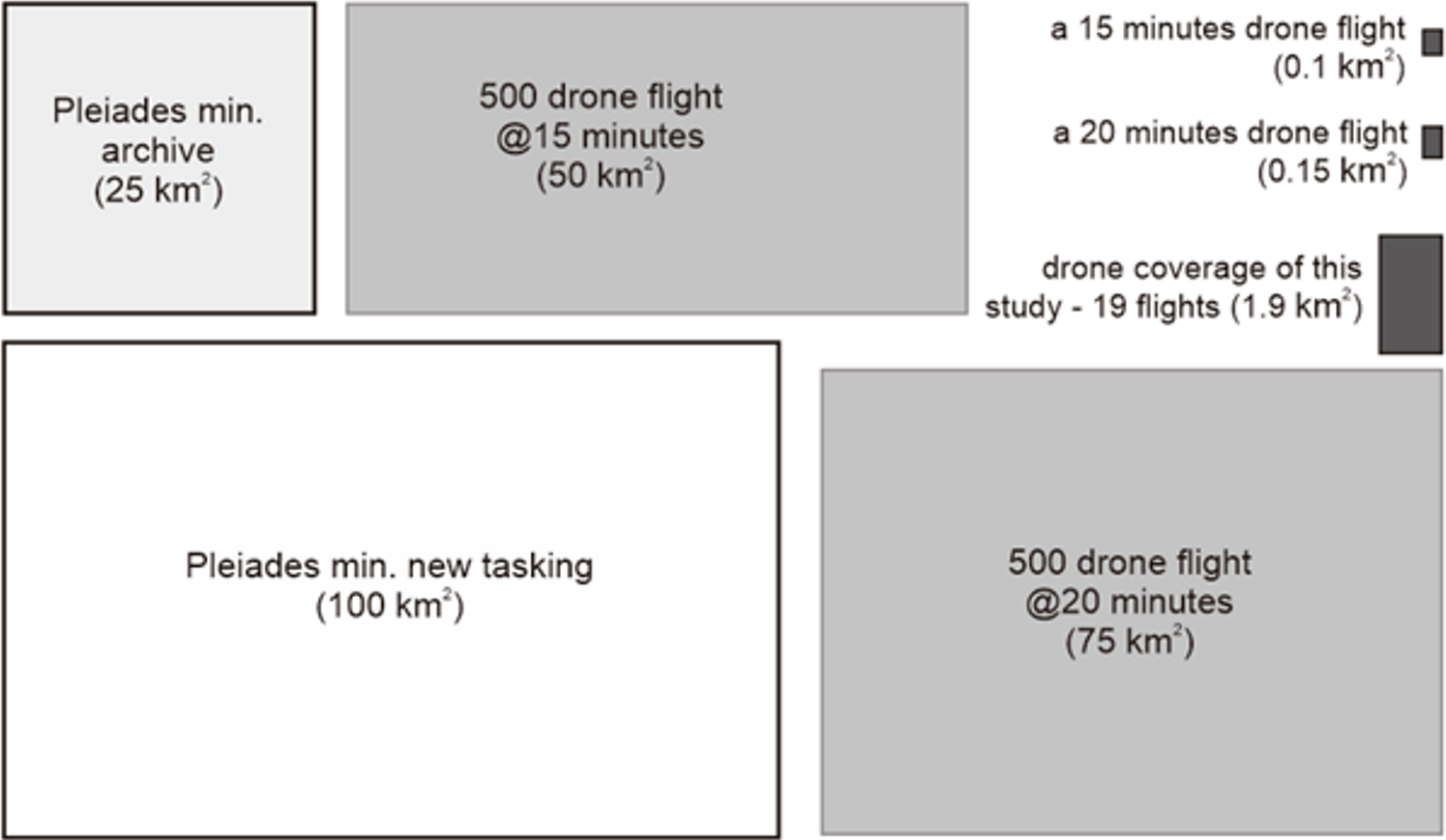
Visual representation of the spatial coverage of Pleiades-1B and DJI-Phantom-2 drone data sets. While new tasking/purchasing order of Pleiades images requires at least 100km^2^ coverage, the archived data of each image is available for a minimum of 25 km^2^. A drone is expected to work efficiently (if it does not crash or have technical problems) for 500 flights. If an efficient drone flight for 15 min (corresponding to average battery run-time) can cover approximately 0.1 km^2^, the total drone flights would be able to cover *ca*. 50 km^2^. However, with an improved battery run-time of up to 20 min these days, the same drone can deliver aerial photos of an area covering up to 75 km^2^ (box dimensions are arbitrary, and the colours are for visualization purposes only).

The cost of the DJI-Phantom-2 drone (with an RGB SJ4000 camera) used in this study was 950USD (April 2015). In addition, the cameras and other accessories (gimbal, GPS logger, batteries) cost 1003USD. Together with the fieldwork expenses of 500USD, the total budget spent was 2453USD. On the other hand, the Pleiades-1B imagery was procured for 1750USD. Out of the two months’ fieldwork, two weeks were focused on the aerial photos acquisition, and obtained 19 composite (RGB, IR and DEM) images, covering area of 1.81km^2^ (Fig 4). If the mangrove surface area being covered in the present aerial photos is considered, then the drone data acquisition cost was *ca*. 1355USD per km^2^ (2453USD ÷ 1.81km^2^), which is more expensive than the Pleiades-1B satellite data (17.5USD per km^2^). The Pleiades data is still economical, even after adding the cost of present fieldwork expenses (22.5USD per km^2^). Although each of our 15 minutes drone flights, corresponding to the average battery run-time at a given speed/altitude, typically covered 0.12km^2^, the parallax cropping reduced it to 0.09–0.11km^2^ (Fig 4).

#### Image acquisition and processing time

Under emergency conditions, the Pleiades satellite data is available to the user within 24 hours. Drone aerial photos can also be acquired daily under favourable weather conditions. The mangrove area of 0.12km^2^ chosen for the present mapping study was represented by 2,800 and 12MB sizes of drone and Pleiades images respectively. Due to the large data size of the drone image (*ca*. 230 times greater than satellite data), its processing time—especially in the case of pixel-based classification, was found to be ten times greater than the satellite image (Table 3). Meanwhile, segmentation process in the object-based classification has greatly reduced the drone imagery size (from 2,800 into 194MB) as well as the processing time.

**Table 3:** Data processing time of DJI-Phantom-2 drone and Pleiades-1B satellite images. For the drone, the accuracy analysis of pixel-based classification was conducted twice due to there being two different training sites–one representing dominant land-use/cover classes, and another representing both dominant and non-dominant classes visible on the ground.

	Time taken for drone imagery(h)	Time taken forsatellite imagery.(h)
**Image preparation**		
Geotagging	2	-
Mosaicking	8	-
Joining	6	-
Pansharpening	-	0.5
Cropping	0.5	0.1
**ROI creation**		
Data interpretation	1	1
Creating random polygons	2	1
Creating random sets	2	1
**Object-based classification**		
Segmentation	4	1
Object based automatic classification (10 iterations)	2	1
Manual rule-set classification (1 iteration)	2	1
Dissolve segment	6	1
Convert to raster	2	1
Accuracy analysis (10 iterations)	2	1
**Total time**	**18**	**6**
**Pixel-based classification**		
Maximum Likelihood (10 iterations)	10	1
Spectral Angle Mapping (10 iterations)	10	1
Accuracy Analysis (20 iterations)	30	3
**Total time**	**50**	**5**
**Total time for entire analyses**	**89.5**	**14.6**

### Data classification

#### Land-cover categories

**Fig 5:**
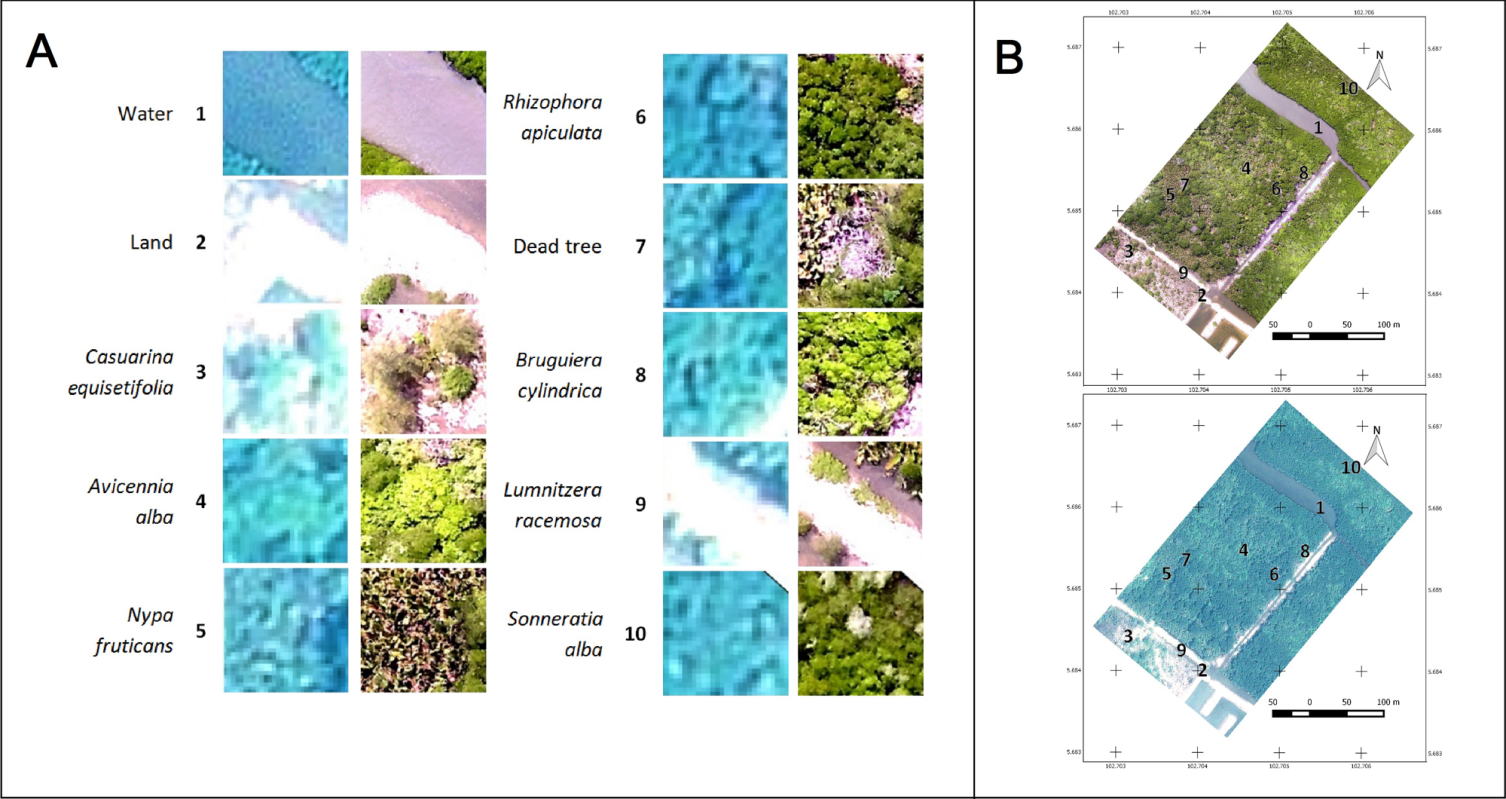
Land-cover classes at the Setiu Wetland. (A) Land-cover classes based on Pleiades-1B satellite (on the left) and DJI-Phantom-2 drone (on the right). Due to the poor demarcation of some features in the Pleiades, only 1–6 land-cover classes were considered for its image classification. (B) Locations of the ten (1–10) land-use/cover classes marked on the Pleiades-1B (top) and DJI-Phantom-2 drone (bottom) images.

Based on the visual interpretation and the ground-truthing, it was possible to distinguish six dominant (*i*.*e*. water, land, non-mangrove *Casuarina equisetifolia*, and mangrove species *A*. *alba*, *N*. *fruticans* and *R*. *apiculata*) and four non-dominant (*B*. *cylindrica*, *L*. *racemosa*, *S*. *alba* and dead trees) land-cover classes at Setiu. *R*. *apiculata* and *A*. *alba* spectral signatures show relatively high homogeneity, while the *N*. *fruticans* signature shows the highest heterogeneity. While the dominant classes are visually clear in both Pleiades and drone images, the non-dominant classes are best observed in the drone imagery (Fig 5).

#### Image classification and accuracy

Besides the classification of drone and satellite images for dominant land-cover classes, the higher resolution drone images were additionally classified for both dominant and non-dominant classes (Table 4). The object-based (with AUT and MAN algorithms) and the pixel-based (with MLI and SAM algorithms) classification approaches produced 93 maps in total, of which the ones with the best OA were used for the present publication (Table 4). The OA, Kappa, SPA and SUA values of all object- and pixel-based classification iterations and the error matrix of each classified map used in this publication are available in the S1 parts 1 and 2.

**Table 4:** Details of the classification approaches and resultant maps (with accuracy iterations) using DJI-Phantom-2 drone and Pleiades-1B satellite images for the Setiu Wetland. Each classified map was given a unique identification code that starts with ‘D’ for drone and ‘S’ for satellite, followed by a number of the land-use/cover classes used (10 = all ten land-use/cover categories and 6 = dominant six classes), classification approach (O = object-based and P = pixel-based), and the algorithm (MAN = Manual rule-set, AUT = Automatic, MLI = Maximum Likelihood and SAM = Spectral Angle Mapping) (OA = Overall Accuracy) (ROI = Region of Interest) (*training site codes follow those used in Fig 2).

Image source	No. of class-es	Classification approach	Classification.Algorithm	Abbre-viation	No. of classification iterations	No. of accuracy iterations	*ROI training sites that produced the highest accuracy maps and its OA	Mean and standard deviation of each classification method
Drone	10	Object-based (O)	Manual rule-set (MAN)	D10OMAN	1	10	4B(71.8%)	69.7±1.2%
(D)			Automatic (AUT)	D10OAUT	10	10	5A(68.7%)	65.8±3.4%
	10	Pixel-based (P)	Maximum Likelihood (MLI)	D10PMLI	10	10	4A(79.4%)	77.6±1.3%
			Spectral Angle Mapping (SAM)	D10PSAM	10	10	3B(55.0%)	51.8±1.6%
	6	Object-based (O)	Manual rule-set (MAN)	D6OMAN	1	10	2B(94.8%)	94.0±0.5%
			Automatic (AUT)	D6OAUT	10	10	5A(85.9%)	78.4±5.3%
	6	Pixel-based (P)	Maximum Likelihood (MLI)	D6PMLI	10	10	2A(93.0%)	90.0±1.9%
			Spectral Angle Mapping (SAM)	D6PSAM	10	10	3B(76.0%)	72.0±1.9%
Satellite	6	Object-based (O)	Manual rule-set (MAN)	S6OMAN	1	10	5A(77.8%)	72.2±2.7%
(S)			Automatic (AUT)	S6OAUT	10	10	1B(75.9%)	70.8±8.1%
	6	Pixel-based (P)	Maximum Likelihood (MLI)	S6PMLI	10	10	3B(88.3%)	82.8±3.5%
			Spectral Angle Mapping (SAM)	S6PSAM	10	10	4B(77.3%)	73.3±5.1%

**Fig 6:**
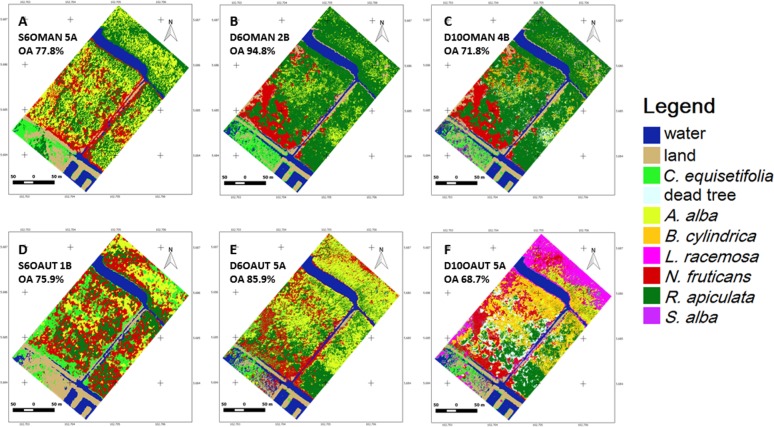
The Setiu Wetland mangrove maps based on object-based classification. Masp based on Manual rule-set algorithm (A-C) and the Automatic classifier algorithm (D-F). The images shown have the highest Overall Accuracy from 10 iterations. (Abbreviations for each image follow the map identification codes in Table 4. OA = Overall Accuracy. Genus names: *A* = *Avicennia*, *B* = *Bruguiera*, *L* = *Lumnitzera*, *N* = *Nypa*, *R* = *Rhizophora*, *S* = *Sonneratia* and, *C* = *Casuarina*.).

**Fig 7:**
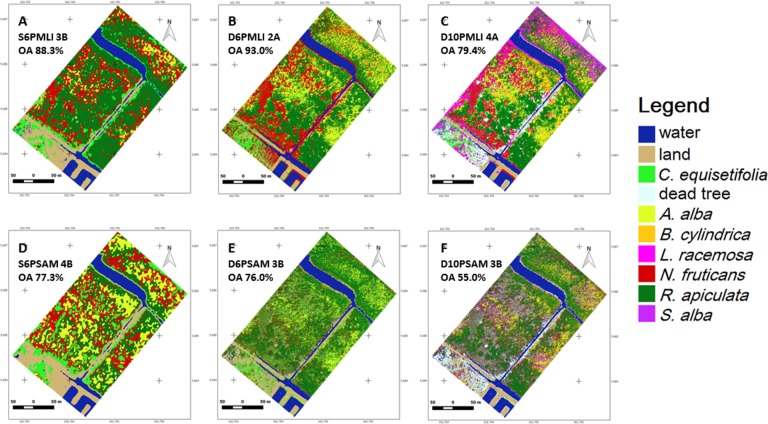
The Setiu Wetland mangrove maps based on pixel-based classification. Map sbased on Maximum Likelihood algorithm (A-C) and the Spectral Angle Mapping algorithm (D-F). The images shown have the highest Overall Accuracy from 10 iterations. (Abbreviations and genus names follow those used in Fig 6).

**Fig 8:**
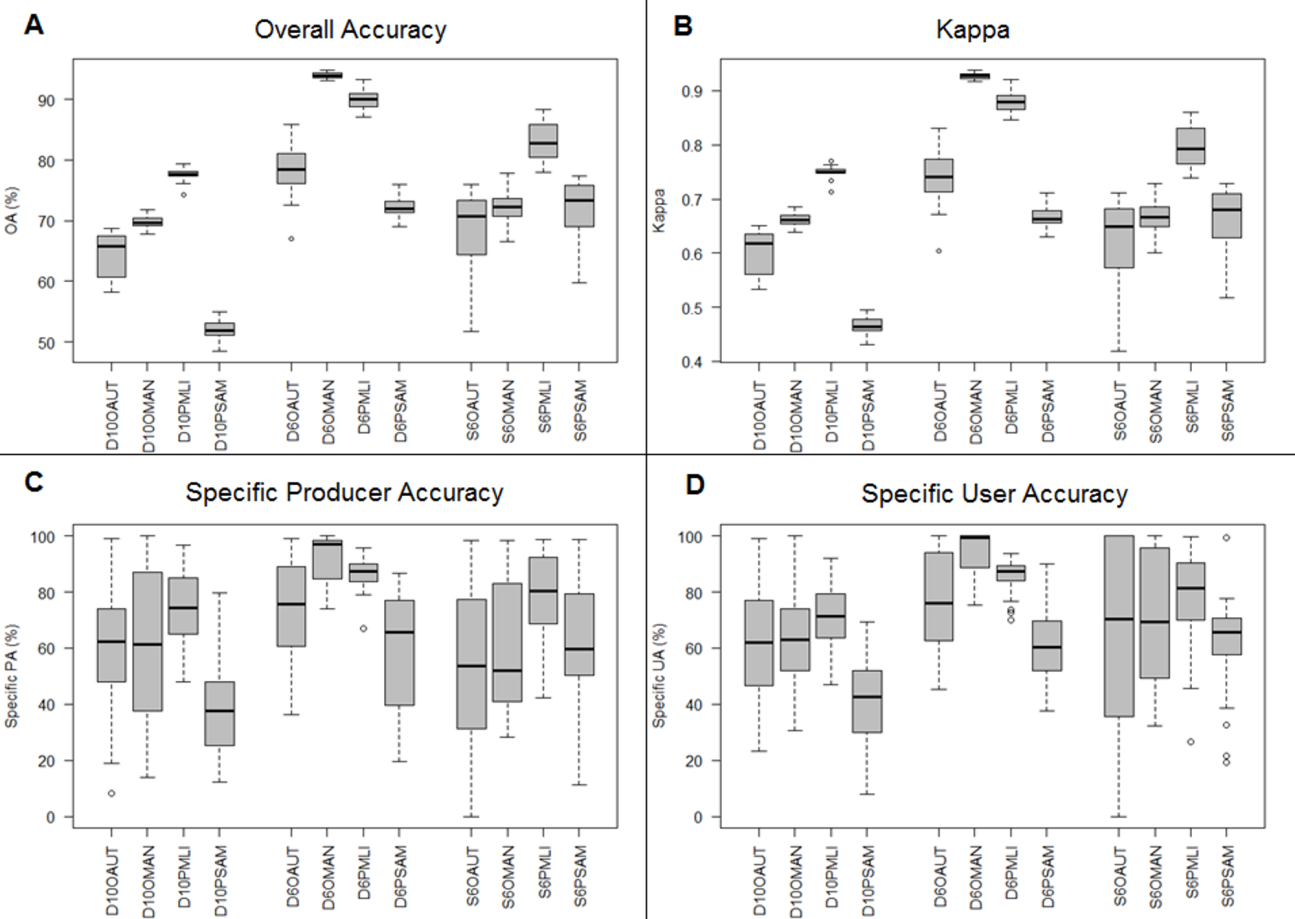
Accuracy analysis of the mangrove vegetation mapping at the Setiu Wetland, based on DJI-Phantom-2 drone and Pleiades-1B satellite images. (A) Overall Accuracy, (B) Kappa Index, (C) Specific Accuracy and, (D) Specific Reliability. Each abbreviation along the X-axis follows the map identification codes in Table 4.

For the object-based classification, the MAN algorithm provided more accurate results than AUT (Fig 6), whereas for the pixel-based classification, MLI was more competent than SAM (Fig 7). The drone image classified through MAN for six dominant land-cover classes (D6OMAN) provided a higher OA (94.0±0.5%) in contrast to the Pleiades (S6OMAN: 72.2±2.7%) or the drone with ten classes (D10OMAN: 69.7±1.2%) (Table 4 & Fig 8). In addition, in the case of pixel-based classification, the drone image classified through MLI for dominant land-cover classes (D6PMLI) provided good accuracy (90.0±1.9%) as compared to the Pleiades (S6PMLI: 82.8±3.5%) or the drone with ten classes (D10PMLI: 77.6±1.3%) (Table 4 & Fig 8).

Both AUT (for object-based classification) and SAM (for pixel-based classification) algorithms provided low (D10PSAM: 51.8±1.6%) to moderate accuracy maps (D10OAUT: 65.8±3.4%; S6OAUT: 70.8±8.1%; D6PSAM: 72.0±1.9%; S6PSAM: 73.3±5.1%), except for the drone imagery that was classified through AUT for dominant classes (D6OAUT: 78.4±5.3%) (Table 4 & Fig 8). While there was an inconsistent performance of the MAN algorithm (D6OMAN, S6OMAN, D10OMAN), the MLI showed rather high OA, Kappa, SPA and SUA values (D10PMLI, D6PMLI, S6PMLI) (Fig 8). Among others, the higher threshold values of OA and Kappa were confined only to the drone image classified through the MAN (D6OMAN) and MLI (D6PMLI) algorithms. Overall, the statistical variations among these classification accuracies and approaches were found to be significant (S1 part 1)

**Fig 9:**
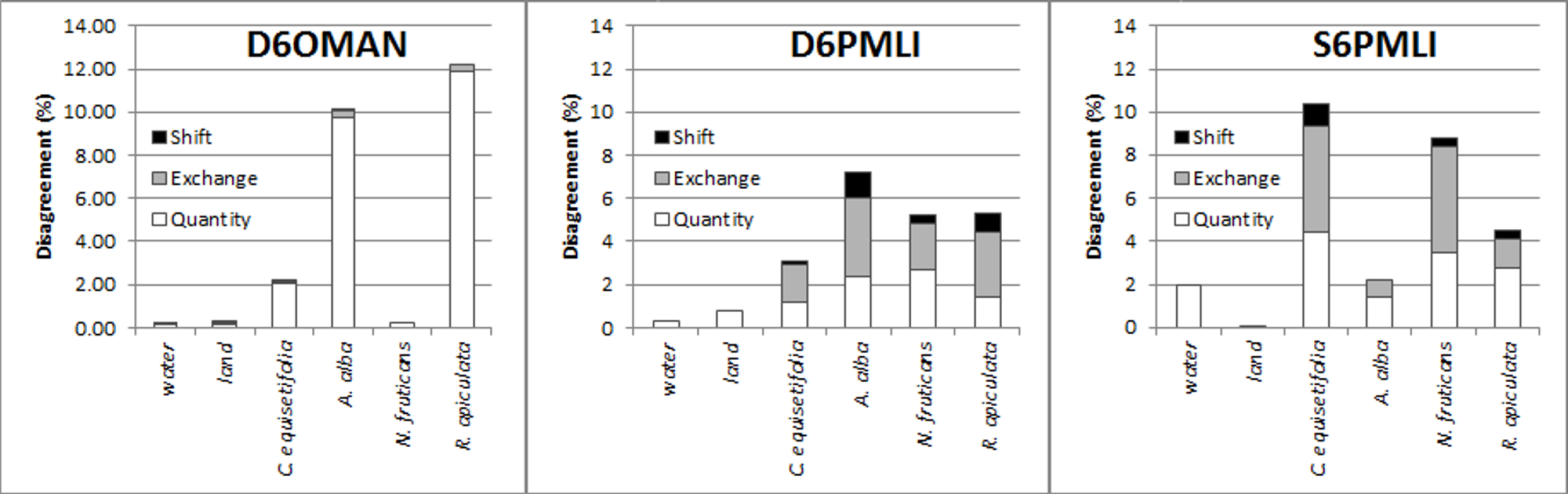
Mean shift (black), exchange (grey) and quantity (white) differences in the first three maps with highest overall accuracy. Result based on DJI-Phantom-2 drone (A-B) and Pleiades-1B satellite (C) images for the Setiu Wetland. Abbreviations for each image follow the map identification codes in Table 4. Abbreviations follow the identification codes in Table 4.

In terms of the classification errors, identified from the first three highest OA observed land-cover maps (Fig 9), the drone image subjected to object-based classification through MAN for dominant classes (D6OMAN) showed an overestimation of *R*. *apiculata* and *A*. *alba*. In the case of pixel-based classification, both drone and satellite images classified through MLI for dominant classes (D6PMLI and S6PMLI) had their classification errors spreading among the various vegetation classes.

## Discussion

### Image quality

The potential of drone imagery over space-borne imagery for mangrove species-level mapping was evident from this study. However, Pleiades (and also other space-borne data) has the advantage of having a better spectral resolution—useful for indicating health and biomass of the vegetation [15,17], which is not available for the drones using a normal RGB camera (Table 2). Since the SJ4000 camera used in this study was fixed with auto-exposure, the conversion of digital numbers into calibrated radiance values was not feasible [75]. Therefore, some of the image processing techniques like image overlay analysis or batch-wise classification, applicable to satellite data, are not supported. While having experience with visual interpretation of remotely sensed imagery [36], and despite not having applied this technique in the present paper, we believe that drone imagery offers an entirely new and promising suite of possibilities to unambiguously identify and distinguish mangrove genera and species (including congeneric species and maybe even subspecies and varieties). This is evidenced by the superior quality of drone imagery (Figs 3 and 5) and the possible application in studies that do not aim at mapping, but rather at pinpointing species or individuals of interest, for instance to recognise invasive species [24] or cryptic ecological degradation [22], to identify tree-top or branch die-off [76] or to quantify biomass loss resulting from tree fall or lightning strikes [77].

According to Tucker (1980) [78], there was only a 2–3% (insignificant) improvement of radiometric resolution in between 6, 8 and 9-bit data of the Landsat. Though a higher radiometric resolution (*e*.*g*. 12-bit) that comes with the VHR data is useful for mapping the shaded areas (*e*.*g*. shaded mountain flanks) [79], it seems less advantageous for mangroves. Despite the daily availability of Pleiades images, the cloud cover (including its shade) in tropical coastal areas reduces the image quality and makes the images unsuitable for mangrove research [15,33]. Although there was no such limitation for drone aerial photos, weather conditions without rain and storm risks are mandatory. Despite the fact that some wild and trained birds of prey are known to attack drones [80,81], we did not face such problems. Although some wild birds were flying around during the fieldwork, they tended to avoid the drone.

Perhaps the development of waterproof drones in the near future can enhance the window time for drone imagery [82]. Based on our field experience, the good quality drone aerial photos for mangroves, without sun gleam resulting from water reflection, can be obtained when the sun’s angle is less than 20° from the horizon (i.e., 1 hour after sunrise or 1 hour before sunset). In this context, sunny days during times of low tide (without tidal inundation below the canopy) and cloudy days (as the sun’s glare is prevented) are also useful.

### Data efficiency

Archived data of the Pleiades (available for min. 25km^2^ purchase) are economical, but cloud-free images for some locations like Setiu Wetland were scarce. On the other hand, new tasking for these satellite images is expensive due to the requirement of a minimum 100km^2^ (23USD per km^2^) purchasing order (Fig 4). There are low cost drones like the Quanum Nova (*ca*. 275USD) or homemade do-it-yourself (*ca*. 100USD), but their image quality is compromised [83]. Theoretically, a drone is expected to work efficiently if the battery–which can last up to 300 recharging cycles (DJI, 2014)—is in good condition, or until the instrument crashes or suffers technical problems. If the drone’s life is estimated at 500 flights (using two batteries) and each flight for 15–20min can deliver aerial photos covering an area of 0.1–0.15km^2^, then the total area covered by a drone could be *ca*. 50–75km^2^ (Fig 4). This could even reduce the cost of drone image acquisition to 32-49USD per km^2^ (2453USD ÷ 50–75 km^2^). Although the Setiu mangrove area mapped in this study is rather restricted (0.12km^2^), it has proven to be good enough for showing the potential of drone data. Earlier, Lucieer et al (2010) [45] demonstrated Antarctic moss beds drone mapping in a 200m×200m area, and Ventura et al (2016) [83] for coastal fish nursery grounds mapping in 60m×80m area on Giglio Island, Italy.

For mangrove research, despite it being a basic version, the DJI-Phantom-2 drone was found to be efficient and fit for purpose. However, if the budget is not a constraint, the Phantom 2 could be replaced with other updated and better performing models equipped with different camera sensors (Table 5). The newer UAV models also have an improved battery run-time up to 20–25minutes, and can cover larger areas at the same altitude and speed settings. For instance, an increase of 5minutes in drone flight time would allow an increase of 4ha coverage in each image [82]. Limits in drone coverage at Setiu (1.81km^2^) were due to our need to learn flight operations, as well as there being no facility to setup both RGB and IR cameras on-board on the Phantom 2 drone (each flight path was covered twice by changing the cameras). With the reduced cost of UAVs, the aerial photos acquisition is becoming more economical, although the multispectral data remain expensive (Table 5). Overall, drone equipment is a one-time investment, and the long-term monitoring of any area could lead to the generation of cost-effective data.

**Table 5:** Various types of drone equipment, camera choices and sensor combinations, with applicable prices, useful for mangrove vegetation mapping (source: DJI Store [84], Specsheet Sequoia [85]) JI, 2017; Parrot, 2016).

Equipment	Price.(USD)		Possible sensor combinations	Purchasing price (USD)	Price per km^2.^(USD)
**Drone type**			**With RGB camera**		
DJI-Phantom-3	499		DJI-Phantom-3 with original camera	999	13.9
DJI-Phantom-4	1200		DJI-Phantom-4 with original camera	1700	23.6
Parrot-3DR Solo	1200		Parrot-3DR Solo + SJCAM	1770	24.6
**Camera type**			**With IR camera**		
SJCAM	70		DJI-Phantom-3 + IRPRO camera	1549	21.5
IRPRO-GoPro	550		DJI-Phantom-4 + IRPRO camera	2250	31.3
Parrot-Sequoia	3500		Parrot-3DR Solo + IRPRO camera	2250	31.3
**Operational cost**	500		**With multispectral camera**		
			DJI-Phantom-3 with Parrot-Sequoia	4499	62.5
			DJI-Phantom-4 with Parrot-Sequoia	5200	72.2
			Parrot-3-DR Solo with Parrot-Sequoia	5200	72.2

Pleiades has a faster data processing time than the DJI-Phantom2 (Table 3). However, the segmentation process in object-based classification has reduced the drone data size as well as its processing time (50h for pixel-based, 18h for object-based). On the other hand, segmentation did not benefit the processing time of Pleiades (which took 6h for object- and 5h for pixel-based classifications). Perhaps the increased computing power and simplified drone data will speed up the image processing time in the future.

Finally, we recognise that some people might question why anyone would go through the trouble of flying a drone for weeks (largely depending on weather conditions and battery time), plus needing much time for the extra-long processing time, if a single satellite image can lead to a similar result. When answering this question, one should consider that satellite imagery is not always available, and where it is not, drone imagery is a much cheaper alternative if the purpose is to collect VHR imagery. The eventual choice will be dictated by logistics, weather, and field conditions, and technological progress. With this work, we are offering insights into comparable options that can lead to more informed choices.

### Image classification and accuracy

Since the MAN algorithm in the object-based classification is highly subjective (with a rule to classify the segments based on signature details, spectral distances and visual interpretation), the differentiation of mangrove species like *R*. *apiculata* and *N*. *fruticans* was difficult for Pleiades imagery (Fig 6). In fact, visual interpretation of *N*. *fruticans* can reveal its rough texture, dark shadow parts between fronds, and high reflectance of fronds (Fig 5), but this species often showed a high spectral signature similarity with other species, especially *R*. *apiculata*. As the AUT classification approach in QGIS is still in the experimental phase [67], its possible improvement in the near future is likely to provide more accurate results [86,87]. Concerning the pixel-based classification (Fig 7), the spectral angle of SAM represented a close similarity among land-cover types compared to the spectral distance of MLI, and hence delivered the maps with poor mangrove species’ discrimination. Low accuracy of the SAM classified maps was reported previously by Shafri et al. (2007)[88], Castillejo-González et al. (2009)[89] and Khatami et al. (2016)[90].

Higher threshold values of OA and Kappa index (Fig 8) encountered for D6OMAN and D6PMLI signify the capability of drone imagery over the satellite data, to produce both object- and pixel-based classifications with unprecedented accuracy, and fulfil the consensus criteria of land-cover mapping [91,92]. Although the Kappa index was proven to be misleading and ineffective for classification accuracy assessment [70], it showed a consistent pattern with the OA in the present study. Due to there being no marked variations or changes in the vegetation between the drone and Pleiades images (except some dead trees), we believe that the time gap (nearly 2 years) between these two data sets had a negligible impact on the image classification. On the other hand, classification errors in the images were due largely to overlapping vegetation spectral signatures (Fig 9). The DEM, which could represent tree height variations on the ground, especially between *N*. *fruticans* (height: 4–7m) and *R*. *apiculata* (16–19m), *A*. *alba* (10–13m) or *C*. *equisetifolia* (14–23 m) benefited the object-based classification (D6OMAN) more than the pixel-based classification (D6PMLI and S6PMLI). Higher classification accuracy was retained chiefly with the higher spatial resolution data, and this confirms the advantage of Phantom 2 drone imagery over the Pleiades for mangrove mapping at the Setiu Wetland. Moreover, the cross validation through a combination of different training sites and several iterations ensures no bias in the results produced.

This research was performed in a species-rich region (including adjacent non-mangrove sections) if compared to the global mangrove range. Hence, it was demanding in terms of ground truthing and image identification. Despite the rather homogeneous spectral signatures that we obtained in the present study for most of the mangrove species present, we recognise that the higher the image’s spatial resolution becomes, the higher the risk of obtaining very heterogeneous spectral signatures. Pixels originating from higher, lower, shaded, and sunny sides of the canopy might contribute to an overall heterogeneous spectral signature for one species, particularly if the texture of the crown is complex, as is the case with Nypa. Our other research shows that a lower spatial resolution does indeed increase the classification accuracy for this species (S1 part 5). However, for other trees, i.e. Rhizophora and Avicennia, a higher spatial resolution results in a higher classification accuracy.

## Conclusions

The present study revealed the potential of DJI-Phantom-2 drone aerial photos for mangrove mapping, as well as its capability against Pleiades-1B satellite data, from observations in the Setiu Wetland, Malaysia. Acquisition of drone data on cloudy days is exceptionally beneficial to the mangrove researchers. Although the initial cost of the drone data was found to be high (which also depends on the type of drone and sensors used), it becomes cost-effective upon monitoring areas of around 50km^2^ in size, or when using it for long-term monitoring of relatively small areas of several square kilometers. The higher spatial resolution, together with DEM, of the drone data delivered highly accurate classified maps compared to the Pleiades imagery. Among the classification algorithms tested, the efficiency of MAN for object-based classification, and MLI for pixel-based classification approaches was clear. The overlapped spectral signatures, especially for species like *R*. *apiculata*, *A*. *alba* and *C*. *equisetifolia* with similar tree heights, were responsible for the observed classification errors. Overall, the mangrove mapping based on drone aerial photos provided unprecedented results–especially in terms of image (object- and pixel-based) classification and accuracy, showing that drone technology could be used as an alternative to satellite-based monitoring/management of the mangrove ecosystems. While the drone’s image quality (spectral and radiometric resolutions) depends on the types of sensors used, the limitations of its data efficiency (coverage area, data acquisition/processing time and user-cost) depend on the model of drone used. Certainly, the development of drone technology towards longer battery run-time (enabling more area coverage), waterproof nature (enabling operation on rainy days), proximity sensors (enabling under-canopy monitoring), simplified data size (decreased processing time), hyperspectral sensor, and active remote sensing (e.g., Lidar) systems will make them even more useful in the future, especially for species-level discrimination in relatively low-diversity settings such as mangrove forests.

## Supporting information

S1 File Supplementary information. Detailed information on classification analysis, including iteration results, statistical analysis and error matrices.
(DOCX)

S2 File Protocol. Stepwise protocol of image analysis.
(DOCX)

S3 File Authorization letter from UMT for using Pleiades image. (PDF)

S4 File Authorization letter from UMT for data collection. (PDF)
