# Supplementary material for: The advantages of using drones over space-borne imagery in the mapping of mangrove forests
Source: PLoS One. 2018 Jul 18;13(7):e0200288. doi: 10.1371/journal.pone.0200288 (PMC6051606; doi:10.1371/journal.pone.0200288)

**Supplementary material**

**The advantages of drone over space-borne imagery in mapping mangrove forests**

Contents

[1. Accuracy Analysis & Statistical Test 2](#_Toc515350809)

[1.1. Overall Accuracy (OA) 2](#_Toc515350810)

[1.1.1. OA Iterations results, Mean, Median and Standard Deviation 2](#_Toc515350811)

[1.1.2. OA Kruskal-Wallis test 2](#_Toc515350812)

[1.1.3. OA Mann-Whitney Pairwise Comparison 2](#_Toc515350813)

[1.2. Kappa 3](#_Toc515350814)

[1.2.1. Kappa Iterations results, Mean, Median and Standard Deviation 3](#_Toc515350815)

[1.2.2. Kappa Kruskal-Wallis test 3](#_Toc515350816)

[1.2.3. Kappa Mann-Whitney Pairwise Comparison 3](#_Toc515350817)

[1.3. Specific Producer Accuracy (SPA) 4](#_Toc515350818)

[1.3.1. SPA Iterations results, Mean, Median and Standard Deviation 4](#_Toc515350819)

[1.3.2. SPA Kruskal-Wallis test 6](#_Toc515350820)

[1.3.3. SPA Mann-Whitney Pairwise Comparison 6](#_Toc515350821)

[1.4. Specific User Accuracy (SUA) 7](#_Toc515350822)

[1.4.1. SUA Iterations results, Mean, Median and Standard Deviation 7](#_Toc515350823)

[1.4.2. SUA Kruskal-Wallis test 9](#_Toc515350824)

[1.4.3. Mann-Whitney Pairwise Comparison 9](#_Toc515350825)

[2. Best maps and Error Matrix 10](#_Toc515350826)

[2.1. Pixel Based - Maximum Likelihood Algorithm 10](#_Toc515350827)

[2.1.1. Satellite Imagery (S6PMLI 3B) 10](#_Toc515350828)

[2.1.2. Drone Imagery – 6 classes (D6PMLI 2A) 11](#_Toc515350829)

[2.1.3. Drone Imagery – 10 Classes (D10PMLI 4A) 12](#_Toc515350830)

[2.2. Pixel based - Spectral Angle Mapping Algorithm 13](#_Toc515350831)

[2.2.1. Satellite Imagery (S6PSAM 4B) 13](#_Toc515350832)

[2.2.2. Drone Imagery – 6 Classes (D6PSAM 3B) 14](#_Toc515350833)

[2.2.3. Drone Imagery – 10 Classes (D10PSAM 3B) 15](#_Toc515350834)

[2.3. Object Based - Manual Classification 16](#_Toc515350835)

[2.3.1. Satellite Imagery (S6OMAN 5A) 16](#_Toc515350836)

[2.3.2. Drone Imagery – 6 classes (D6OMAN 2B) 17](#_Toc515350837)

[2.3.3. Drone Imagery – 10 Classes (D10OMAN 4B) 18](#_Toc515350838)

[2.4. Pixel Based - Automatic Algorithm 19](#_Toc515350839)

[2.4.1. Satellite Imagery (S6OAUT 1B) 19](#_Toc515350840)

[2.4.2. Drone Imagery – 6 Classes (D6OAUT 5A) 20](#_Toc515350841)

[2.4.3. Drone Imagery – 10 Classed (D10OAUT 5A) 21](#_Toc515350842)

[3. Separability of land-cover classes based on pixel-based classification 22](#_Toc515350843)

[4. Relative Intensity of RGB and IR cameras in various wavelengths 23](#_Toc515350844)

[5. Accuracy of drone imagery across various resolutions 23](#_Toc515350845)

# 1. Accuracy Analysis & Statistical Test

## 1.1. Overall Accuracy (OA)

### 1.1.1. OA Iterations results, Mean, Median and Standard Deviation

| **ROI number** | **D10O AUT** | **D10O MAN** | **D10P MLI** | **D10P SAM** | **D6O AUT** | **D6O MAN** | **D6P MLI** | **D6P SAM** | **S6O AUT** | **S6O MAN** | **S6P MLI** | **S6P SAM** |
| --- | --- | --- | --- | --- | --- | --- | --- | --- | --- | --- | --- | --- |
| **1A** | 58.21 | 69.22 | 77.58 | 52.15 | 81.13 | 94.01 | 88.29 | 71.30 | 51.68 | 71.87 | 85.78 | 75.78 |
| **1B** | 65.36 | 70.17 | 77.36 | 51.74 | 78.73 | 93.90 | 90.58 | 72.18 | 75.92 | 72.60 | 82.45 | 76.15 |
| **2A** | 66.87 | 69.25 | 77.68 | 53.23 | 84.83 | 93.10 | 93.04 | 73.20 | 73.43 | 72.93 | 80.47 | 67.00 |
| **2B** | 68.06 | 70.39 | 77.98 | 51.60 | 77.29 | 94.81 | 89.17 | 71.73 | 64.39 | 71.59 | 83.08 | 75.87 |
| **3A** | 63.57 | 71.29 | 76.17 | 48.54 | 72.63 | 93.84 | 87.13 | 69.10 | 71.23 | 73.70 | 82.41 | 72.41 |
| **3B** | 66.17 | 68.34 | 78.22 | 55.03 | 76.11 | 94.07 | 93.36 | 76.00 | 69.45 | 70.74 | 88.30 | 73.38 |
| **4A** | 60.51 | 67.80 | 79.37 | 50.59 | 78.14 | 93.61 | 90.93 | 72.97 | 71.41 | 73.88 | 85.85 | 73.20 |
| **4B** | 67.42 | 71.86 | 74.35 | 51.87 | 80.13 | 94.30 | 88.83 | 71.75 | 70.32 | 70.60 | 87.85 | 77.35 |
| **5A** | 68.72 | 69.16 | 78.83 | 53.05 | 85.87 | 94.52 | 89.64 | 73.90 | 74.83 | 77.81 | 78.28 | 59.81 |
| **5B** | 60.70 | 70.48 | 77.51 | 51.11 | 66.99 | 93.39 | 90.30 | 69.72 | 53.69 | 66.57 | 78.07 | 69.08 |
| **Mean** | **64.56** | **69.80** | **77.51** | **51.89** | **78.18** | **93.96** | **90.13** | **72.19** | **67.64** | **72.23** | **83.25** | **72.00** |
| **Median** | **65.77** | **69.71** | **77.63** | **51.80** | **78.43** | **93.96** | **89.97** | **71.97** | **70.77** | **72.23** | **82.76** | **73.29** |
| **Standard Deviation** | **3.45** | **1.21** | **1.33** | **1.63** | **5.27** | **0.49** | **1.87** | **1.89** | **8.07** | **2.72** | **3.47** | **5.11** |

### 1.1.2. OA Kruskal-Wallis test

H (chi2) : 103.4

Hc (tie corrected) : 103.4

p (same) : 3.728E-17

There is a significant difference between sample medians

### 1.1.3. OA Mann-Whitney Pairwise Comparison

| **OA** | **D10O MAN** | **D10O AUT** | **D10P MLI** | **D10P SAM** | **D6O MAN** | **D6O AUT** | **D6P MLI** | **D6P SAM** | **S6O MAN** | **S6O AUT** | **S6P MLI** | **S6P SAM** |
| --- | --- | --- | --- | --- | --- | --- | --- | --- | --- | --- | --- | --- |
| **D10OMAN** |  | 0.0004 | 0.0002 | 0.0002 | 0.0002 | 0.0028 | 0.0002 | 0.0125 | 0.0101 | 0.5706 | 0.0002 | 0.1040 |
| **D10OAUT** | 0.0004 |  | 0.0002 | 0.0002 | 0.0002 | 0.0004 | 0.0002 | 0.0002 | 0.0006 | 0.0757 | 0.0002 | 0.0046 |
| **D10PMLI** | 0.0002 | 0.0002 |  | 0.0002 | 0.0002 | 0.5708 | 0.0002 | 0.0002 | 0.0010 | 0.0003 | 0.0008 | 0.0009 |
| **D10PSAM** | 0.0002 | 0.0002 | 0.0002 |  | 0.0002 | 0.0002 | 0.0002 | 0.0002 | 0.0002 | 0.0011 | 0.0002 | 0.0002 |
| **D6OMAN** | 0.0002 | 0.0002 | 0.0002 | 0.0002 |  | 0.0002 | 0.0003 | 0.0002 | 0.0002 | 0.0002 | 0.0002 | 0.0002 |
| **D6OAUT** | 0.0028 | 0.0004 | 0.5708 | 0.0002 | 0.0002 |  | 0.0002 | 0.0073 | 0.0101 | 0.0028 | 0.0310 | 0.0140 |
| **D6PMLI** | 0.0002 | 0.0002 | 0.0002 | 0.0002 | 0.0003 | 0.0002 |  | 0.0002 | 0.0002 | 0.0002 | 0.0004 | 0.0002 |
| **D6PSAM** | 0.0125 | 0.0002 | 0.0002 | 0.0002 | 0.0002 | 0.0073 | 0.0002 |  | 1.0000 | 0.2413 | 0.0002 | 0.4723 |
| **S6OMAN** | 0.0101 | 0.0006 | 0.0010 | 0.0002 | 0.0002 | 0.0101 | 0.0002 | 1.0000 |  | 0.2123 | 0.0002 | 0.6232 |
| **S6OAUT** | 0.5706 | 0.0757 | 0.0003 | 0.0011 | 0.0002 | 0.0028 | 0.0002 | 0.2413 | 0.2123 |  | 0.0002 | 0.1855 |
| **S6PMLI** | 0.0002 | 0.0002 | 0.0008 | 0.0002 | 0.0002 | 0.0310 | 0.0004 | 0.0002 | 0.0002 | 0.0002 |  | 0.0002 |
| **S6PSAM** | 0.1040 | 0.0046 | 0.0009 | 0.0002 | 0.0002 | 0.0140 | 0.0002 | 0.4723 | 0.6232 | 0.1855 | 0.0002 |  |

Notes : Grey cells show insignificant difference (p > 0.05)

Values above and below diagonal are the same.

## 1.2. Kappa

### 1.2.1. Kappa Iterations results, Mean, Median and Standard Deviation

| ROI number | D10O AUT | D10O MAN | D10P MLI | D10P SAM | D6O AUT | D6O MAN | D6P MLI | D6P SAM | S6O AUT | S6O MAN | S6P MLI | S6P SAM |
| --- | --- | --- | --- | --- | --- | --- | --- | --- | --- | --- | --- | --- |
| 1A | 0.53 | 0.66 | 0.75 | 0.47 | 0.77 | 0.93 | 0.86 | 0.66 | 0.42 | 0.66 | 0.83 | 0.71 |
| 1B | 0.61 | 0.67 | 0.75 | 0.46 | 0.75 | 0.93 | 0.89 | 0.67 | 0.71 | 0.67 | 0.79 | 0.71 |
| 2A | 0.63 | 0.66 | 0.75 | 0.48 | 0.82 | 0.92 | 0.92 | 0.68 | 0.68 | 0.67 | 0.77 | 0.60 |
| 2B | 0.64 | 0.67 | 0.75 | 0.46 | 0.73 | 0.94 | 0.87 | 0.66 | 0.57 | 0.66 | 0.80 | 0.71 |
| 3A | 0.59 | 0.67 | 0.73 | 0.43 | 0.67 | 0.93 | 0.85 | 0.63 | 0.65 | 0.68 | 0.79 | 0.67 |
| 3B | 0.62 | 0.65 | 0.75 | 0.49 | 0.71 | 0.93 | 0.92 | 0.71 | 0.63 | 0.65 | 0.86 | 0.68 |
| 4A | 0.56 | 0.64 | 0.77 | 0.45 | 0.74 | 0.92 | 0.89 | 0.68 | 0.66 | 0.69 | 0.83 | 0.68 |
| 4B | 0.64 | 0.68 | 0.71 | 0.46 | 0.76 | 0.93 | 0.87 | 0.66 | 0.64 | 0.65 | 0.85 | 0.73 |
| 5A | 0.65 | 0.65 | 0.76 | 0.48 | 0.83 | 0.93 | 0.88 | 0.69 | 0.70 | 0.73 | 0.74 | 0.52 |
| 5B | 0.56 | 0.67 | 0.75 | 0.46 | 0.60 | 0.92 | 0.88 | 0.64 | 0.46 | 0.60 | 0.74 | 0.63 |
| Mean | 0.60 | 0.66 | 0.75 | 0.47 | 0.74 | 0.93 | 0.88 | 0.67 | 0.61 | 0.67 | 0.80 | 0.66 |
| Median | 0.62 | 0.66 | 0.75 | 0.46 | 0.74 | 0.93 | 0.88 | 0.66 | 0.65 | 0.67 | 0.79 | 0.68 |
| Standard Deviation | 0.04 | 0.01 | 0.01 | 0.02 | 0.06 | 0.01 | 0.02 | 0.02 | 0.09 | 0.03 | 0.04 | 0.06 |

### 1.2.2. Kappa Kruskal-Wallis test

H (chi2) : 101.1

Hc (tie corrected) : 101.3

p (same) : 9.829E-17

There is a significant difference between sample medians

### 1.2.3. Kappa Mann-Whitney Pairwise Comparison

| **Kappa** | **D10O MAN** | **D10O AUT** | **D10P MLI** | **D10P SAM** | **D6O MAN** | **D6O AUT** | **D6P MLI** | **D6P SAM** | **S6O MAN** | **S6O AUT** | **S6P MLI** | **S6P SAM** |
| --- | --- | --- | --- | --- | --- | --- | --- | --- | --- | --- | --- | --- |
| **D10OMAN** |  | 0.0004 | 0.0002 | 0.0002 | 0.0002 | 0.0046 | 0.0002 | 0.5205 | 0.4274 | 0.3847 | 0.0002 | 0.2730 |
| **D10OAUT** | 0.0004 |  | 0.0002 | 0.0002 | 0.0002 | 0.0010 | 0.0002 | 0.0010 | 0.0017 | 0.1620 | 0.0002 | 0.0258 |
| **D10PMLI** | 0.0002 | 0.0002 |  | 0.0002 | 0.0002 | 0.6500 | 0.0002 | 0.0002 | 0.0002 | 0.0002 | 0.0140 | 0.0003 |
| **D10PSAM** | 0.0002 | 0.0002 | 0.0002 |  | 0.0002 | 0.0002 | 0.0002 | 0.0002 | 0.0002 | 0.0140 | 0.0002 | 0.0002 |
| **D6OMAN** | 0.0002 | 0.0002 | 0.0002 | 0.0002 |  | 0.0002 | 0.0002 | 0.0002 | 0.0002 | 0.0002 | 0.0002 | 0.0002 |
| **D6OAUT** | 0.0046 | 0.0010 | 0.6500 | 0.0002 | 0.0002 |  | 0.0002 | 0.0073 | 0.0091 | 0.0028 | 0.0312 | 0.0155 |
| **D6PMLI** | 0.0002 | 0.0002 | 0.0002 | 0.0002 | 0.0002 | 0.0002 |  | 0.0002 | 0.0002 | 0.0002 | 0.0004 | 0.0002 |
| **D6PSAM** | 0.5205 | 0.0010 | 0.0002 | 0.0002 | 0.0002 | 0.0073 | 0.0002 |  | 0.9698 | 0.2413 | 0.0002 | 0.4727 |
| **S6OMAN** | 0.4274 | 0.0017 | 0.0002 | 0.0002 | 0.0002 | 0.0091 | 0.0002 | 0.9698 |  | 0.2123 | 0.0002 | 0.6232 |
| **S6OAUT** | 0.3847 | 0.1620 | 0.0002 | 0.0140 | 0.0002 | 0.0028 | 0.0002 | 0.2413 | 0.2123 |  | 0.0002 | 0.2123 |
| **S6PMLI** | 0.0002 | 0.0002 | 0.0140 | 0.0002 | 0.0002 | 0.0312 | 0.0004 | 0.0002 | 0.0002 | 0.0002 |  | 0.0002 |
| **S6PSAM** | 0.2730 | 0.0258 | 0.0003 | 0.0002 | 0.0002 | 0.0155 | 0.0002 | 0.4727 | 0.6232 | 0.2123 | 0.0002 |  |

Notes : Grey cells show insignificant difference (p > 0.05)

Values above and below diagonal are the same.

## 1.3. Specific Producer Accuracy (SPA)

### 1.3.1. SPA Iterations results, Mean, Median and Standard Deviation

| Land cover | ROI number | D10O AUT | D10O MAN | D10P MLI | D10P SAM | D6O AUT | D6O MAN | D6P MLI | D6P SAM | S6O AUT | S6O MAN | S6P MLI | S6P SAM |
| --- | --- | --- | --- | --- | --- | --- | --- | --- | --- | --- | --- | --- | --- |
| *C. equisetifolia* | 1A | 75.12 | 68.10 | 88.12 | 31.66 | 80.48 | 96.54 | 85.50 | 66.11 | 50.88 | 41.76 | 83.57 | 65.46 |
|  | 1B | 65.22 | 72.05 | 72.56 | 30.04 | 78.45 | 92.49 | 95.60 | 73.90 | 85.71 | 53.30 | 82.45 | 71.31 |
|  | 2A | 60.06 | 70.32 | 81.64 | 37.73 | 84.65 | 90.88 | 95.22 | 76.78 | 71.15 | 60.66 | 58.10 | 41.90 |
|  | 2B | 87.73 | 69.80 | 86.76 | 39.79 | 87.15 | 97.96 | 83.82 | 65.30 | 91.87 | 34.36 | 70.00 | 78.61 |
|  | 3A | 47.48 | 76.75 | 84.69 | 28.56 | 82.36 | 95.27 | 84.82 | 74.02 | 85.40 | 57.78 | 66.95 | 50.42 |
|  | 3B | 61.34 | 61.51 | 83.25 | 37.89 | 81.16 | 93.67 | 93.68 | 78.04 | 85.78 | 37.47 | 77.01 | 80.61 |
|  | 4A | 62.86 | 67.42 | 85.66 | 29.56 | 73.56 | 93.76 | 90.23 | 72.50 | 81.09 | 43.65 | 76.11 | 66.67 |
|  | 4B | 45.73 | 72.63 | 75.90 | 26.45 | 59.35 | 95.15 | 89.78 | 73.27 | 74.61 | 51.30 | 77.37 | 67.88 |
|  | 5A | 54.35 | 61.72 | 85.35 | 34.24 | 88.09 | 96.67 | 91.61 | 78.03 | 55.23 | 72.85 | 42.38 | 44.80 |
|  | 5B | 68.82 | 78.39 | 75.96 | 29.90 | 69.78 | 92.26 | 82.97 | 61.99 | 70.14 | 39.39 | 67.78 | 98.89 |
| *A. alba* | 1A | 63.83 | 49.92 | 82.24 | 41.02 | 96.05 | 76.71 | 66.84 | 63.34 | 18.55 | 98.45 | 59.89 | 87.36 |
|  | 1B | 82.82 | 36.87 | 65.03 | 44.63 | 89.90 | 75.54 | 83.46 | 65.34 | 98.45 | 92.99 | 95.83 | 88.06 |
|  | 2A | 77.84 | 34.55 | 74.13 | 38.40 | 95.20 | 76.53 | 89.30 | 63.91 | 85.32 | 93.65 | 92.86 | 92.86 |
|  | 2B | 82.39 | 52.47 | 65.67 | 39.04 | 93.16 | 75.74 | 83.44 | 69.01 | 46.17 | 97.94 | 85.56 | 79.44 |
|  | 3A | 73.14 | 43.21 | 65.04 | 32.80 | 96.10 | 73.89 | 87.58 | 53.28 | 75.40 | 96.44 | 89.94 | 94.69 |
|  | 3B | 69.13 | 43.76 | 75.75 | 44.57 | 95.98 | 78.39 | 85.57 | 69.86 | 69.56 | 95.03 | 92.08 | 84.97 |
|  | 4A | 64.14 | 40.88 | 76.83 | 38.83 | 98.97 | 77.23 | 83.38 | 64.08 | 79.19 | 98.00 | 84.75 | 82.77 |
|  | 4B | 73.19 | 45.92 | 66.54 | 42.45 | 93.25 | 75.02 | 83.53 | 59.72 | 80.49 | 93.44 | 88.38 | 91.62 |
|  | 5A | 50.15 | 38.95 | 71.28 | 42.52 | 95.19 | 78.18 | 84.68 | 64.21 | 98.31 | 95.91 | 89.62 | 62.63 |
|  | 5B | 77.76 | 47.87 | 69.26 | 47.24 | 94.06 | 74.13 | 82.06 | 59.19 | 24.16 | 95.49 | 72.64 | 79.54 |
| *N. fruticans* | 1A | 65.22 | 95.71 | 66.11 | 24.71 | 59.49 | 97.66 | 86.41 | 23.62 | 1.10 | 50.90 | 76.67 | 50.83 |
|  | 1B | 62.36 | 99.63 | 69.98 | 15.12 | 73.52 | 97.65 | 87.16 | 23.04 | 17.65 | 28.29 | 58.06 | 60.00 |
|  | 2A | 79.71 | 98.01 | 73.39 | 20.75 | 75.26 | 97.35 | 86.21 | 23.01 | 38.96 | 47.25 | 45.00 | 11.67 |
|  | 2B | 48.44 | 97.29 | 63.46 | 17.83 | 61.87 | 97.96 | 88.97 | 24.66 | 0.00 | 32.03 | 69.72 | 57.22 |
|  | 3A | 65.24 | 100.00 | 61.00 | 17.01 | 64.24 | 95.62 | 87.31 | 22.21 | 13.26 | 35.26 | 74.72 | 56.67 |
|  | 3B | 60.36 | 96.13 | 63.98 | 17.67 | 76.25 | 99.64 | 87.41 | 25.81 | 16.67 | 43.95 | 62.78 | 58.06 |
|  | 4A | 52.64 | 95.67 | 68.20 | 22.26 | 70.10 | 97.98 | 83.85 | 22.19 | 13.05 | 39.01 | 67.78 | 67.78 |
|  | 4B | 82.59 | 99.64 | 64.57 | 17.41 | 82.67 | 97.33 | 87.40 | 22.08 | 23.54 | 39.82 | 67.78 | 50.00 |
|  | 5A | 87.58 | 99.64 | 72.79 | 21.82 | 77.22 | 97.62 | 85.68 | 19.43 | 57.73 | 39.02 | 80.56 | 59.57 |
|  | 5B | 57.41 | 95.68 | 61.85 | 20.76 | 65.19 | 97.69 | 89.99 | 24.31 | 4.90 | 39.95 | 72.47 | 32.58 |
| *R. apiculata* | 1A | 31.25 | 98.24 | 87.38 | 70.54 | 64.55 | 97.96 | 93.59 | 78.89 | 38.69 | 51.92 | 94.44 | 54.17 |
|  | 1B | 23.60 | 98.67 | 71.44 | 75.41 | 46.20 | 99.98 | 85.50 | 76.49 | 53.85 | 61.54 | 98.61 | 49.72 |
|  | 2A | 32.02 | 98.96 | 81.71 | 75.00 | 55.18 | 98.62 | 90.25 | 77.11 | 46.49 | 45.76 | 94.44 | 55.28 |
|  | 2B | 25.39 | 97.97 | 86.19 | 75.63 | 50.15 | 99.28 | 89.55 | 79.26 | 46.65 | 67.32 | 93.06 | 61.11 |
|  | 3A | 34.23 | 98.26 | 92.67 | 79.30 | 48.13 | 99.16 | 79.09 | 74.33 | 53.26 | 51.95 | 92.50 | 56.39 |
|  | 3B | 21.32 | 98.94 | 75.61 | 77.37 | 54.72 | 98.73 | 94.68 | 83.08 | 44.14 | 60.84 | 97.50 | 15.56 |
|  | 4A | 29.55 | 98.73 | 85.32 | 74.90 | 65.32 | 98.64 | 91.66 | 80.27 | 56.62 | 65.84 | 97.22 | 43.33 |
|  | 4B | 29.99 | 98.19 | 86.23 | 75.21 | 51.54 | 99.25 | 89.56 | 84.15 | 41.63 | 47.86 | 92.78 | 53.61 |
|  | 5A | 47.05 | 97.38 | 83.36 | 79.56 | 56.78 | 98.65 | 82.38 | 86.59 | 56.00 | 64.57 | 80.28 | 11.11 |
|  | 5B | 8.05 | 99.58 | 85.13 | 68.70 | 36.26 | 99.25 | 91.90 | 78.03 | 39.13 | 48.44 | 91.67 | 42.22 |
| Dead tree | 1A | 86.00 | 61.27 | 89.37 | 69.76 |  |  |  |  |  |  |  |  |
|  | 1B | 96.51 | 64.79 | 91.02 | 50.60 |  |  |  |  |  |  |  |  |
|  | 2A | 92.84 | 62.34 | 91.71 | 63.17 |  |  |  |  |  |  |  |  |
|  | 2B | 89.51 | 63.57 | 87.96 | 48.84 |  |  |  |  |  |  |  |  |
|  | 3A | 83.04 | 64.77 | 82.52 | 56.19 |  |  |  |  |  |  |  |  |
|  | 3B | 97.98 | 61.82 | 96.77 | 66.84 |  |  |  |  |  |  |  |  |
|  | 4A | 88.56 | 64.90 | 81.64 | 43.28 |  |  |  |  |  |  |  |  |
|  | 4B | 94.43 | 60.85 | 92.74 | 64.66 |  |  |  |  |  |  |  |  |
|  | 5A | 70.74 | 67.30 | 86.56 | 49.92 |  |  |  |  |  |  |  |  |
|  | 5B | 98.95 | 58.97 | 95.71 | 61.55 |  |  |  |  |  |  |  |  |
| *B. cylindrica* | 1A | 54.57 | 25.89 | 49.99 | 28.32 |  |  |  |  |  |  |  |  |
|  | 1B | 18.81 | 25.13 | 69.69 | 35.29 |  |  |  |  |  |  |  |  |
|  | 2A | 31.52 | 25.88 | 57.89 | 35.30 |  |  |  |  |  |  |  |  |
|  | 2B | 33.18 | 25.16 | 68.77 | 29.36 |  |  |  |  |  |  |  |  |
|  | 3A | 28.08 | 25.52 | 69.65 | 22.76 |  |  |  |  |  |  |  |  |
|  | 3B | 52.04 | 25.52 | 57.04 | 32.34 |  |  |  |  |  |  |  |  |
|  | 4A | 37.32 | 13.79 | 53.48 | 25.60 |  |  |  |  |  |  |  |  |
|  | 4B | 31.33 | 37.46 | 47.96 | 39.51 |  |  |  |  |  |  |  |  |
|  | 5A | 59.67 | 26.82 | 61.35 | 33.31 |  |  |  |  |  |  |  |  |
|  | 5B | 26.87 | 24.16 | 67.63 | 31.39 |  |  |  |  |  |  |  |  |
| *L. racemosa* | 1A | 53.78 | 24.97 | 51.45 | 16.70 |  |  |  |  |  |  |  |  |
|  | 1B | 52.73 | 40.32 | 61.10 | 23.13 |  |  |  |  |  |  |  |  |
|  | 2A | 58.06 | 34.21 | 61.66 | 29.03 |  |  |  |  |  |  |  |  |
|  | 2B | 62.21 | 32.86 | 48.50 | 13.78 |  |  |  |  |  |  |  |  |
|  | 3A | 38.94 | 27.55 | 54.62 | 22.44 |  |  |  |  |  |  |  |  |
|  | 3B | 70.54 | 37.44 | 61.11 | 12.39 |  |  |  |  |  |  |  |  |
|  | 4A | 67.37 | 34.07 | 69.67 | 21.98 |  |  |  |  |  |  |  |  |
|  | 4B | 54.10 | 32.95 | 54.10 | 16.99 |  |  |  |  |  |  |  |  |
|  | 5A | 50.95 | 32.30 | 51.47 | 23.98 |  |  |  |  |  |  |  |  |
|  | 5B | 72.29 | 34.59 | 64.86 | 12.99 |  |  |  |  |  |  |  |  |
| *S. alba* | 1A | 60.16 | 58.51 | 74.75 | 35.04 |  |  |  |  |  |  |  |  |
|  | 1B | 70.86 | 58.79 | 83.36 | 37.54 |  |  |  |  |  |  |  |  |
|  | 2A | 62.22 | 54.95 | 76.54 | 37.59 |  |  |  |  |  |  |  |  |
|  | 2B | 69.41 | 63.20 | 80.85 | 46.96 |  |  |  |  |  |  |  |  |
|  | 3A | 76.99 | 53.79 | 82.80 | 39.64 |  |  |  |  |  |  |  |  |
|  | 3B | 62.01 | 64.47 | 83.02 | 39.99 |  |  |  |  |  |  |  |  |
|  | 4A | 51.48 | 60.93 | 72.45 | 47.70 |  |  |  |  |  |  |  |  |
|  | 4B | 78.94 | 58.17 | 87.96 | 39.41 |  |  |  |  |  |  |  |  |
|  | 5A | 70.83 | 62.65 | 82.94 | 32.61 |  |  |  |  |  |  |  |  |
|  | 5B | 62.37 | 56.03 | 87.46 | 48.48 |  |  |  |  |  |  |  |  |
| Mean | | 60.07 | 61.30 | 74.03 | 39.63 | 74.19 | 91.80 | 87.04 | 59.51 | 52.27 | 60.29 | 79.03 | 61.43 |
| Median | | 62.22 | 61.39 | 74.44 | 37.57 | 75.76 | 97.00 | 87.24 | 65.73 | 53.55 | 51.94 | 80.42 | 59.78 |
| Standard Deviation | | 20.91 | 25.76 | 12.34 | 18.44 | 16.61 | 9.30 | 5.09 | 22.23 | 28.01 | 22.69 | 14.28 | 21.43 |

### 1.3.2. SPA Kruskal-Wallis test

H (chi2) : 223.7

Hc (tie corrected) : 223.7

p (same) : 8.951E-42

There is a significant difference between sample medians

### 1.3.3. SPA Mann-Whitney Pairwise Comparison

| SPA | **D10O MAN** | **D10O AUT** | **D10P MLI** | **D10P SAM** | **D6O MAN** | **D6O AUT** | **D6P MLI** | **D6P SAM** | **S6O MAN** | **S6O AUT** | **S6P MLI** | **S6P SAM** |
| --- | --- | --- | --- | --- | --- | --- | --- | --- | --- | --- | --- | --- |
| D10OMAN |  | 0.9090 | 0.0005 | 0.0000 | 0.0000 | 0.0133 | 0.0000 | 0.9312 | 0.5950 | 0.1265 | 0.0007 | 0.9756 |
| D10OAUT | 0.9090 |  | 0.0000 | 0.0000 | 0.0000 | 0.0008 | 0.0000 | 0.6702 | 0.6500 | 0.1582 | 0.0000 | 0.8564 |
| D10PMLI | 0.0005 | 0.0000 |  | 0.0000 | 0.0000 | 0.7743 | 0.0000 | 0.0016 | 0.0003 | 0.0000 | 0.0273 | 0.0008 |
| D10PSAM | 0.0000 | 0.0000 | 0.0000 |  | 0.0000 | 0.0000 | 0.0000 | 0.0000 | 0.0000 | 0.0071 | 0.0000 | 0.0000 |
| D6OMAN | 0.0000 | 0.0000 | 0.0000 | 0.0000 |  | 0.0000 | 0.0002 | 0.0000 | 0.0000 | 0.0000 | 0.0000 | 0.0000 |
| D6OAUT | 0.0133 | 0.0008 | 0.7743 | 0.0000 | 0.0000 |  | 0.0006 | 0.0080 | 0.0036 | 0.0004 | 0.2462 | 0.0080 |
| D6PMLI | 0.0000 | 0.0000 | 0.0000 | 0.0000 | 0.0002 | 0.0006 |  | 0.0000 | 0.0001 | 0.0000 | 0.0355 | 0.0000 |
| D6PSAM | 0.9312 | 0.6702 | 0.0016 | 0.0000 | 0.0000 | 0.0080 | 0.0000 |  | 0.6615 | 0.2385 | 0.0001 | 0.8814 |
| S6OMAN | 0.5950 | 0.6500 | 0.0003 | 0.0000 | 0.0000 | 0.0036 | 0.0001 | 0.6615 |  | 0.3169 | 0.0004 | 0.4273 |
| S6OAUT | 0.1265 | 0.1582 | 0.0000 | 0.0071 | 0.0000 | 0.0004 | 0.0000 | 0.2385 | 0.3169 |  | 0.0000 | 0.1397 |
| S6PMLI | 0.0007 | 0.0000 | 0.0273 | 0.0000 | 0.0000 | 0.2462 | 0.0355 | 0.0001 | 0.0004 | 0.0000 |  | 0.0001 |
| S6PSAM | 0.9756 | 0.8564 | 0.0008 | 0.0000 | 0.0000 | 0.0080 | 0.0000 | 0.8814 | 0.4273 | 0.1397 | 0.0001 |  |

Notes : Grey cells show insignificant difference (p > 0.05)

Values above and below diagonal are the same.

## 1.4. Specific User Accuracy (SUA)

### 1.4.1. SUA Iterations results, Mean, Median and Standard Deviation

| Land cover | ROI number | D10O AUT | D10O MAN | D10P MLI | D10P SAM | D6O AUT | D6O MAN | D6P MLI | D6P SAM | S6O AUT | S6O MAN | S6P MLI | S6P SAM |
| --- | --- | --- | --- | --- | --- | --- | --- | --- | --- | --- | --- | --- | --- |
| *C. equisetifolia* | 1A | 54.67 | 66.08 | 73.23 | 54.08 | 70.73 | 99.73 | 87.02 | 59.98 | 22.56 | 100.00 | 63.42 | 68.71 |
|  | 1B | 63.26 | 62.98 | 81.63 | 45.96 | 62.40 | 99.65 | 84.29 | 58.84 | 53.65 | 100.00 | 52.39 | 66.15 |
|  | 2A | 80.83 | 67.63 | 77.82 | 48.17 | 79.03 | 99.72 | 89.73 | 64.74 | 51.03 | 100.00 | 55.47 | 66.08 |
|  | 2B | 60.82 | 61.51 | 72.42 | 51.05 | 67.42 | 99.67 | 85.45 | 60.65 | 39.73 | 100.00 | 53.85 | 70.57 |
|  | 3A | 39.47 | 72.26 | 63.30 | 42.74 | 48.23 | 99.97 | 84.57 | 53.14 | 45.32 | 100.00 | 56.90 | 58.63 |
|  | 3B | 78.99 | 54.97 | 81.37 | 60.47 | 51.86 | 99.41 | 89.38 | 65.54 | 43.81 | 100.00 | 72.40 | 77.19 |
|  | 4A | 53.38 | 62.89 | 79.69 | 42.61 | 71.47 | 99.64 | 87.41 | 57.99 | 44.30 | 100.00 | 66.02 | 68.57 |
|  | 4B | 79.27 | 65.94 | 70.93 | 50.87 | 76.71 | 99.74 | 85.32 | 61.73 | 48.62 | 100.00 | 72.70 | 65.15 |
|  | 5A | 67.32 | 65.99 | 70.44 | 53.85 | 78.96 | 99.66 | 88.94 | 63.53 | 82.07 | 100.00 | 81.72 | 73.48 |
|  | 5B | 66.80 | 63.30 | 79.34 | 57.92 | 67.74 | 99.72 | 88.20 | 53.02 | 15.11 | 100.00 | 26.46 | 60.14 |
| *A. alba* | 1A | 55.43 | 60.97 | 64.92 | 55.36 | 66.66 | 96.90 | 88.16 | 85.21 | 90.11 | 51.81 | 97.76 | 63.86 |
|  | 1B | 45.34 | 59.55 | 64.38 | 59.46 | 58.81 | 99.87 | 83.16 | 77.00 | 92.31 | 49.05 | 88.24 | 57.95 |
|  | 2A | 46.79 | 54.98 | 65.35 | 50.81 | 63.51 | 98.16 | 86.54 | 83.67 | 78.81 | 49.03 | 78.60 | 57.88 |
|  | 2B | 48.28 | 64.60 | 67.48 | 57.07 | 62.24 | 98.52 | 89.46 | 84.19 | 95.91 | 52.14 | 92.77 | 72.77 |
|  | 3A | 41.79 | 69.54 | 70.42 | 46.04 | 62.99 | 98.29 | 82.37 | 73.96 | 100.00 | 48.17 | 84.07 | 68.21 |
|  | 3B | 53.30 | 50.60 | 70.04 | 59.31 | 62.33 | 98.38 | 89.40 | 88.25 | 97.21 | 53.02 | 89.39 | 49.44 |
|  | 4A | 46.63 | 51.47 | 63.82 | 63.52 | 69.23 | 97.74 | 84.09 | 80.72 | 100.00 | 52.06 | 95.24 | 56.67 |
|  | 4B | 41.86 | 71.03 | 58.56 | 55.67 | 58.58 | 98.96 | 89.16 | 90.18 | 85.21 | 48.82 | 83.42 | 63.13 |
|  | 5A | 49.48 | 55.57 | 70.67 | 54.44 | 66.97 | 97.71 | 77.35 | 87.44 | 61.88 | 55.48 | 70.96 | 47.76 |
|  | 5B | 37.82 | 64.89 | 64.32 | 52.28 | 53.10 | 98.99 | 88.71 | 77.19 | 100.00 | 44.31 | 99.06 | 75.05 |
| *N. fruticans* | 1A | 74.10 | 83.24 | 80.45 | 30.72 | 82.99 | 100.00 | 81.17 | 37.61 | 1.58 | 75.50 | 75.00 | 60.80 |
|  | 1B | 65.59 | 90.37 | 72.36 | 29.98 | 82.32 | 100.00 | 84.85 | 44.59 | 27.96 | 79.14 | 90.87 | 67.08 |
|  | 2A | 57.66 | 86.31 | 73.04 | 36.58 | 92.32 | 100.00 | 91.79 | 43.22 | 44.23 | 72.28 | 66.12 | 21.43 |
|  | 2B | 77.98 | 87.08 | 76.20 | 26.72 | 82.64 | 100.00 | 72.82 | 38.42 | 0.00 | 84.09 | 70.31 | 66.88 |
|  | 3A | 56.91 | 81.78 | 73.40 | 28.96 | 76.30 | 100.00 | 70.16 | 44.56 | 21.19 | 85.94 | 69.87 | 58.96 |
|  | 3B | 56.08 | 90.35 | 78.77 | 27.50 | 93.69 | 100.00 | 93.35 | 50.61 | 23.01 | 70.26 | 86.26 | 57.73 |
|  | 4A | 73.29 | 86.38 | 75.69 | 29.66 | 56.41 | 100.00 | 87.10 | 47.14 | 21.07 | 82.08 | 70.72 | 69.12 |
|  | 4B | 58.00 | 86.99 | 78.14 | 31.88 | 75.95 | 100.00 | 73.75 | 41.58 | 31.63 | 72.29 | 76.73 | 56.96 |
|  | 5A | 63.26 | 83.24 | 83.45 | 35.13 | 91.11 | 100.00 | 87.56 | 47.55 | 53.98 | 68.15 | 45.47 | 32.60 |
|  | 5B | 73.92 | 90.57 | 75.09 | 29.52 | 45.23 | 100.00 | 76.76 | 39.31 | 6.74 | 91.72 | 77.57 | 58.90 |
| *R. apiculata* | 1A | 98.16 | 48.45 | 80.09 | 46.82 | 95.39 | 79.44 | 77.38 | 58.44 | 100.00 | 46.82 | 89.95 | 70.91 |
|  | 1B | 97.73 | 50.88 | 92.11 | 44.20 | 99.90 | 76.45 | 93.40 | 62.15 | 100.00 | 49.36 | 94.16 | 99.44 |
|  | 2A | 99.14 | 46.77 | 87.58 | 51.93 | 96.57 | 76.37 | 91.11 | 59.22 | 100.00 | 50.12 | 81.15 | 49.75 |
|  | 2B | 95.72 | 52.99 | 80.29 | 43.43 | 98.60 | 79.58 | 93.32 | 60.31 | 100.00 | 46.66 | 97.67 | 67.07 |
|  | 3A | 94.38 | 59.33 | 82.55 | 30.48 | 97.53 | 77.37 | 93.77 | 60.92 | 100.00 | 50.22 | 96.80 | 76.89 |
|  | 3B | 99.01 | 35.84 | 81.48 | 56.75 | 97.76 | 78.52 | 89.21 | 63.54 | 100.00 | 46.61 | 82.98 | 77.78 |
|  | 4A | 95.07 | 49.16 | 83.86 | 49.67 | 99.65 | 77.77 | 87.77 | 63.21 | 100.00 | 52.81 | 88.83 | 77.23 |
|  | 4B | 98.34 | 50.20 | 79.73 | 42.41 | 95.60 | 78.09 | 90.27 | 58.20 | 100.00 | 43.16 | 93.56 | 99.48 |
|  | 5A | 97.89 | 49.26 | 81.20 | 46.74 | 97.05 | 80.73 | 86.56 | 58.68 | 100.00 | 75.38 | 99.66 | 19.14 |
|  | 5B | 97.25 | 50.09 | 88.84 | 46.25 | 94.38 | 75.30 | 91.28 | 61.39 | 100.00 | 32.25 | 89.67 | 38.58 |
| Dead tree | 1A | 71.52 | 75.69 | 76.36 | 54.87 |  |  |  |  |  |  |  |  |
|  | 1B | 73.44 | 69.51 | 88.00 | 54.69 |  |  |  |  |  |  |  |  |
|  | 2A | 75.16 | 76.39 | 71.77 | 51.10 |  |  |  |  |  |  |  |  |
|  | 2B | 80.20 | 69.01 | 87.52 | 64.32 |  |  |  |  |  |  |  |  |
|  | 3A | 87.49 | 66.85 | 84.66 | 69.41 |  |  |  |  |  |  |  |  |
|  | 3B | 59.79 | 76.76 | 74.85 | 53.74 |  |  |  |  |  |  |  |  |
|  | 4A | 73.20 | 75.13 | 87.19 | 49.45 |  |  |  |  |  |  |  |  |
|  | 4B | 74.32 | 69.74 | 75.80 | 56.38 |  |  |  |  |  |  |  |  |
|  | 5A | 64.65 | 77.81 | 88.44 | 51.83 |  |  |  |  |  |  |  |  |
|  | 5B | 75.23 | 67.73 | 71.56 | 52.74 |  |  |  |  |  |  |  |  |
| *B. cylindrica* | 1A | 37.81 | 54.50 | 72.98 | 43.61 |  |  |  |  |  |  |  |  |
|  | 1B | 23.13 | 38.11 | 63.19 | 38.15 |  |  |  |  |  |  |  |  |
|  | 2A | 34.77 | 41.02 | 67.13 | 44.66 |  |  |  |  |  |  |  |  |
|  | 2B | 36.41 | 50.21 | 66.86 | 34.39 |  |  |  |  |  |  |  |  |
|  | 3A | 36.60 | 39.36 | 77.34 | 38.09 |  |  |  |  |  |  |  |  |
|  | 3B | 35.95 | 52.37 | 55.56 | 34.61 |  |  |  |  |  |  |  |  |
|  | 4A | 32.95 | 30.52 | 75.85 | 40.14 |  |  |  |  |  |  |  |  |
|  | 4B | 35.41 | 54.95 | 56.20 | 39.01 |  |  |  |  |  |  |  |  |
|  | 5A | 43.85 | 40.65 | 61.15 | 50.46 |  |  |  |  |  |  |  |  |
|  | 5B | 35.66 | 51.65 | 70.77 | 31.92 |  |  |  |  |  |  |  |  |
| *L. racemosa* | 1A | 39.27 | 45.11 | 64.97 | 29.50 |  |  |  |  |  |  |  |  |
|  | 1B | 62.87 | 66.60 | 53.98 | 29.90 |  |  |  |  |  |  |  |  |
|  | 2A | 55.94 | 58.47 | 65.34 | 41.61 |  |  |  |  |  |  |  |  |
|  | 2B | 60.87 | 55.15 | 62.47 | 28.95 |  |  |  |  |  |  |  |  |
|  | 3A | 58.98 | 50.21 | 55.11 | 33.23 |  |  |  |  |  |  |  |  |
|  | 3B | 59.09 | 60.55 | 67.34 | 23.68 |  |  |  |  |  |  |  |  |
|  | 4A | 38.89 | 56.72 | 63.23 | 27.48 |  |  |  |  |  |  |  |  |
|  | 4B | 75.97 | 56.77 | 61.89 | 37.96 |  |  |  |  |  |  |  |  |
|  | 5A | 73.02 | 50.40 | 68.24 | 40.14 |  |  |  |  |  |  |  |  |
|  | 5B | 40.58 | 63.33 | 60.24 | 28.46 |  |  |  |  |  |  |  |  |
| *S. alba* | 1A | 82.35 | 58.45 | 49.06 | 8.66 |  |  |  |  |  |  |  |  |
|  | 1B | 50.74 | 89.99 | 50.25 | 9.90 |  |  |  |  |  |  |  |  |
|  | 2A | 74.29 | 88.06 | 67.47 | 11.03 |  |  |  |  |  |  |  |  |
|  | 2B | 65.82 | 72.76 | 47.06 | 9.83 |  |  |  |  |  |  |  |  |
|  | 3A | 43.84 | 86.90 | 47.70 | 9.53 |  |  |  |  |  |  |  |  |
|  | 3B | 85.07 | 73.16 | 65.28 | 10.71 |  |  |  |  |  |  |  |  |
|  | 4A | 82.75 | 69.79 | 52.42 | 9.72 |  |  |  |  |  |  |  |  |
|  | 4B | 60.90 | 89.82 | 54.31 | 11.13 |  |  |  |  |  |  |  |  |
|  | 5A | 49.31 | 100.00 | 49.40 | 7.76 |  |  |  |  |  |  |  |  |
|  | 5B | 82.78 | 61.45 | 59.61 | 12.99 |  |  |  |  |  |  |  |  |
| Mean | | 63.40 | 64.22 | 70.66 | 40.03 | 76.26 | 94.00 | 86.05 | 61.69 | 64.48 | 69.47 | 78.10 | 62.85 |
| Median | | 61.88 | 63.14 | 71.25 | 42.68 | 76.13 | 99.53 | 87.49 | 60.48 | 70.35 | 69.21 | 81.43 | 65.61 |
| Standard Deviation | | 19.70 | 15.16 | 10.86 | 15.35 | 16.28 | 9.32 | 5.70 | 14.53 | 34.37 | 22.04 | 16.39 | 15.91 |

### 1.4.2. SUA Kruskal-Wallis test

H (chi2) : 243

Hc (tie corrected) : 243

p (same) : 8.183E-46

There is a significant difference between sample medians

### 1.4.3. Mann-Whitney Pairwise Comparison

| SUA | **D10O MAN** | **D10O AUT** | **D10P MLI** | **D10P SAM** | **D6O MAN** | **D6O AUT** | **D6P MLI** | **D6P SAM** | **S6O MAN** | **S6O AUT** | **S6P MLI** | **S6P SAM** |
| --- | --- | --- | --- | --- | --- | --- | --- | --- | --- | --- | --- | --- |
| D10OMAN |  | 0.6935 | 0.0021 | 0.0000 | 0.0000 | 0.0003 | 0.0000 | 0.3627 | 0.4916 | 0.6398 | 0.0000 | 0.8959 |
| D10OAUT | 0.6935 |  | 0.0062 | 0.0000 | 0.0000 | 0.0012 | 0.0000 | 0.8043 | 0.1597 | 0.4145 | 0.0002 | 0.8915 |
| D10PMLI | 0.0021 | 0.0062 |  | 0.0000 | 0.0000 | 0.1023 | 0.0000 | 0.0004 | 0.4340 | 0.9756 | 0.0016 | 0.0030 |
| D10PSAM | 0.0000 | 0.0000 | 0.0000 |  | 0.0000 | 0.0000 | 0.0000 | 0.0000 | 0.0000 | 0.0007 | 0.0000 | 0.0000 |
| D6OMAN | 0.0000 | 0.0000 | 0.0000 | 0.0000 |  | 0.0000 | 0.0000 | 0.0000 | 0.0001 | 0.0071 | 0.0000 | 0.0000 |
| D6OAUT | 0.0003 | 0.0012 | 0.1023 | 0.0000 | 0.0000 |  | 0.0186 | 0.0001 | 0.2231 | 0.5403 | 0.5476 | 0.0024 |
| D6PMLI | 0.0000 | 0.0000 | 0.0000 | 0.0000 | 0.0000 | 0.0186 |  | 0.0000 | 0.0022 | 0.3090 | 0.0626 | 0.0000 |
| D6PSAM | 0.3627 | 0.8043 | 0.0004 | 0.0000 | 0.0000 | 0.0001 | 0.0000 |  | 0.2618 | 0.4725 | 0.0000 | 0.3891 |
| S6OMAN | 0.4916 | 0.1597 | 0.4340 | 0.0000 | 0.0001 | 0.2231 | 0.0022 | 0.2618 |  | 0.5492 | 0.1153 | 0.4612 |
| S6OAUT | 0.6398 | 0.4145 | 0.9756 | 0.0007 | 0.0071 | 0.5403 | 0.3090 | 0.4725 | 0.5492 |  | 0.5661 | 0.5727 |
| S6PMLI | 0.0000 | 0.0002 | 0.0016 | 0.0000 | 0.0000 | 0.5476 | 0.0626 | 0.0000 | 0.1153 | 0.5661 |  | 0.0001 |
| S6PSAM | 0.8959 | 0.8915 | 0.0030 | 0.0000 | 0.0000 | 0.0024 | 0.0000 | 0.3891 | 0.4612 | 0.5727 | 0.0001 |  |

Notes : Grey cells show insignificant difference (p > 0.05)

Values above and below diagonal are the same.

# 2. Best maps and Error Matrix

## 2.1. Pixel Based - Maximum Likelihood Algorithm

### 2.1.1. Satellite Imagery (S6PMLI 3B)

Image Source : Pleaides Satellite

Classification Method : Pixel Based – Maximum Likelihood

Number of land covers class : 6

Training sites : 3B

Validated by : 3A


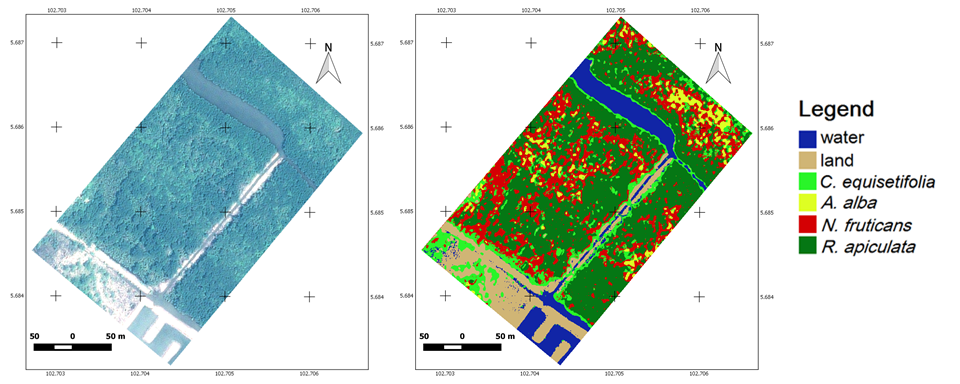


| S6PMLI 3B | Water | Land | *C. equise-tiofolia* | *A. alba* | *N. fruticans* | *R. apiculata* | Total | User Accuracy | Commission Error |
| --- | --- | --- | --- | --- | --- | --- | --- | --- | --- |
| Water | 357 | 0 | 0 | 0 | 0 | 0 | 357 | 100% | 0% |
| Land | 0 | 375 | 1 | 0 | 0 | 0 | 376 | 100% | 0% |
| *C. equisetiofolia* | 0 | 0 | 278 | 24 | 81 | 1 | 384 | 72% | 28% |
| *A. alba* | 0 | 0 | 23 | 337 | 17 | 0 | 377 | 89% | 11% |
| *N. fruticans* | 0 | 0 | 23 | 5 | 226 | 8 | 262 | 86% | 14% |
| *R. apiculata* | 0 | 0 | 36 | 0 | 36 | 351 | 423 | 83% | 17% |
| Total | 357 | 375 | 361 | 366 | 360 | 360 | 2179 |  |  |
| Producer Accuracy | 100% | 100% | 77% | 92% | 63% | 98% |  |  |  |
| Omission error | 0% | 0% | 23% | 8% | 37% | 3% |  |  |  |

| Overall Accuracy (%) | 88.30 |
| --- | --- |
| Kappa | 0.86 |
| Disagreement (%) |  |
| Quantity | 6.67 |
| Exchange | 5.92 |
| Shift | 1.53 |

### 2.1.2. Drone Imagery – 6 classes (D6PMLI 2A)

Image Source : Drone

Classification Method : Pixel Based – Maximum Likelihood

Number of land covers class : 6

Training sites : 2A

Validated by : 2B


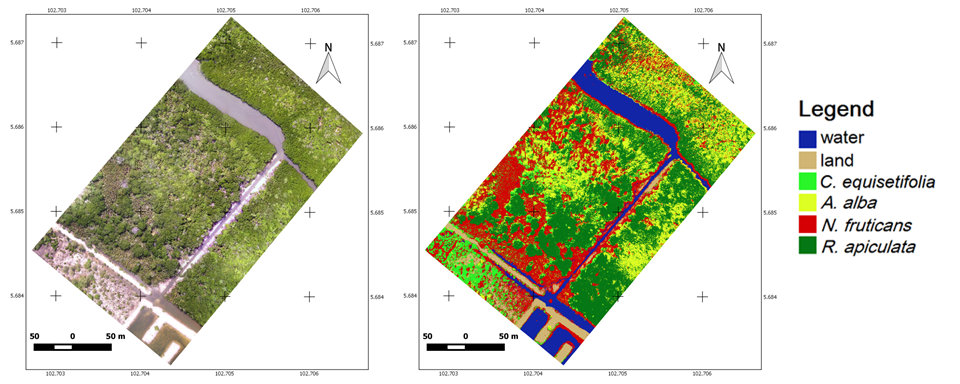


| D6PMLI 2A | Water | Land | *C. equise-tiofolia* | *A. alba* | *N. fruticans* | *R. apiculata* | Total | User Accuracy | Commission Error |
| --- | --- | --- | --- | --- | --- | --- | --- | --- | --- |
| Water | 35489 | 0 | 0 | 0 | 0 | 0 | 35489 | 100% | 0% |
| Land | 0 | 35209 | 0 | 0 | 0 | 0 | 35209 | 100% | 0% |
| *C. equisetiofolia* | 6 | 0 | 33704 | 235 | 3552 | 65 | 37562 | 90% | 10% |
| *A. alba* | 13 | 147 | 735 | 33477 | 1418 | 2894 | 38684 | 87% | 13% |
| *N. fruticans* | 326 | 316 | 938 | 437 | 31104 | 765 | 33886 | 92% | 8% |
| *R. apiculata* | 0 | 0 | 20 | 3340 | 5 | 34472 | 37837 | 91% | 9% |
| Total | 35834 | 35672 | 35397 | 37489 | 36079 | 38196 | 218667 |  |  |
| Producer Accuracy | 99% | 99% | 95% | 89% | 86% | 90% |  |  |  |
| Omission error | 1% | 1% | 5% | 11% | 14% | 10% |  |  |  |

| Overall Accuracy (%) | 93.04 |
| --- | --- |
| Kappa | 0.92 |
| Disagreement (%) |  |
| Quantity | 1.25 |
| Exchange | 5.27 |
| Shift | 2.13 |

### 2.1.3. Drone Imagery – 10 Classes (D10PMLI 4A)

Image Source : Drone

Classification Method : Pixel Based – Maximum Likelihood

Number of land covers class : 10

Training sites : 4A

Validated by : 4B


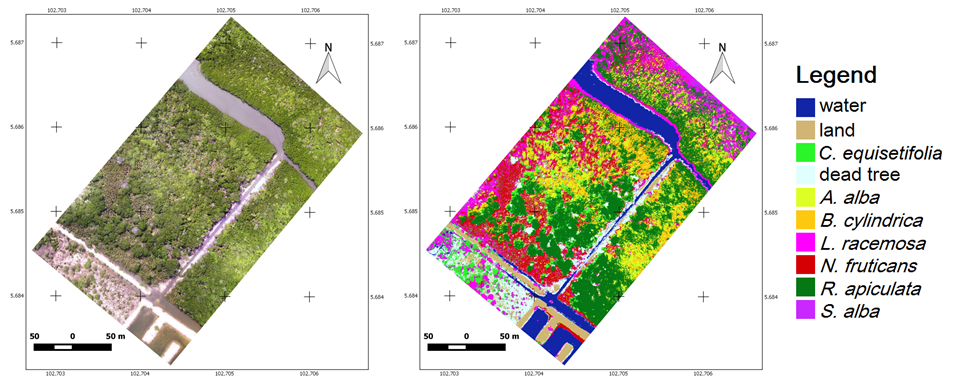


| D10PMLI 4A | Water | Land | *C. equise-tiofolia* | *Dead tree* | *A. alba* | *B. cylindrica* | *L. racemosa* | *N. fruticans* | *R. apiculata* | *S. alba* | Total | User Accuracy | Comm-  ission Error |
| --- | --- | --- | --- | --- | --- | --- | --- | --- | --- | --- | --- | --- | --- |
| Water | 34843 | 0 | 0 | 0 | 0 | 0 | 277 | 2 | 0 | 0 | 35122 | 99% | 1% |
| Land | 0 | 35408 | 0 | 21 | 0 | 0 | 3 | 0 | 0 | 0 | 35432 | 100% | 0% |
| *C. equisetiofolia* | 0 | 62 | 30728 | 695 | 630 | 704 | 2256 | 3227 | 217 | 42 | 38561 | 80% | 20% |
| *Dead tree* | 578 | 350 | 9 | 25727 | 60 | 284 | 209 | 1810 | 8 | 471 | 29506 | 87% | 13% |
| *A. alba* | 0 | 378 | 7 | 585 | 29046 | 9767 | 2458 | 876 | 1855 | 539 | 45511 | 64% | 36% |
| *B. cylindrica* | 0 | 0 | 0 | 3 | 4161 | 20116 | 307 | 11 | 1922 | 0 | 26520 | 76% | 24% |
| *L. racemosa* | 0 | 54 | 4632 | 383 | 78 | 1439 | 23026 | 5047 | 955 | 802 | 36416 | 63% | 37% |
| *N. fruticans* | 0 | 20 | 265 | 1584 | 473 | 2366 | 2012 | 24931 | 671 | 617 | 32939 | 76% | 24% |
| *R. apiculata* | 0 | 0 | 0 | 7 | 3226 | 2906 | 159 | 0 | 32711 | 0 | 39009 | 84% | 16% |
| *S. alba* | 0 | 5 | 229 | 2509 | 130 | 30 | 2343 | 651 | 0 | 6497 | 12394 | 52% | 48% |
| Total | 35421 | 36277 | 35870 | 31514 | 37804 | 37612 | 33050 | 36555 | 38339 | 8968 | 331410 |  |  |
| Producer Accuracy | 98% | 98% | 86% | 82% | 77% | 53% | 70% | 68% | 85% | 72% |  |  |  |
| Omission error | 2% | 2% | 14% | 18% | 23% | 47% | 30% | 32% | 15% | 28% |  |  |  |

| Overall Accuracy (%) | 79.37 |
| --- | --- |
| Kappa | 0.77 |
| Disagreement (%) |  |
| Quantity | 5.85 |
| Exchange | 9.39 |
| Shift | 5.92 |

## 2.2. Pixel based - Spectral Angle Mapping Algorithm

### 2.2.1. Satellite Imagery (S6PSAM 4B)

Image Source : Pleaides Satellite

Classification Method : Pixel Based – Spectral Angle Mapping

Number of land covers class : 6

Training sites : 4B

Validated by : 4A


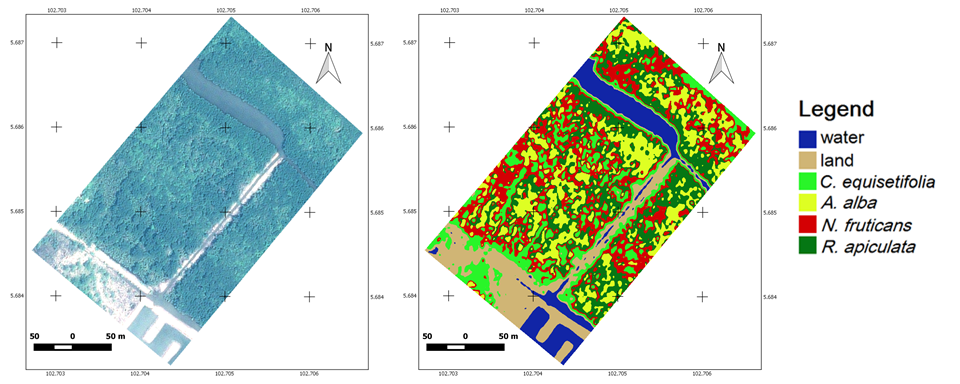


| S6PSAM 4B | Water | Land | *C. equise-tiofolia* | *A. alba* | *N. fruticans* | *R. apiculata* | Total | User Accuracy | Commission Error |
| --- | --- | --- | --- | --- | --- | --- | --- | --- | --- |
| Water | 357 | 0 | 0 | 0 | 0 | 0 | 357 | 100% | 0% |
| Land | 3 | 382 | 25 | 0 | 3 | 0 | 413 | 92% | 8% |
| *C. equisetiofolia* | 0 | 0 | 243 | 0 | 69 | 61 | 373 | 65% | 35% |
| *A. alba* | 0 | 0 | 49 | 339 | 108 | 41 | 537 | 63% | 37% |
| *N. fruticans* | 0 | 0 | 40 | 31 | 180 | 65 | 316 | 57% | 43% |
| *R. apiculata* | 0 | 0 | 1 | 0 | 0 | 193 | 194 | 99% | 1% |
| Total | 360 | 382 | 358 | 370 | 360 | 360 | 2190 |  |  |
| Producer Accuracy | 99% | 100% | 68% | 92% | 50% | 54% |  |  |  |
| Omission error | 1% | 0% | 32% | 8% | 50% | 46% |  |  |  |

| Overall Accuracy (%) | 77.35 |
| --- | --- |
| Kappa | 0.73 |
| Disagreement (%) |  |
| Quantity | 8.52 |
| Exchange | 10.24 |
| Shift | 4.15 |

### 2.2.2. Drone Imagery – 6 Classes (D6PSAM 3B)

Image Source : Drone

Classification Method : Pixel Based – Spectral Angle Mapping

Number of land covers class : 6

Training sites : 3B

Validated by : 3A


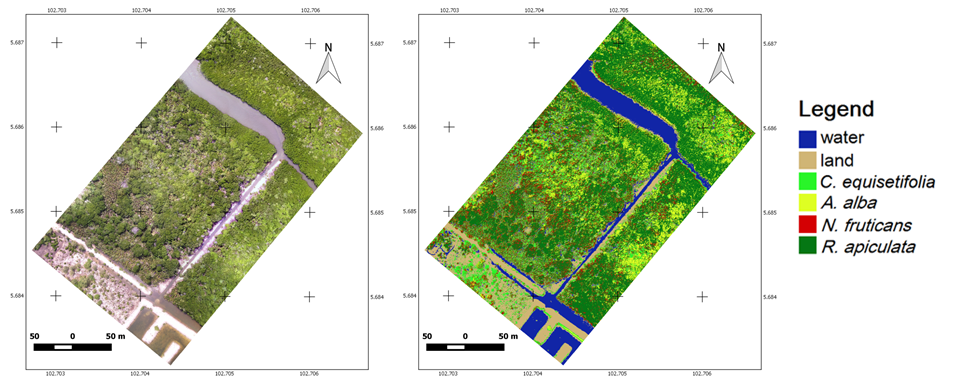


| D6PSAM 3B | Water | Land | *C. equise-tiofolia* | *A. alba* | *N. fruticans* | *R. apiculata* | Total | User Accuracy | Commission Error |
| --- | --- | --- | --- | --- | --- | --- | --- | --- | --- |
| Water | 35243 | 3 | 2 | 145 | 776 | 46 | 36215 | 97% | 3% |
| Land | 83 | 35966 | 906 | 1023 | 3935 | 55 | 41968 | 86% | 14% |
| *C. equisetiofolia* | 0 | 30 | 27172 | 2960 | 9847 | 1451 | 41460 | 66% | 34% |
| *A. alba* | 0 | 0 | 1481 | 26075 | 417 | 1574 | 29547 | 88% | 12% |
| *N. fruticans* | 0 | 0 | 4988 | 643 | 9353 | 3498 | 18482 | 51% | 49% |
| *R. apiculata* | 0 | 0 | 268 | 6479 | 11913 | 32517 | 51177 | 64% | 36% |
| Total | 35326 | 35999 | 34817 | 37325 | 36241 | 39141 | 218849 |  |  |
| Producer Accuracy | 100% | 100% | 78% | 70% | 26% | 83% |  |  |  |
| Omission error | 0% | 0% | 22% | 30% | 74% | 17% |  |  |  |

| Overall Accuracy (%) | 76.00 |
| --- | --- |
| Kappa | 0.71 |
| Disagreement (%) |  |
| Quantity | 14.08 |
| Exchange | 13.48 |
| Shift | 0.84 |

### 2.2.3. Drone Imagery – 10 Classes (D10PSAM 3B)

Image Source : Drone

Classification Method : Pixel Based – Spectral Angle Mapping

Number of land covers class : 10

Training sites : 3B

Validated by : 3A


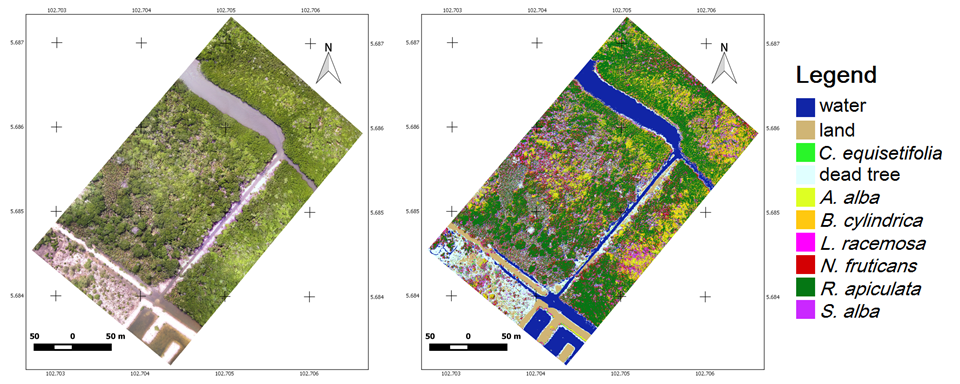


| D10PSAM 3B | Water | Land | *C. equise-tiofolia* | *Dead tree* | *A. alba* | *B. cylindrica* | *L. racemosa* | *N. fruticans* | *R. apiculata* | *S. alba* | Total | User Accuracy | Commi-ssion Error |
| --- | --- | --- | --- | --- | --- | --- | --- | --- | --- | --- | --- | --- | --- |
| Water | 38710 | 1541 | 0 | 1039 | 109 | 24 | 946 | 387 | 35 | 93 | 42884 | 90% | 10% |
| Land | 0 | 28262 | 19 | 1001 | 766 | 81 | 31 | 181 | 3 | 364 | 30708 | 92% | 8% |
| *C. equisetiofolia* | 3 | 19 | 14884 | 2026 | 395 | 582 | 2596 | 2186 | 336 | 1588 | 24615 | 60% | 40% |
| *Dead tree* | 464 | 1820 | 885 | 16757 | 645 | 649 | 3482 | 4534 | 173 | 1770 | 31179 | 54% | 46% |
| *A. alba* | 0 | 0 | 1519 | 9 | 19841 | 8679 | 2472 | 231 | 609 | 95 | 33455 | 59% | 41% |
| *B. cylindrica* | 0 | 0 | 1262 | 1 | 11245 | 11956 | 4304 | 279 | 5468 | 32 | 34547 | 35% | 65% |
| *L. racemosa* | 0 | 14 | 6249 | 160 | 505 | 1206 | 3263 | 1034 | 657 | 692 | 13780 | 24% | 76% |
| *N. fruticans* | 0 | 0 | 5226 | 1236 | 157 | 966 | 2389 | 5123 | 3263 | 266 | 18626 | 28% | 72% |
| *R. apiculata* | 0 | 0 | 1095 | 419 | 6125 | 9079 | 3909 | 11323 | 41971 | 42 | 73963 | 57% | 43% |
| *S. alba* | 7 | 0 | 8139 | 2423 | 4727 | 3743 | 2953 | 3721 | 1733 | 3293 | 30739 | 11% | 89% |
| Total | 39184 | 31656 | 39278 | 25071 | 44515 | 36965 | 26345 | 28999 | 54248 | 8235 | 334496 |  |  |
| Producer Accuracy | 99% | 89% | 38% | 67% | 45% | 32% | 12% | 18% | 77% | 40% |  |  |  |
| Omission error | 1% | 11% | 62% | 33% | 55% | 68% | 88% | 82% | 23% | 60% |  |  |  |

| Overall Accuracy (%) | 55.03 |
| --- | --- |
| Kappa | 0.49 |
| Disagreement (%) |  |
| Quantity | 22.17 |
| Exchange | 16.22 |
| Shift | 9.04 |

## 2.3. Object Based - Manual Classification

### 2.3.1. Satellite Imagery (S6OMAN 5A)

Image Source : Pleaides Satellite

Classification Method : Object Based – Manual

Number of land covers class : 6

Training sites : -

Validated by : 5A


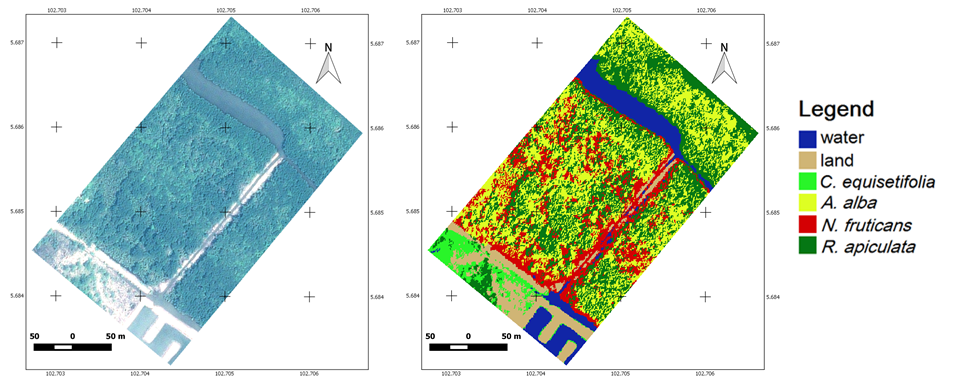


| S6OMAN 5A | Water | Land | *C. equise-tiofolia* | *A. alba* | *N. fruticans* | *R. apiculata* | Total | User Accuracy | Commission Error |
| --- | --- | --- | --- | --- | --- | --- | --- | --- | --- |
| Water | 535 | 0 | 0 | 0 | 0 | 0 | 535 | 100% | 0% |
| Land | 1 | 414 | 0 | 0 | 0 | 0 | 415 | 100% | 0% |
| *C. equisetiofolia* | 0 | 0 | 161 | 0 | 0 | 0 | 161 | 100% | 0% |
| *A. alba* | 0 | 0 | 0 | 516 | 297 | 117 | 930 | 55% | 45% |
| *N. fruticans* | 47 | 0 | 0 | 0 | 199 | 46 | 292 | 68% | 32% |
| *R. apiculata* | 1 | 0 | 60 | 22 | 14 | 297 | 394 | 75% | 25% |
| Total | 584 | 414 | 221 | 538 | 510 | 460 | 2727 |  |  |
| Producer Accuracy | 92% | 100% | 73% | 96% | 39% | 65% |  |  |  |
| Omission error | 8% | 0% | 27% | 4% | 61% | 35% |  |  |  |

| Overall Accuracy (%) | 77.81 |
| --- | --- |
| Kappa | 0.73 |
| Disagreement (%) |  |
| Quantity | 14.13 |
| Exchange | 6.05 |
| Shift | 7.09 |

### 2.3.2. Drone Imagery – 6 classes (D6OMAN 2B)

Image Source : Drone

Classification Method : Object Based – Manual

Number of land covers class : 6

Training sites : -

Validated by : 2B


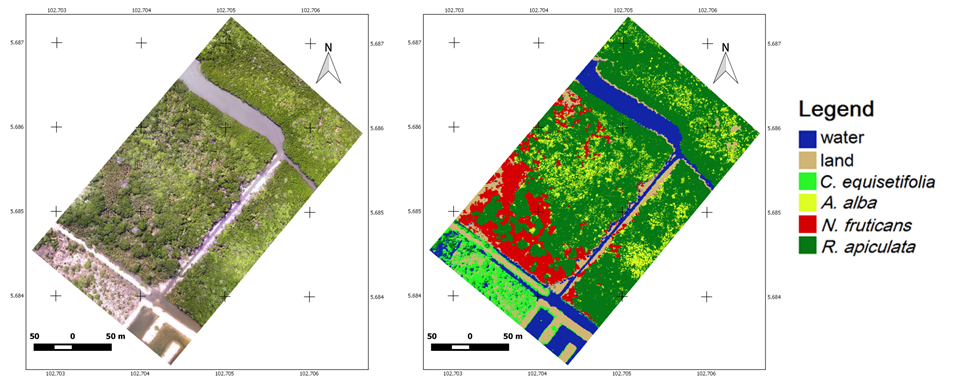


| D60MAN 2B | Water | Land | *C. equise-tiofolia* | *A. alba* | *N. fruticans* | *R. apiculata* | Total | User Accuracy | Commission Error |
| --- | --- | --- | --- | --- | --- | --- | --- | --- | --- |
| Water | 4001 | 0 | 10 | 0 | 0 | 0 | 4011 | 100% | 0% |
| Land | 34 | 4046 | 0 | 0 | 83 | 0 | 4163 | 97% | 3% |
| *C. equisetiofolia* | 0 | 13 | 3899 | 0 | 0 | 0 | 3912 | 100% | 0% |
| *A. alba* | 14 | 3 | 0 | 3188 | 0 | 31 | 3236 | 99% | 1% |
| *N. fruticans* | 0 | 0 | 0 | 0 | 3988 | 0 | 3988 | 100% | 0% |
| *R. apiculata* | 0 | 0 | 71 | 1021 | 0 | 4255 | 5347 | 80% | 20% |
| Total | 4049 | 4062 | 3980 | 4209 | 4071 | 4286 | 24657 |  |  |
| Producer Accuracy | 99% | 100% | 98% | 76% | 98% | 99% |  |  |  |
| Omission error | 1% | 0% | 2% | 24% | 2% | 1% |  |  |  |

| Overall Accuracy (%) | 94.81 |
| --- | --- |
| Kappa | 0.94 |
| Disagreement (%) |  |
| Quantity | 11.26 |
| Exchange | 0.19 |
| Shift | 0.13 |

### 2.3.3. Drone Imagery – 10 Classes (D10OMAN 4B)

Image Source : Drone

Classification Method : Object Based – Manual

Number of land covers class : 10

Training sites : -

Validated by : 4B


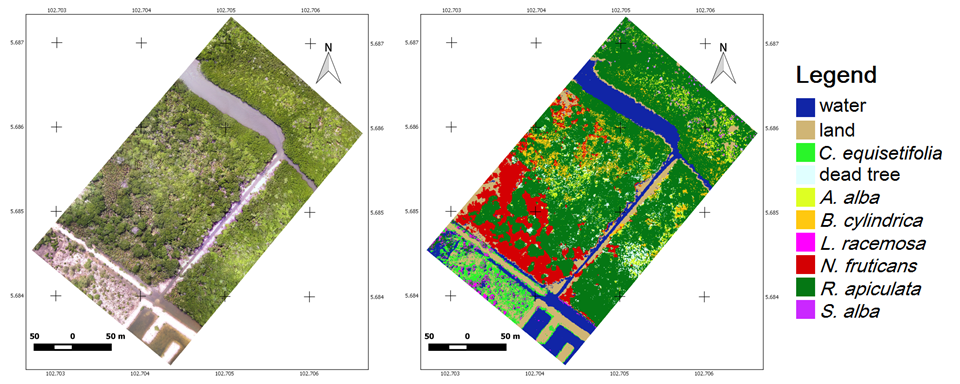


| D10MAN 4B | Water | Land | *C. equise-tiofolia* | *Dead tree* | *A. alba* | *B. cylindrica* | *L. racemosa* | *N. fruticans* | *R. apiculata* | *S. alba* | Total | User Accuracy | Commi-ssion Error |
| --- | --- | --- | --- | --- | --- | --- | --- | --- | --- | --- | --- | --- | --- |
| Water | 3912 | 0 | 10 | 0 | 0 | 0 | 28 | 0 | 0 | 0 | 3950 | 99% | 1% |
| Land | 46 | 4129 | 0 | 0 | 0 | 2 | 61 | 15 | 0 | 0 | 4253 | 97% | 3% |
| *C. equisetiofolia* | 0 | 22 | 2898 | 0 | 0 | 0 | 1475 | 0 | 0 | 0 | 4395 | 66% | 34% |
| *Dead tree* | 0 | 0 | 0 | 2160 | 386 | 261 | 0 | 0 | 24 | 266 | 3097 | 70% | 30% |
| *A. alba* | 0 | 3 | 0 | 410 | 1949 | 357 | 0 | 0 | 3 | 22 | 2744 | 71% | 29% |
| *B. cylindrica* | 0 | 0 | 0 | 218 | 1019 | 1571 | 0 | 0 | 51 | 0 | 2859 | 55% | 45% |
| *L. racemosa* | 0 | 0 | 936 | 0 | 0 | 0 | 1229 | 0 | 0 | 0 | 2165 | 57% | 43% |
| *N. fruticans* | 0 | 0 | 0 | 102 | 0 | 5 | 509 | 4119 | 0 | 0 | 4735 | 87% | 13% |
| *R. apiculata* | 0 | 0 | 146 | 593 | 890 | 1998 | 428 | 0 | 4226 | 137 | 8418 | 50% | 50% |
| *S. alba* | 0 | 0 | 0 | 67 | 0 | 0 | 0 | 0 | 0 | 591 | 658 | 90% | 10% |
| Total | 3958 | 4154 | 3990 | 3550 | 4244 | 4194 | 3730 | 4134 | 4304 | 1016 | 37274 |  |  |
| Producer Accuracy | 99% | 99% | 73% | 61% | 46% | 37% | 33% | 100% | 98% | 58% |  |  |  |
| Omission error | 1% | 1% | 27% | 39% | 54% | 63% | 67% | 0% | 2% | 42% |  |  |  |

| Overall Accuracy (%) | 71.86 |
| --- | --- |
| Kappa | 0.68 |
| Disagreement (%) |  |
| Quantity | 28.06 |
| Exchange | 3.60 |
| Shift | 2.54 |

## 2.4. Pixel Based - Automatic Algorithm

### 2.4.1. Satellite Imagery (S6OAUT 1B)

Image Source : Pleaides Satellite

Classification Method : Object Based – Automatic

Number of land covers class : 6

Training sites : 1B

Validated by : 1A


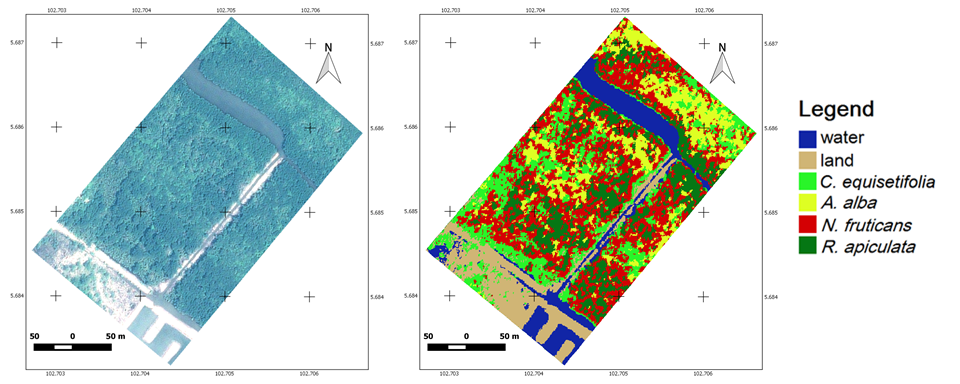


| S6OAUT 1B | Water | Land | *C. equise-tiofolia* | *A. alba* | *N. fruticans* | *R. apiculata* | Total | User Accuracy | Commission Error |
| --- | --- | --- | --- | --- | --- | --- | --- | --- | --- |
| Water | 433 | 0 | 0 | 0 | 6 | 0 | 439 | 99% | 1% |
| Land | 1 | 462 | 39 | 0 | 32 | 0 | 534 | 87% | 13% |
| *C. equisetiofolia* | 0 | 0 | 390 | 7 | 326 | 4 | 727 | 54% | 46% |
| *A. alba* | 0 | 0 | 21 | 444 | 0 | 16 | 481 | 92% | 8% |
| *N. fruticans* | 0 | 0 | 5 | 0 | 78 | 196 | 279 | 28% | 72% |
| *R. apiculata* | 0 | 0 | 0 | 0 | 0 | 252 | 252 | 100% | 0% |
| Total | 434 | 462 | 455 | 451 | 442 | 468 | 2712 |  |  |
| Producer Accuracy | 100% | 100% | 86% | 98% | 18% | 54% |  |  |  |
| Omission error | 0% | 0% | 14% | 2% | 82% | 46% |  |  |  |

| Overall Accuracy (%) | 75.92 |
| --- | --- |
| Kappa | 0.71 |
| Disagreement (%) |  |
| Quantity | 21.74 |
| Exchange | 1.42 |
| Shift | 9.44 |

### 2.4.2. Drone Imagery – 6 Classes (D6OAUT 5A)

Image Source : Drone

Classification Method : Object Based – Automatic

Number of land covers class : 6

Training sites : 5A

Validated by : 5B


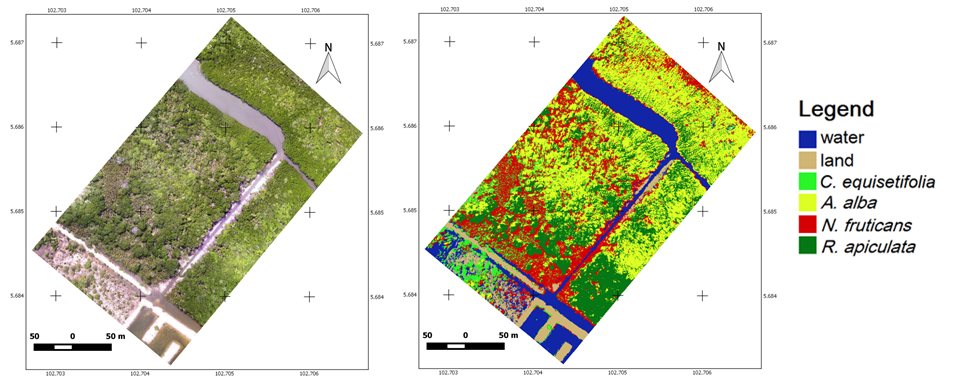


| D60AUT 5A | Water | Land | *C. equise-tiofolia* | *A. alba* | *N. fruticans* | *R. apiculata* | Total | User Accuracy | Commission Error |
| --- | --- | --- | --- | --- | --- | --- | --- | --- | --- |
| Water | 4010 | 11 | 166 | 0 | 0 | 0 | 4187 | 96% | 4% |
| Land | 6 | 4050 | 3 | 0 | 0 | 0 | 4059 | 100% | 0% |
| *C. equisetiofolia* | 0 | 0 | 3460 | 113 | 809 | 0 | 4382 | 79% | 21% |
| *A. alba* | 0 | 0 | 28 | 4041 | 137 | 1828 | 6034 | 67% | 33% |
| *N. fruticans* | 0 | 0 | 271 | 17 | 3207 | 25 | 3520 | 91% | 9% |
| *R. apiculata* | 0 | 0 | 0 | 74 | 0 | 2434 | 2508 | 97% | 3% |
| Total | 4016 | 4061 | 3928 | 4245 | 4153 | 4287 | 24690 |  |  |
| Producer Accuracy | 100% | 100% | 88% | 95% | 77% | 57% |  |  |  |
| Omission error | 0% | 0% | 12% | 5% | 23% | 43% |  |  |  |

| Overall Accuracy (%) | 85.87 |
| --- | --- |
| Kappa | 0.83 |
| Disagreement (%) |  |
| Quantity | 10.70 |
| Exchange | 4.61 |
| Shift | 0.29 |

### 2.4.3. Drone Imagery – 10 Classed (D10OAUT 5A)

Image Source : Drone

Classification Method : Object Based – Automatic

Number of land covers class : 10

Training sites : 5A

Validated by : 5B


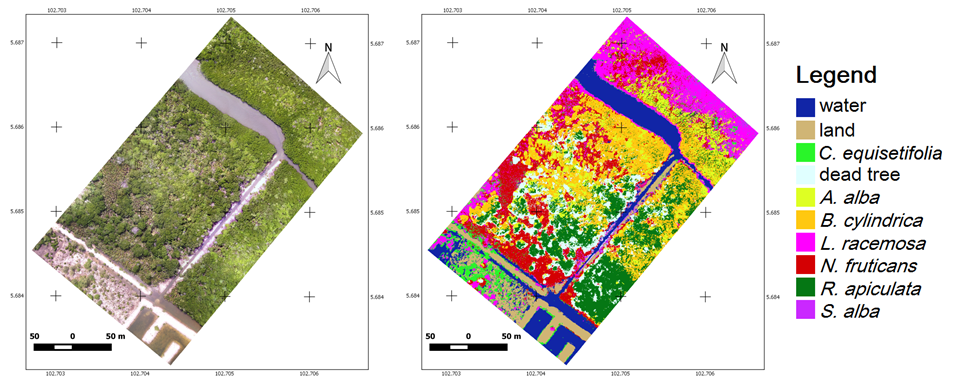


| D10OAUT 5A | Water | Land | *C. equise-tiofolia* | *Dead tree* | *A. alba* | *B. cylindrica* | *L. racemosa* | *N. fruticans* | *R. apiculata* | *S. alba* | Total | User Accuracy | Commi-ssion Error |
| --- | --- | --- | --- | --- | --- | --- | --- | --- | --- | --- | --- | --- | --- |
| Water | 4006 | 0 | 1 | 0 | 0 | 0 | 18 | 0 | 0 | 0 | 4025 | 100% | 0% |
| Land | 2 | 3914 | 3 | 223 | 0 | 0 | 9 | 0 | 0 | 0 | 4151 | 94% | 6% |
| *C. equisetiofolia* | 0 | 9 | 2138 | 50 | 0 | 0 | 878 | 48 | 0 | 53 | 3176 | 67% | 33% |
| *Dead tree* | 0 | 0 | 851 | 2729 | 273 | 34 | 0 | 203 | 125 | 6 | 4221 | 65% | 35% |
| *A. alba* | 0 | 0 | 184 | 1 | 2112 | 1418 | 296 | 2 | 255 | 0 | 4268 | 49% | 51% |
| *B. cylindrica* | 0 | 0 | 250 | 1 | 1397 | 2472 | 120 | 10 | 1387 | 0 | 5637 | 44% | 56% |
| *L. racemosa* | 0 | 0 | 52 | 18 | 98 | 10 | 2030 | 160 | 248 | 164 | 2780 | 73% | 27% |
| *N. fruticans* | 0 | 0 | 455 | 357 | 304 | 186 | 529 | 3631 | 223 | 55 | 5740 | 63% | 37% |
| *R. apiculata* | 0 | 0 | 0 | 0 | 23 | 20 | 0 | 0 | 1999 | 0 | 2042 | 98% | 2% |
| *S. alba* | 0 | 0 | 0 | 479 | 4 | 3 | 104 | 92 | 12 | 675 | 1369 | 49% | 51% |
| Total | 4008 | 3923 | 3934 | 3858 | 4211 | 4143 | 3984 | 4146 | 4249 | 953 | 37409 |  |  |
| Producer Accuracy | 100% | 100% | 54% | 71% | 50% | 60% | 51% | 88% | 47% | 71% |  |  |  |
| Omission error | 0% | 0% | 46% | 29% | 50% | 40% | 49% | 12% | 53% | 29% |  |  |  |

| Overall Accuracy (%) | 68.72 |
| --- | --- |
| Kappa | 0.65 |
| Disagreement (%) |  |
| Quantity | 11.40 |
| Exchange | 11.22 |
| Shift | 6.41 |

# 3. Separability of land-cover classes based on pixel-based classification

Matrix showing the separability of land-cover classes in Setiu wetland with respect to – (A) Pleiades 1B satellite imagery for dominant land-cover classes, (B) Drone imagery for dominant land-cover classes and, (C) Drone imagery for dominant + non-dominant land-cover classes. Values above the grey shaded diagonal squares are Jeffries-Matusita distances, which were calculated using Maximum Likelihood algorithm, and below are spectral angle distances, which were calculated using Spectral Angle Mapping algorithm. The values highlighted in bold font shows lower separability between two land-cover types. CE = *Casuarina equisetifolis*; AA = *Avicennia alba*; NF = *Nypa fruticans*; RA = *Rhizophora apiculata*; BC = *Bruguiera cylindrica*; LR = *Lumnitzera racemosa*; SA = *Sonneratia alba*

| (A) Pleiades-1B with dominant land-cover types | | | | | | | | | | | |  | (B) DJI Phantom 2 drone with dominant land-cover types | | | | | | | | | | | |
| --- | --- | --- | --- | --- | --- | --- | --- | --- | --- | --- | --- | --- | --- | --- | --- | --- | --- | --- | --- | --- | --- | --- | --- | --- |
|  | Water | | Land | | CE | | AA | NF | | RA | |  |  | | Water | | Land | CE | | AA | | NF | | RA |
| Water |  | | 1.999 | | 1.951 | | 2.000 | 2.000 | | 2.000 | |  | Water | |  | | 2.000 | 2.000 | | 2.000 | | 2.000 | | 2.000 |
| Land | 16.992 | |  | | 1.999 | | 2.000 | 2.000 | | 2.000 | |  | Land | | 35.049 | |  | 2.000 | | 2.000 | | 2.000 | | 2.000 |
| CE | 30.137 | | 16.360 | |  | | 1.527 | 0.623 | | 1.737 | |  | CE | | 45.757 | | 13.695 |  | | 1.877 | | 1.427 | | 1.958 |
| AA | 37.229 | | 22.971 | | 7.097 | |  | 1.707 | | 1.998 | |  | AA | | 48.132 | | 16.062 | 5.681 | |  | | 1.910 | | 1.444 |
| NF | 32.380 | | 18.507 | | 2.268 | | 4.871 |  | | 1.792 | |  | NF | | 49.681 | | 17.920 | 4.369 | | 6.659 | |  | | 1.963 |
| RA | 35.396 | | 21.673 | | 5.433 | | 2.354 | 3.196 | |  | |  | RA | | 57.778 | | 25.668 | 12.698 | | 10.797 | | 8.941 | |  |
| (C) DJI Phantom 2 drone with dominant + non-dominant land-cover types | | | | | | | | | | | | | | | | | | | | | | | | |
|  | | water | | land | | CE | | | AA | | NF | | | RA | | Dead tree | | | BC | | LR | | SA | |
| Water | |  | | 2.0000 | | 2.0000 | | | 2.0000 | | 1.9999 | | | 2.0000 | | 1.9995 | | | 2.0000 | | 1.9994 | | 2.0000 | |
| Land | | 35.0490 | |  | | 2.0000 | | | 2.0000 | | 2.0000 | | | 2.0000 | | 1.9972 | | | 2.0000 | | 2.0000 | | 1.9999 | |
| CE | | 45.7567 | | 13.6953 | |  | | | 1.8767 | | 1.4266 | | | 1.9583 | | 1.8554 | | | 1.8711 | | 1.4019 | | 1.7721 | |
| AA | | 48.1317 | | 16.0623 | | 5.6814 | | |  | | 1.9097 | | | 1.4438 | | 1.9708 | | | 0.7263 | | 1.8257 | | 1.9712 | |
| NF | | 49.6813 | | 17.9199 | | 4.3687 | | | 6.6592 | |  | | | 1.9628 | | 1.5724 | | | 1.8277 | | 1.0643 | | 1.7091 | |
| RA | | 57.7780 | | 25.6682 | | 12.6980 | | | 10.7967 | | 8.9414 | | |  | | 1.9939 | | | 1.3967 | | 1.9294 | | 1.9982 | |
| Dead tree | | 36.8944 | | 8.0676 | | 9.6921 | | | 13.9570 | | 13.3648 | | | 21.9555 | |  | | | 1.9600 | | 1.8294 | | 1.7424 | |
| BC | | 49.9380 | | 18.0851 | | 6.2215 | | | 2.4392 | | 5.5200 | | | 8.5012 | | 15.2251 | | |  | | 1.7877 | | 1.9760 | |
| LR | | 46.4463 | | 14.3277 | | 1.7920 | | | 4.0768 | | 4.3677 | | | 11.7401 | | 10.8213 | | | 4.6375 | |  | | 1.3886 | |
| SA | | 41.1031 | | 11.5780 | | 5.4407 | | | 9.5731 | | 8.8273 | | | 17.0601 | | 5.7652 | | | 10.3856 | | 6.2270 | |  | |

# 4. Relative Intensity of RGB and IR cameras in various wavelengths


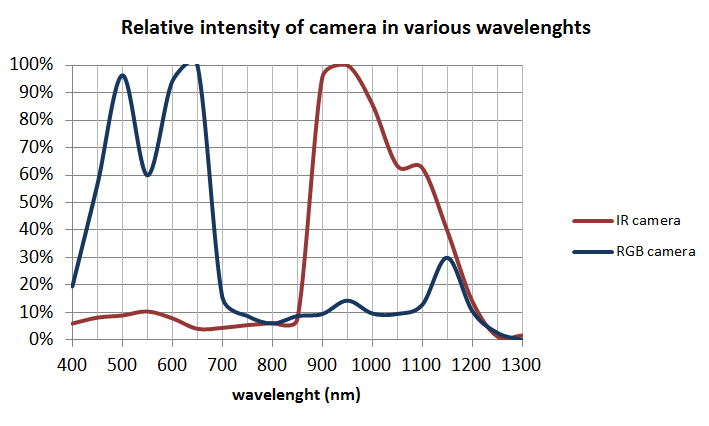


# 5. Accuracy of drone imagery across various resolutions

(OA = Overall Accuracy; PA = Producer Accuracy; UA = user


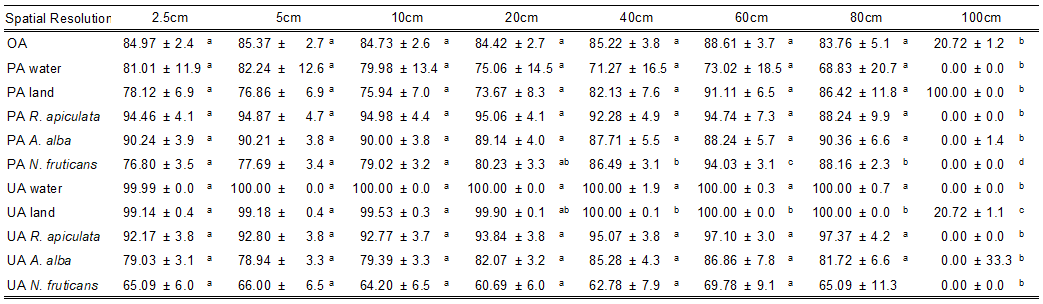

Supplement: S1 File — Detailed information on classification analysis, including iteration results, statistical analysis and error matrices. (DOCX) [file pone.0200288.s001.docx]
