# Supplementary material for: The advantages of using drones over space-borne imagery in the mapping of mangrove forests
Source: PLoS One. 2018 Jul 18;13(7):e0200288. doi: 10.1371/journal.pone.0200288 (PMC6051606; doi:10.1371/journal.pone.0200288)
Supplement: S2 File — Stepwise protocol of image analysis. (DOCX) [file pone.0200288.s002.docx]

**The advantages of using drones over space-borne imagery in the mapping of mangrove forests**

**Data Analysis Protocol**

Contents

[A. Geotagging Images 1](#_Toc515352308)

[B. Generating Mosaic Image 4](#_Toc515352309)

[C. Image joining and georeferencing with satellite image 7](#_Toc515352310)

[D. Paralax and Invalid DEM Cropping 10](#_Toc515352311)

[E. ROI creation 14](#_Toc515352312)

[F. Segmentation 18](#_Toc515352313)

[G. Object-based Manual Classification 21](#_Toc515352314)

[H. Object-based Automatic Classification 23](#_Toc515352315)

[I. Merging Classified Vector and Convert to Raster 28](#_Toc515352316)

[J. Pixel-based Maximum Likelihood and Spectral Angle Mapping Classification 31](#_Toc515352317)

[K. Accuracy Analysis 32](#_Toc515352318)

[L. Pontius Matrix 33](#_Toc515352319)

# Geotagging Images

- 1. Take out the memory card from Flytrex device. Open it on a computer and copy the flight log data with extension .fvp
  2. Create an account and sign-in at [www.flytrex.com](http://www.flytrex.com). On the profile page, click “Upload Mission”, and upload the .fpv file


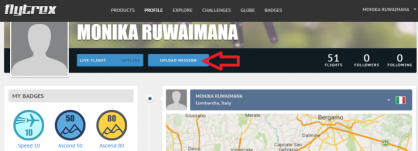


- 1. On the uploaded mission, click drop-down-arrow next to the country flag, and choose “Export Mission”. Then select option “Download CSV”


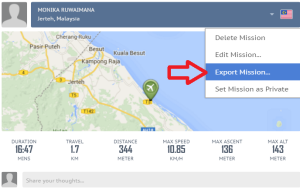


- 1. Open the .csv file using Microsoft Excel. This file contains complete flight information. Delete the columns, except latitude, longitude, altitude feet, and datetime. Divide datetime column into date and time. The original time is in GMT time, therefore, change the hour so it fits with the local time (in this case, add 8 hour as Malaysia is GMT+8). Save the file and close the Excel.


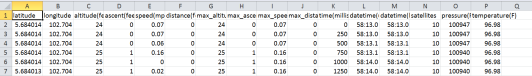

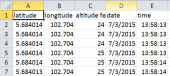


- 1. Open the file with Notepad, select all text and copy it. Open [www.gpsvisualizer.com](http://www.gpsvisualizer.com), select option “Convert to GPX”


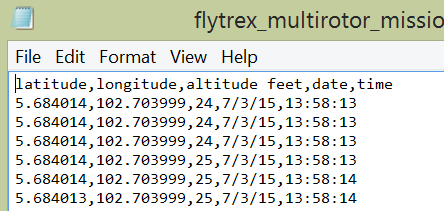

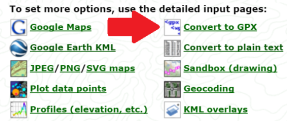


- 1. Paste the text data from .csv file on the window “paste your data here”. Choose comma as “Plain text delimiter” and click “Convert”


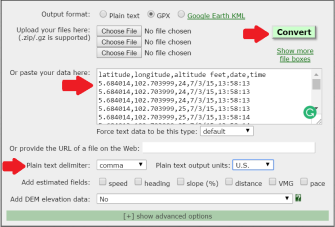


- 1. Click the provided link and download the .gpx data.


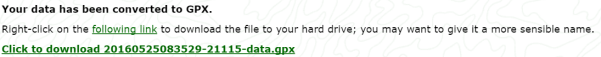


- 1. Download and install free software GeoSetter from <http://www.geosetter.de/en/>. Open the GeoSetter. On the upper left directory dropdown, select the folder containing the drone images. On the bottom right, click the icon open and open the .gpx file from the previous step. A Google map on the upper right window will show the drone track.


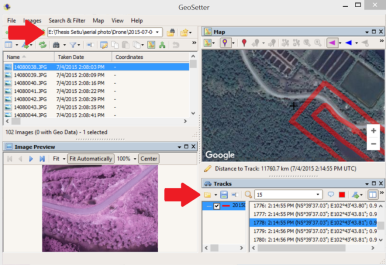


- 1. Select an image which is located more or less in the middle of image list, and select the record with same minutes and seconds from the window “Tracks”. Click shortcut ctrl-A to select all the images


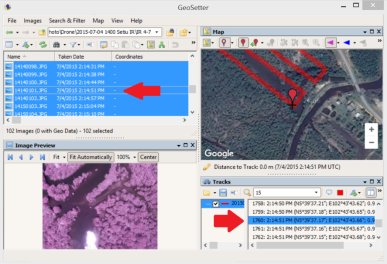


- 1. Click menu “Images”, then choose “Synchronize with GPS data file” or just push the shortcut button ctrl-G to open Synchronize window. Click “OK” button on the bottom left. Then click “Yes” on the pop-up window.


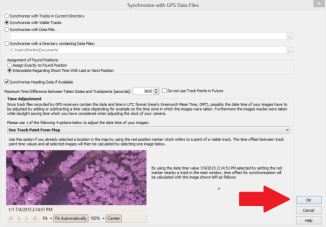

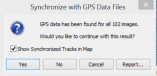


- 1. The coordinates for each image will appear. Click the save icon. Images are tagged and ready for processing.


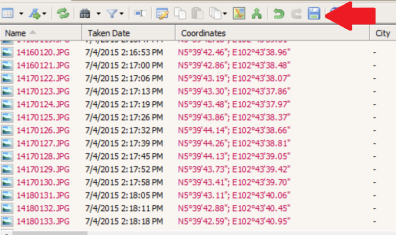


# Generating Mosaic Image

1. Install Agisoft Photoscan Professional with license.
2. Click the menu “Workflow”, then “Add Photos”. Choose all the geotagged images from the directory.


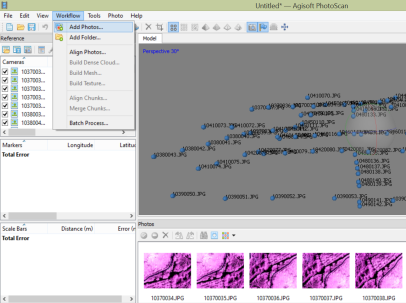


1. Click the menu “Workflow”, then “Align photos”. Choose the “Accuracy” setting “Low”, then click “OK”. Wait for the processing to finish.


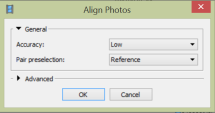

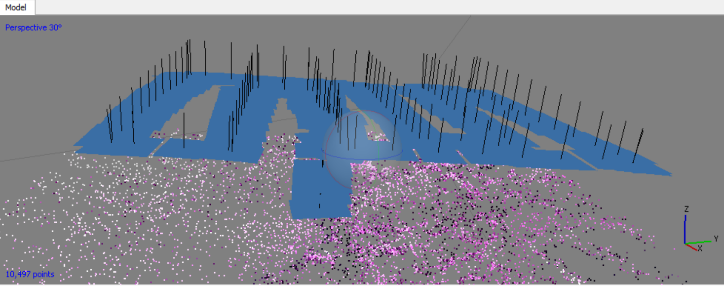


1. Click the menu “Workflow”, then “Build Dense Cloud”. Choose the “Quality” setting “Low”, then click OK. Wait for the processing to finish.


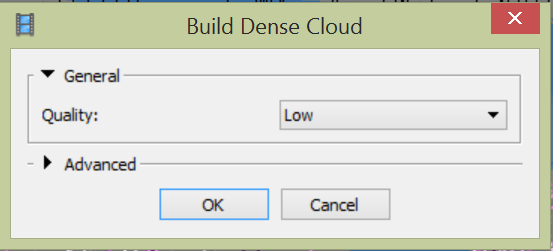

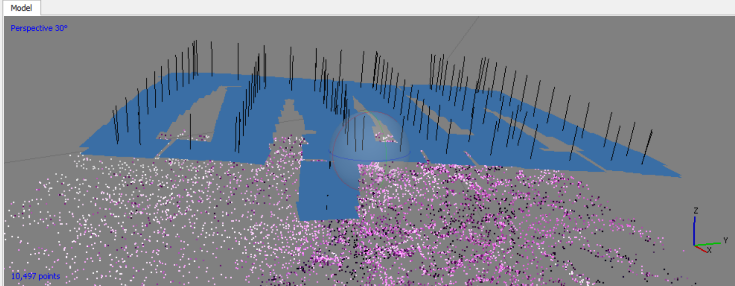


1. Click the menu “Workflow” then “Build Mesh”. Choose the “Face count” setting “Low”, then click “OK”. Wait for the processing to finish.


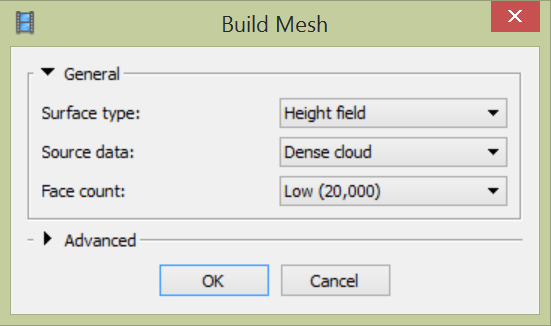

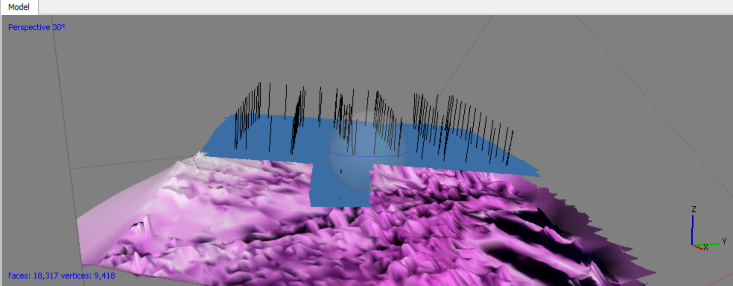


1. Click the menu “Workflow” then “Build Texture”. Click “OK”. Wait for the processing to finish.


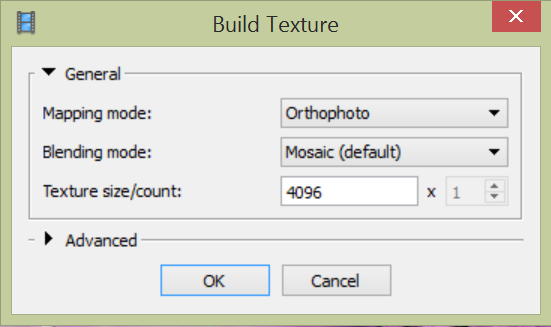

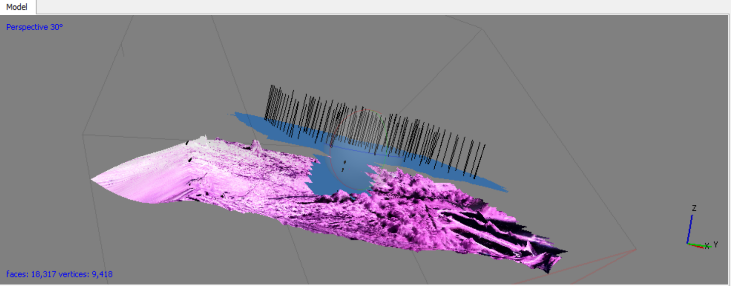


1. Click the icon camera
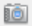
 for clear view of the image.
2. Open Google Earth, and zoom into the exact same area. Find a corresponding feature which can be useful for creating the control point.


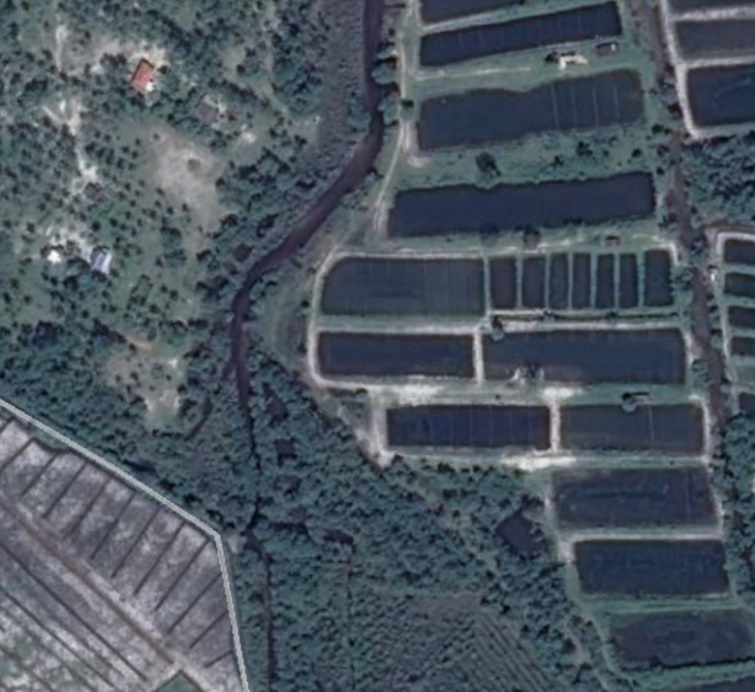

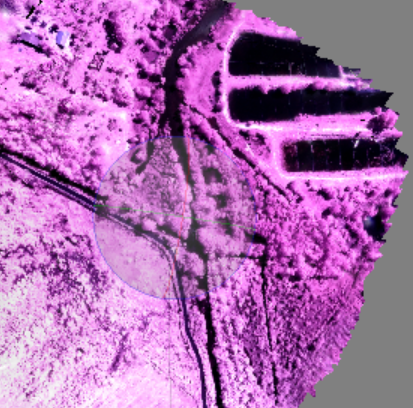


1. On Agisoft, right click on the point and click “Create Marker”. A flag will appear on the image and a marker named “point 1” will appear on the Markers Panel on the left side of the screen.


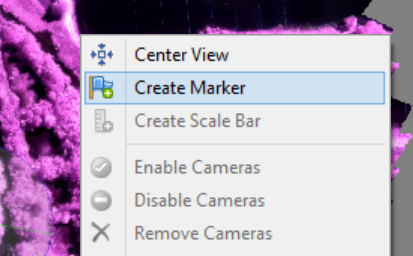

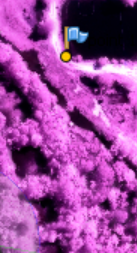

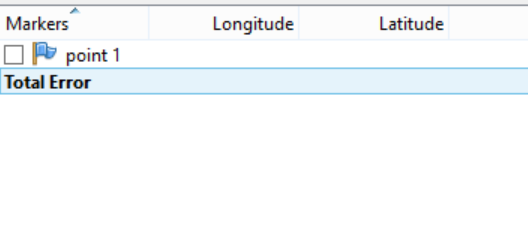


1. On Google Earth, place a mark over the correspond point, and copy the latitude and longitude values to the Markers Panel in Agisoft. The coordinates must be in decimal degree format.


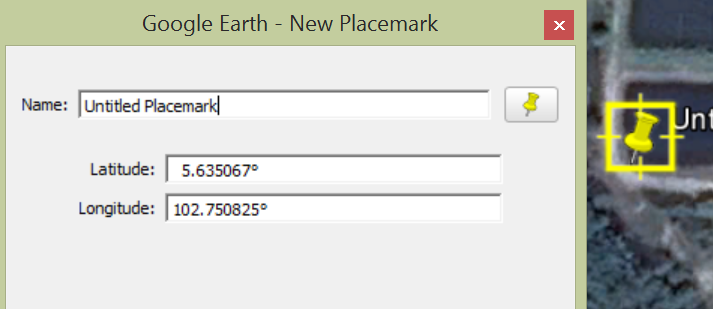

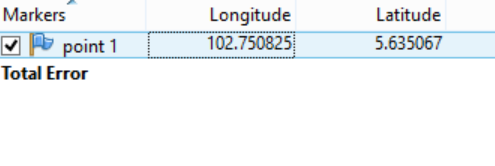


1. Repeat the process until at least 5 control points generated.
2. On the panel “Reference”, located on the upper left, select all the cameras, then right click and choose “Reset Camera Alignment”, click “Yes” on the pop up window


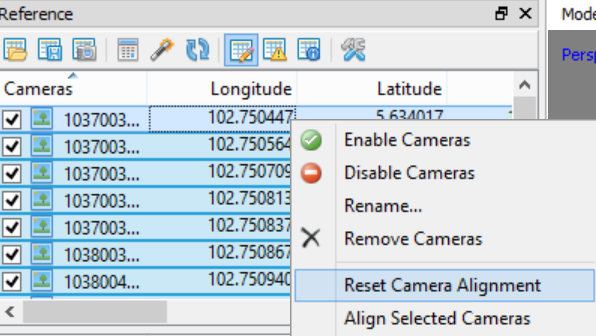


1. Repeat the “Workflow” process from “Align Photos” to “Build Texture”, but with setting medium or high. This depends on the capacity of the computer; using a computer with 2.6GHz processor and 8GB RAM, medium setting will take approximately 6 hours and high setting approximately 20 hours. This research was using medium setting.
2. Click menu “File” then “Export Orthophoto” then “Export JPEG/TIF..”. On the pop up window, choose coordinate system WGS 84/UTM zone 48N (Setiu’s UTM zone). On the “Pixel size”, fill 0.05 to give 5cm resolution. Tick the “Write World file” box, and click “Export”. Give name to the file and then click “Save”


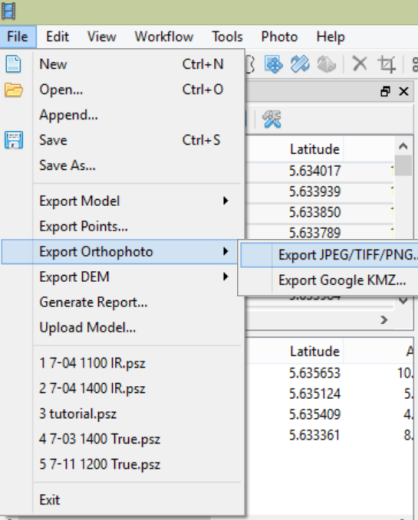

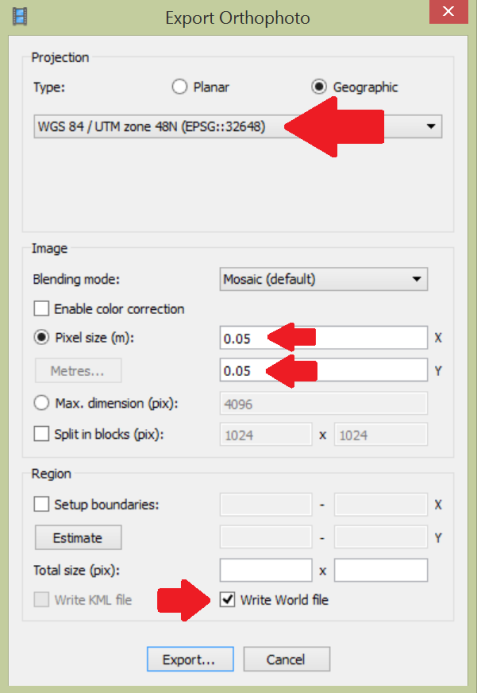


1. Click menu “File” then “Export DEM” then “Export TIFF/BIL...”. On the pop up window, choose coordinate system WGS 84/UTM zone 48N (Setiu’s UTM zone). On the “Pixel size”, fill 0.05 to give 5cm resolution. Tick the”Write World file” box, and click “Export”. Give a name to the file and then click “Save”


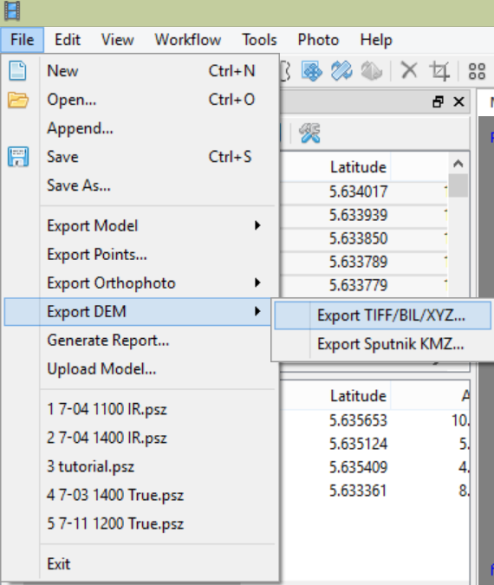

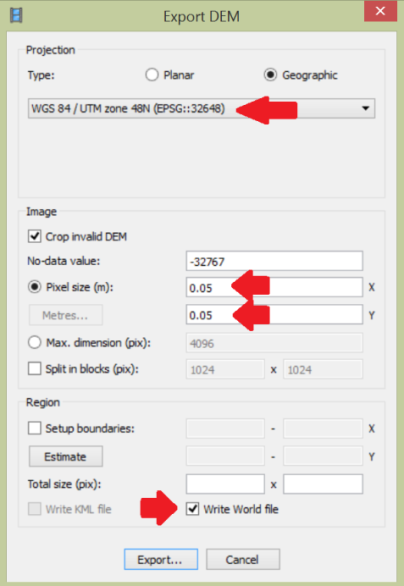


# Image joining and georeferencing with satellite image

1. Download and install QGIS OSGeo4W advance package from [www.qgis.org](http://www.qgis.org). This package is including ORFEO Toolbox and SAGA GIS.
2. Open QGIS. On the bottom left of “Processing Toolbox” panel, choose “Advanced Interface”


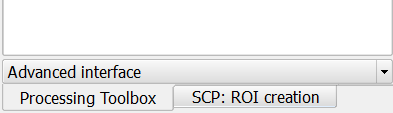


1. Click menu “Layer” then “Add Layer” then “Add Raster Layer” or by clicking shortcut Ctrl-Shift-R. Open 3 complementary mosaic images : RGB, IR and DEM (for DEM, choose one of the RGB or IR, this research was using DEM from RGB, because RGB camera have a higher number of images on the same area, therefore, providing more detailed DEM)


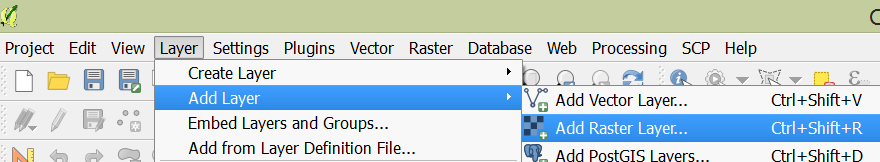


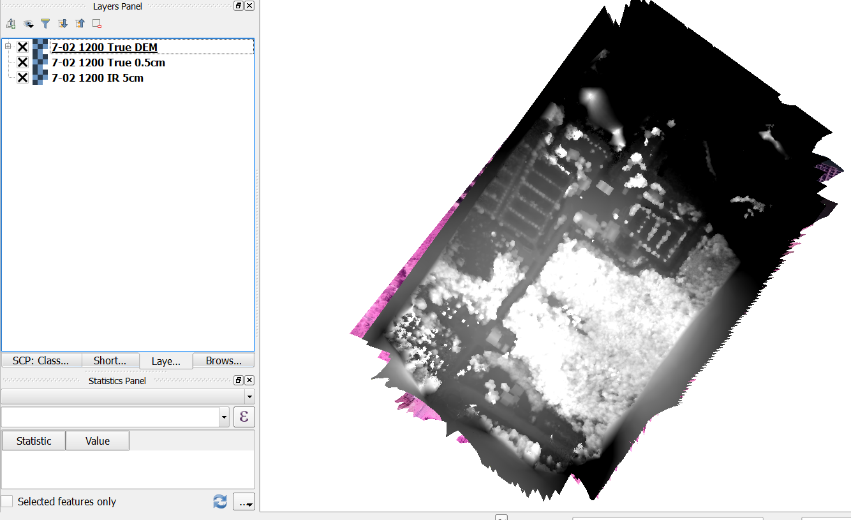


1. To make QGIS works faster, especially when zooming the images, right click on the image list, and choose “Properties”. On the pop up window, choose tab “Pyramids”. Select all list on the “Resolutions” tab, then click “Apply”. Repeat the process for all the images.


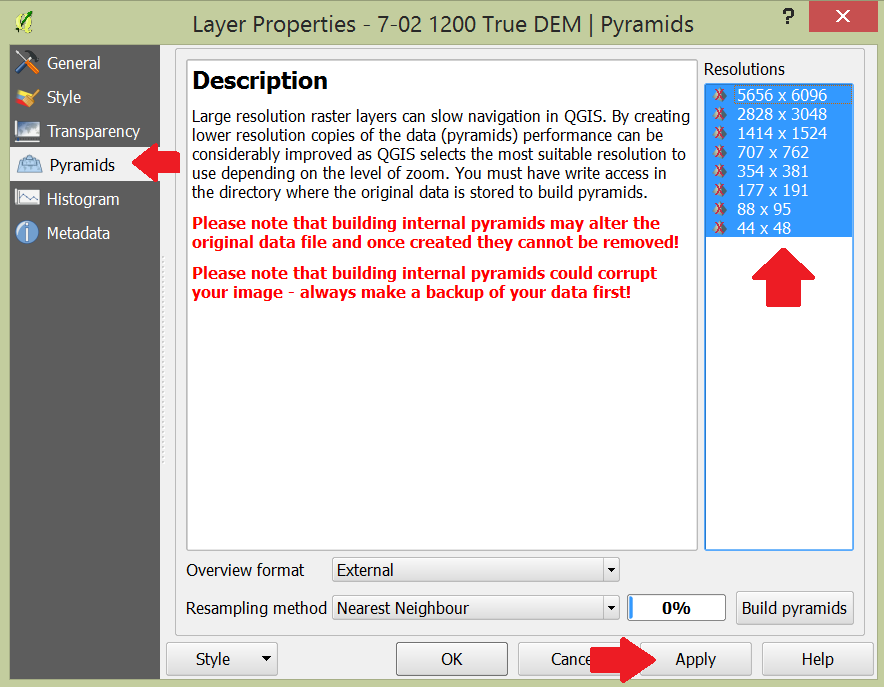


1. Check if the IR and RGB images are perfectly overlapped or not. Click and un-click the tick boxes next to image list on “Layers Panel” to make them appear or disappear. Additionally, open image “Properties” window, click on tab “Transparency” and adjust the “Global transparency”.


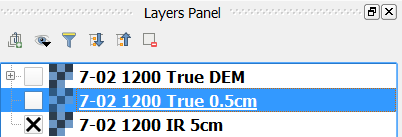

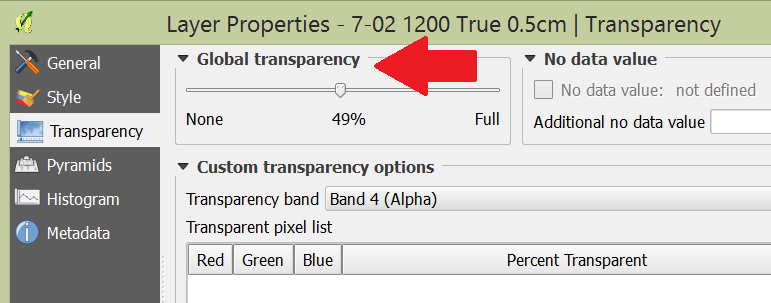


1. (Optional) Reference the IR image with RGB image if they are not perfectly overlapped. On the menu “Raster” choose “Georeferencer”. On the opened Georeferencer window, click menu “File” and then “Open Raster”. Choose the IR image file.


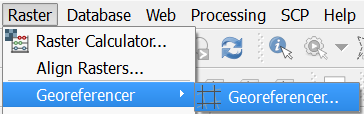

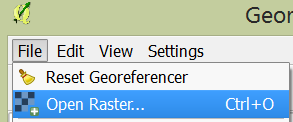


1. (Optional) On the Georeferencer window, click on the landmark which clearly visible on the image as the reference point (corner of a building or small individual tree *etc*.). On the pop up window, click “From map canvas”. Click corresponding point from the RGB image. The coordinates of reference will fill automatically. Repeat the process to obtain at least 3 points (more is better; this research was using 7-8 points).


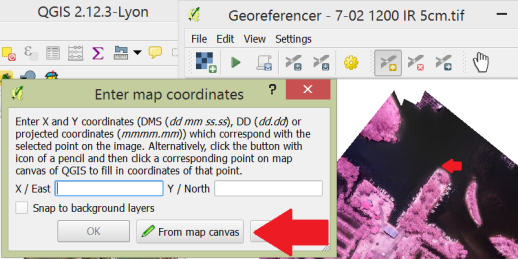

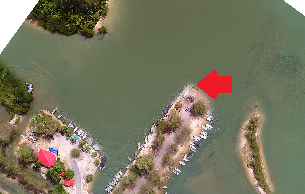


1. (Optional) On the Georeferencer window, click menu “Setting” and “Transformation Setting”. On the Transformation type choose one type (this is trial and error process, so far the Polynomial Transformation gives better result). On the output raster, give the filename. Click OK and wait for the process. Close the Georeferencer window.


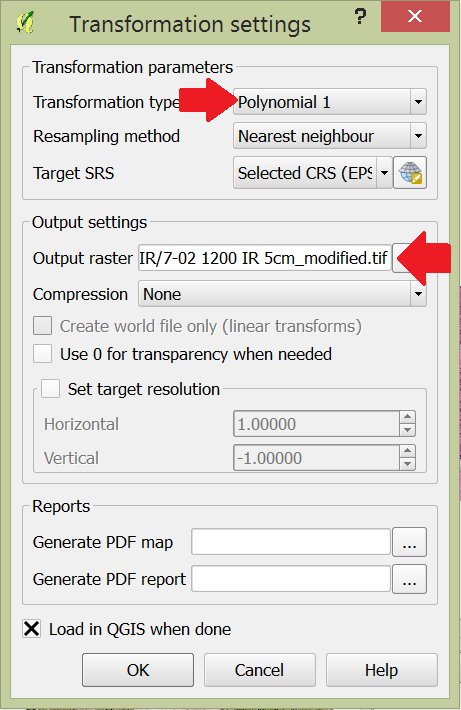


1. If the images are perfectly overlapped, join all the images. On the Processing Toolbox, type “Merge” and double-click on the GDAL Merge algorithm. On the opened Merge window, click on the Input layers and select all the images (IR, RGB and DEM). Tick the “Layer Stack”. On the Merged tab, put the output file name, then, click Run. Wait until the process is finished (approximately 1-2 hours)


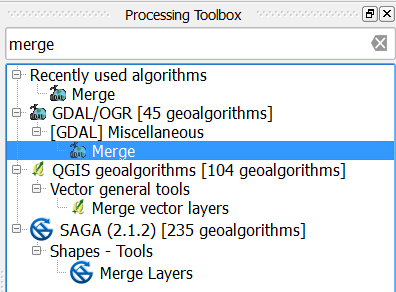

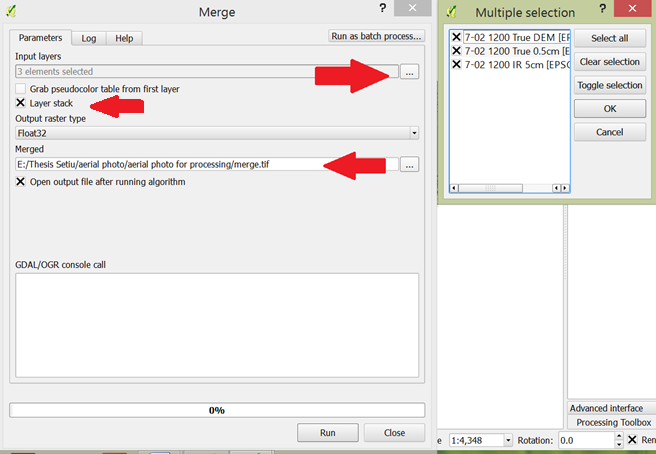


1. Build pyramid for the new merged image (as explained in the step 1)
2. Georeference the image again, but this time use the Satellite Image as the reference coordinate point. Follow the step 6-8 for this process. Build pyramid for the referenced image.

# Paralax and Invalid DEM Cropping

- 1. Open the merged and georeferenced image in the QGIS (by Add Raster Layer or ctrl-shift-R)
  2. Duplicate the image by right click on the image list, then click “Duplicate”
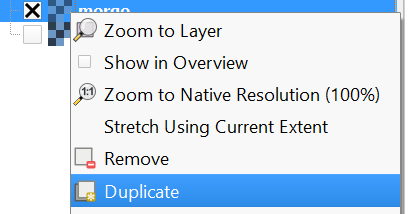

  3. On the merged image, right click and click “Properties”. On the tab “Style”, chose “Multiband color” as Render type. Choose Band 5-6-7 (R, G, B) for true color views. Choose “No Enhancement” for contrast enhancement, and click “Apply”.


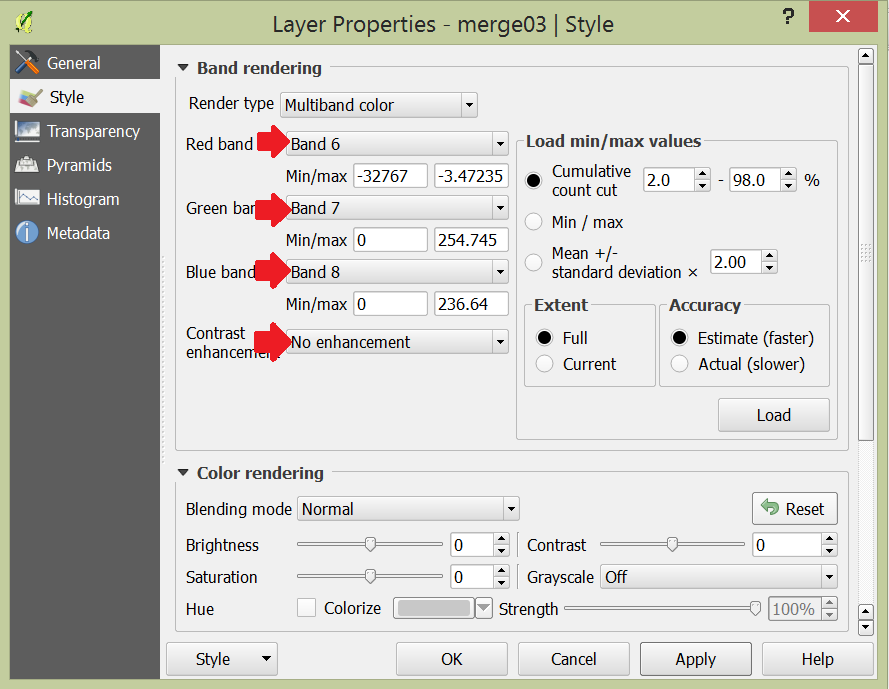


- 1. On the copy of merged image, right click and click “Properties”. In the tab “Style”, chose “Singleband pseudocolor” as Render type. Choose Band 1 (DEM), put value 0-30 (to view DEM of 0 m to 30 m). Click “Classify”, then click “Apply”.


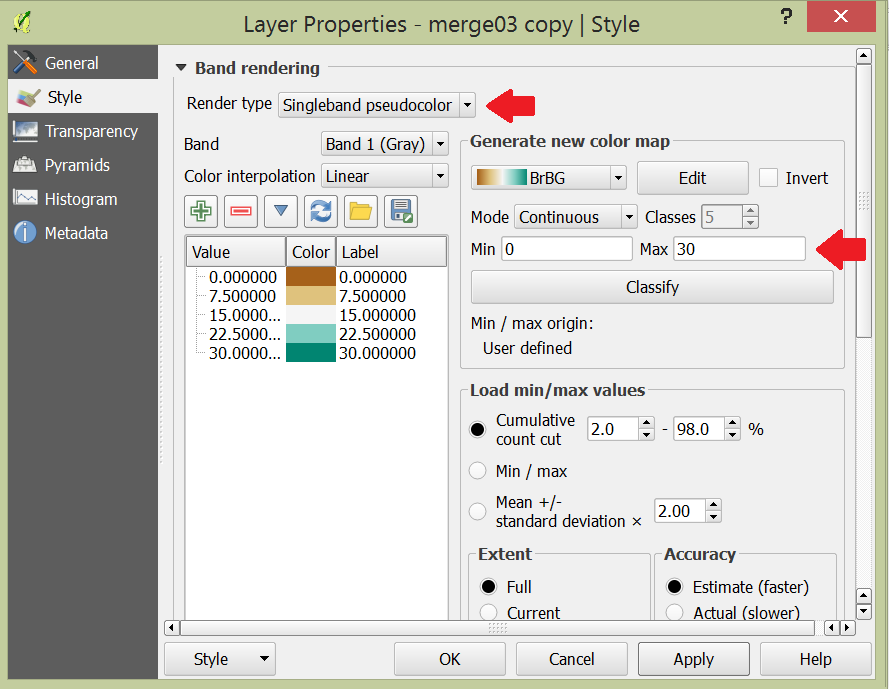


- 1. Compare both image in the format true color/RGB and in the DEM, observe the parallax area (where the trees look elongated outward) and invalid DEM area. This area needs to be cropped.


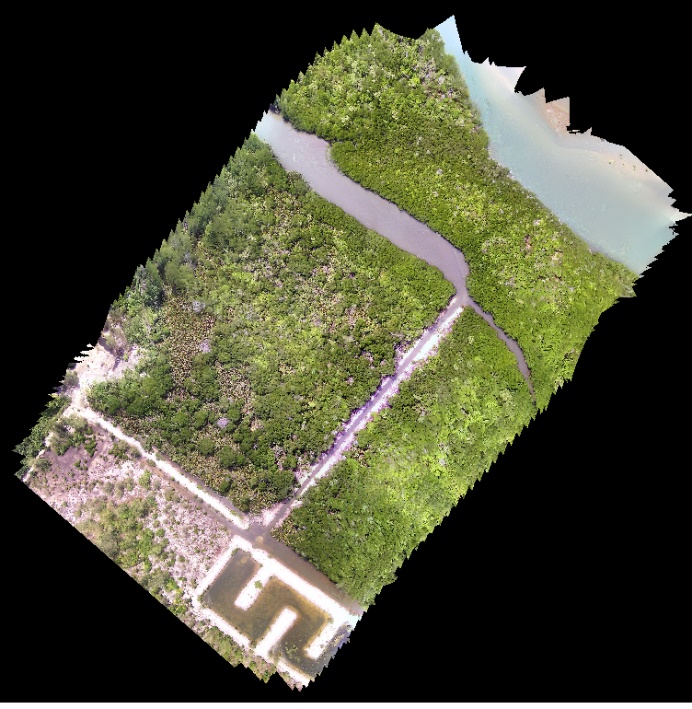

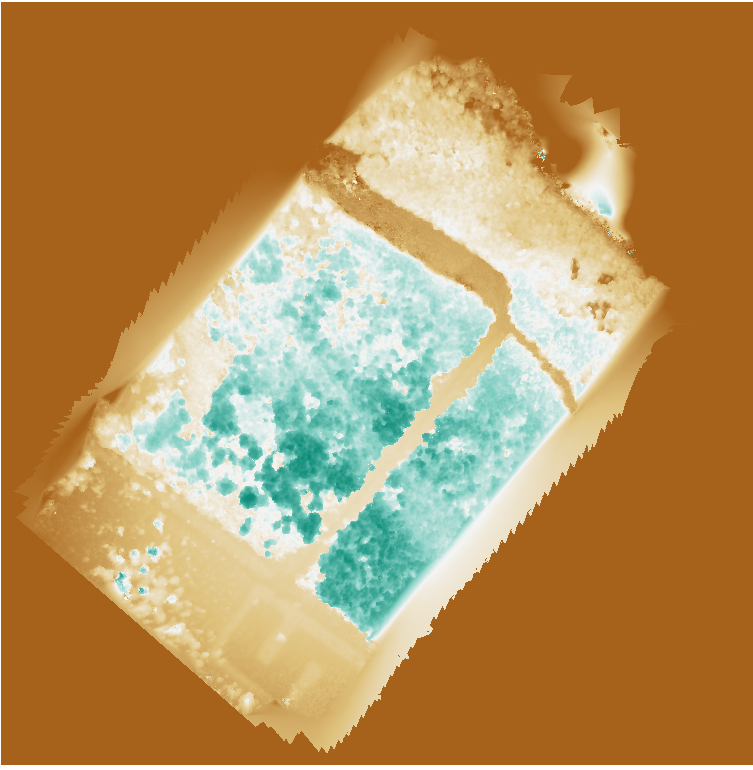


- 1. On the bottom left panel, click icon “New Shapefile Layer”. In the pop-up window, click “polygon” as type, and choose the UTM Zone 48 as coordinate reference system. Click OK and give the file name (“03clip”).
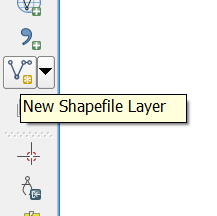

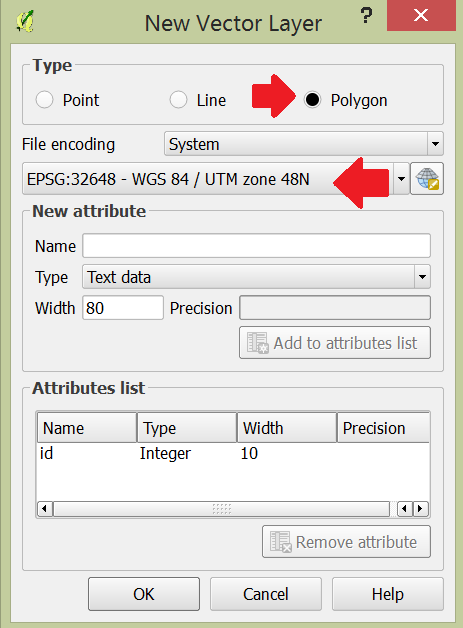

  2. Click the icon “Toggle Editing”
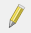
 on Digitizing Toolbar. Then click icon “Add feature”
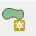
.
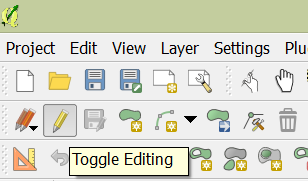

  3. Create a polygon over the image, excluding the parallax and invalid DEM area. After create the polygon, save it by clicking on “toggle editing”
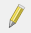
again.


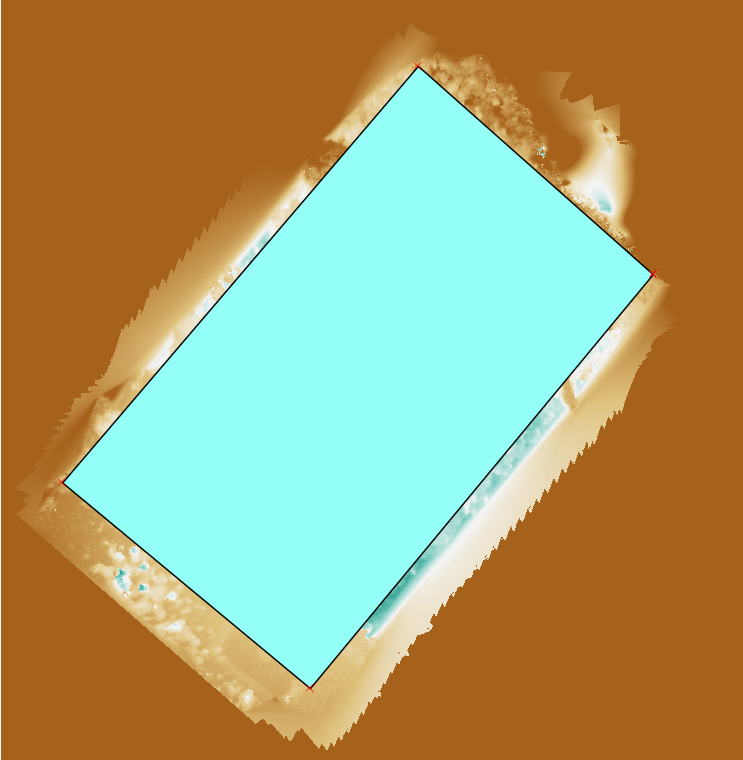


- 1. Click menu “Raster – Extraction – Clipper”. On the pop-up window, put the merged image as input. Give name for the output file. On the clipping mode, choose “Mask Layer” and select the vector polygon layer that just made (“03clip”). Click OK. Wait for approximately 0.5-1 hour until the process is finished. Repeat this process to the Satellite Image, crop it with same vector file (”03clip”).


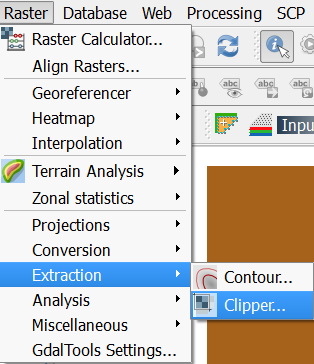

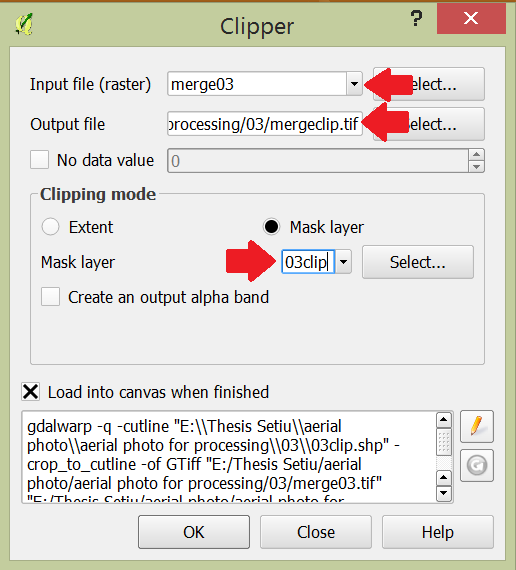


- 1. (Optional, only need to be applied for a large image) To make the clipping process faster, click edit button on the Clipper Window and add this text to the end of script: --config GDAL_CACHEMAX 1000 -wm 1000. This will increase the usage of cache memory to 1000Mb. Increase this cache memory for faster process.


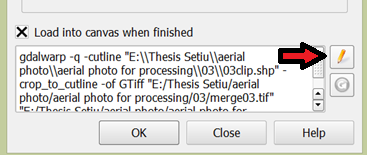


# ROI creation

Complete and original tutorial of SCP Plugin is available in this link

<http://semiautomaticclassificationmanual-v4.readthedocs.io/en/latest/Tutorials.html>

- 1. Click menu “Plugins – Manage and Install Plugin”. Scroll to find Semi-Automatic Classification Plugin and click “Install Plugin”. Menu “SCP” will appear, open it and click “Semi-Automatic Classification Plugin” to activate the plugin


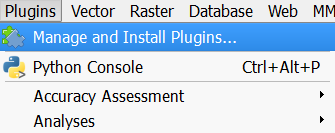

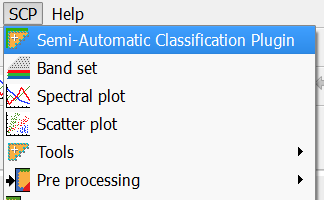

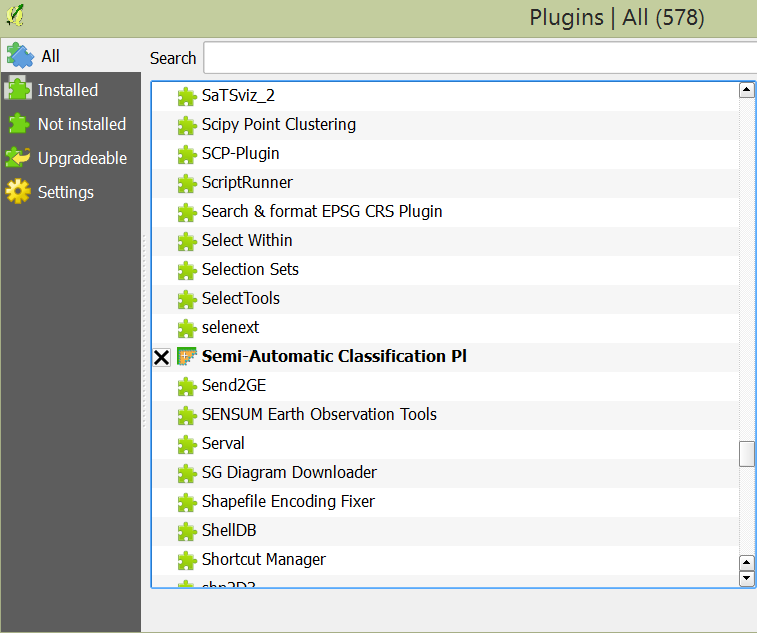


- 1. Open the cropped or clipped raster image from previous section (Open raster layer or Ctrl-Shift-R)
  2. Load the image to plugin. Click the refresh button in SCP toolbar, and chose the raster file name (in this case it is “03clip”)

**
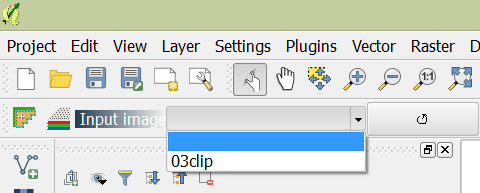
**

- 1. Open the “SCP: ROI creation” Panel (located on the right side of the window). Click “New shp” and give a name to the shapefile (in this case it is “traindroneS”). Choose the class ID (as example “1”) and give the class name (as example “water”). Un-click the “Add. Sig list”


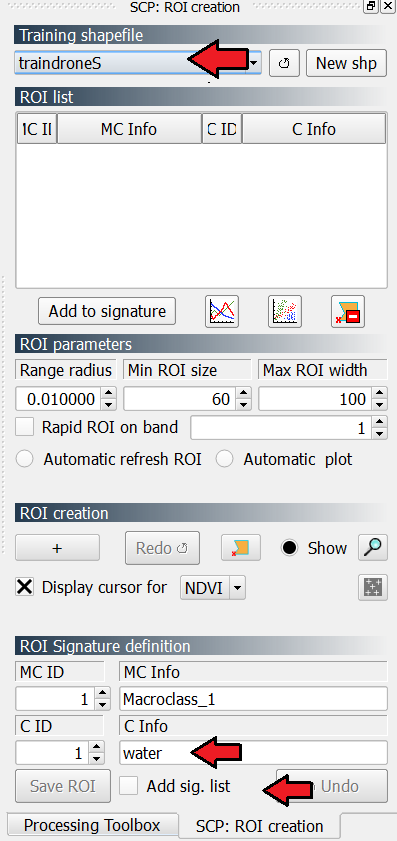


- 1. Click the “Create ROI Polygon”, and draw the polygon over the image. Additionally, you could make sure the polygons have same size by using a measuring tool. Then click “Save ROI”


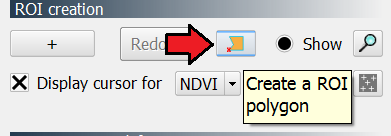

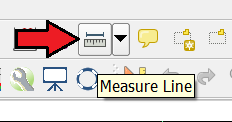

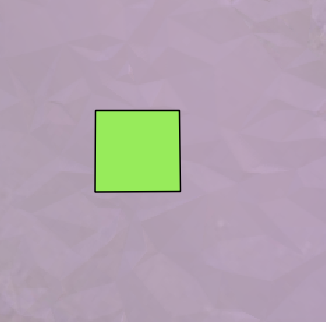


- 1. Repeat the polygon creating process until every class covered by same number of polygons with same coverage area. Use information from field surveys data and photographs. Load the plot data by click menu “Layer – Add Layer – Add delimited Text Layer”. On the opened window, click data contain Adult Tree (“AT plot.csv”) and click OK. The plot data will load into the diplay. Use icon “identify features”
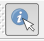
 to click on the plot and to see the information.


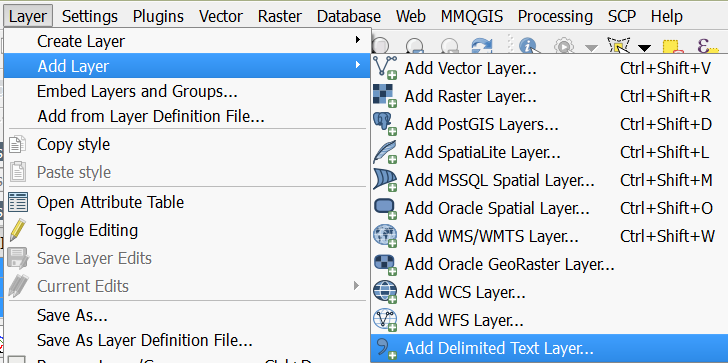

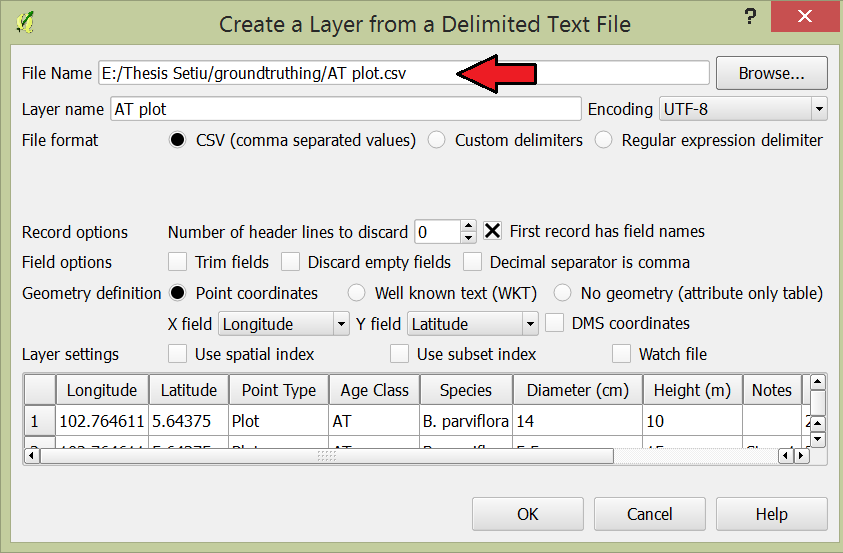

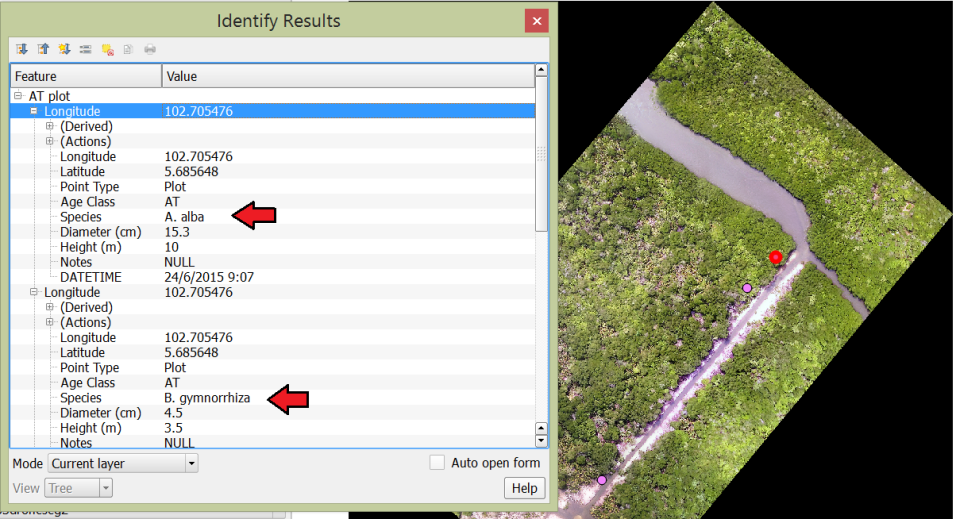


- 1. After ROI polygons are created, select all the ROIs and click “Add to signature”. Wait for 20-30 minutes for drone image and 2-3 minutes for satellite image.


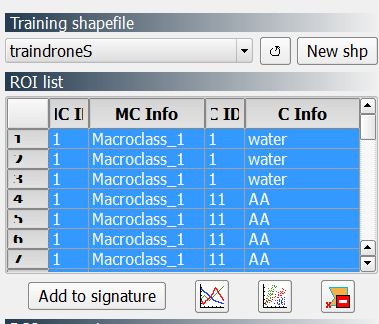


- 1. On the “SCP: Classification” Panel (on the left side) the signatures will appear. Click “Save” and give a name to the signature file (“sigdroneall”). Select all signatures and click icon “Create signature plot”
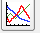
.
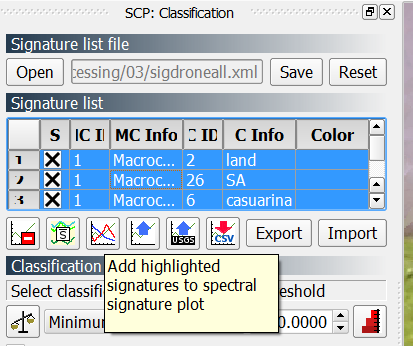

  2. Review the signature, by zooming on the plot. Click on the tab “Signature file” and “Signature distance” to evaluate separation between classes. This overall signature will be used to write manual rule-set. Close the SCP Plot.


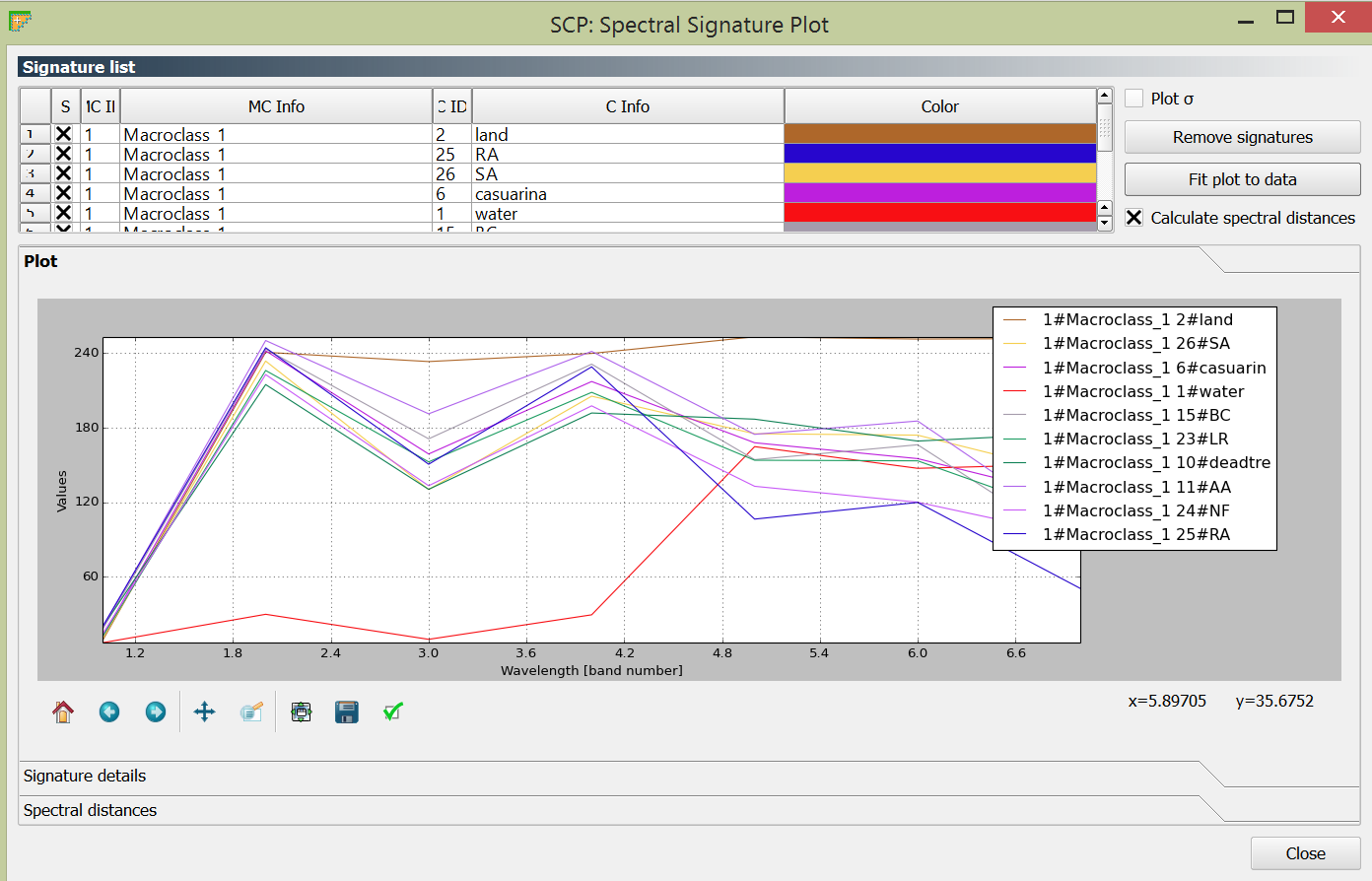


- 1. On the Layers panel, right click on “traindroneS” and click “Save as”. On the opened window, give a name to the file (“traindroneS1A”) and click OK. Repeat the process to 4 more time to create files labeled up to “traindroneS5A”.


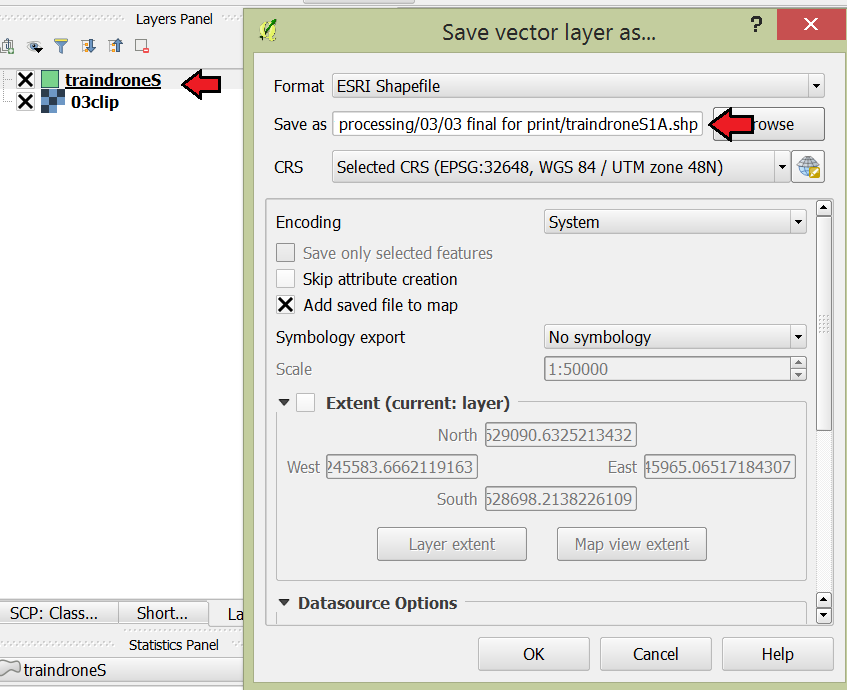

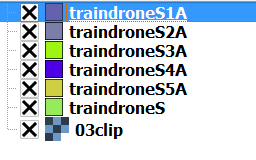


- 1. Click menu “Vector – Research Tools – Random Selection Within Subsets”.
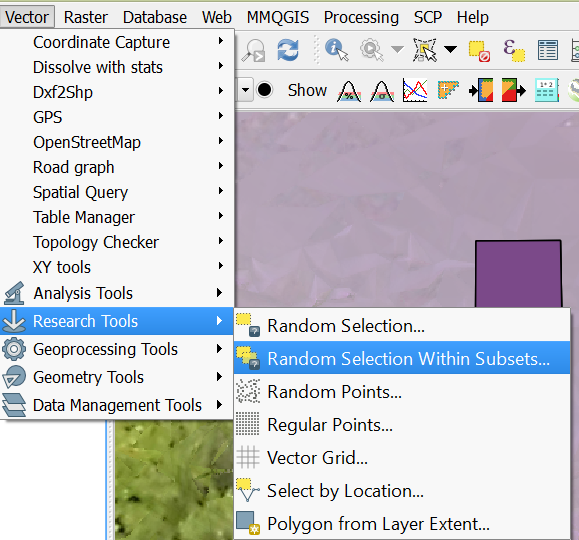

  2. On the opened window, choose “traindroneS1A” as Input Vector Layer. Choose “C_ID” as unique ID field. Choose 50% for percentage of features. Click “OK”. Half of the polygons per class is selected and will be colored yellow on the display.


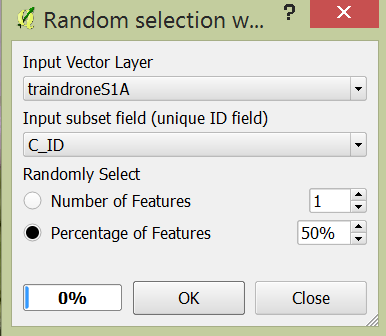

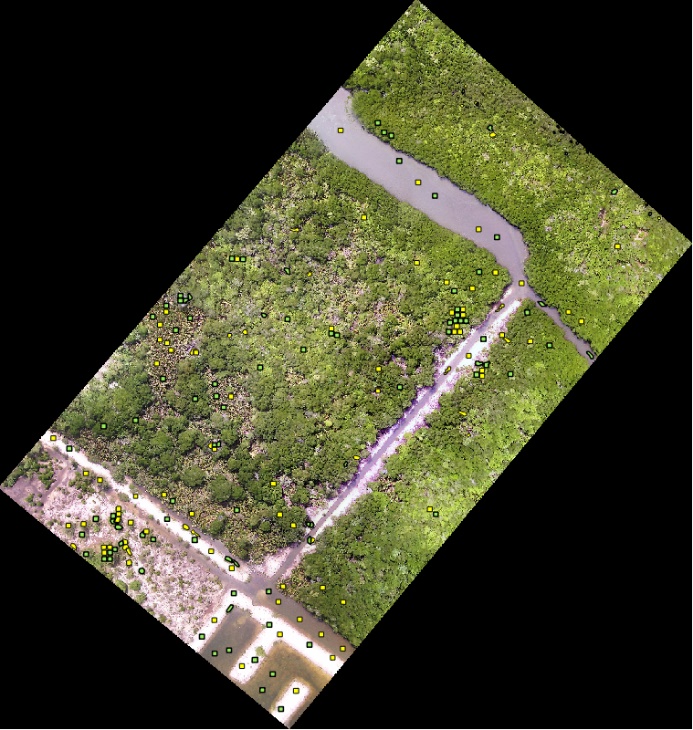


- 1. Right click on “traindroneS1A” in layer panel and click “Save as”. Give the file name “traindroneS1B”. Click “Save only selected features” and click OK. This new shapefile will be loaded into the display.


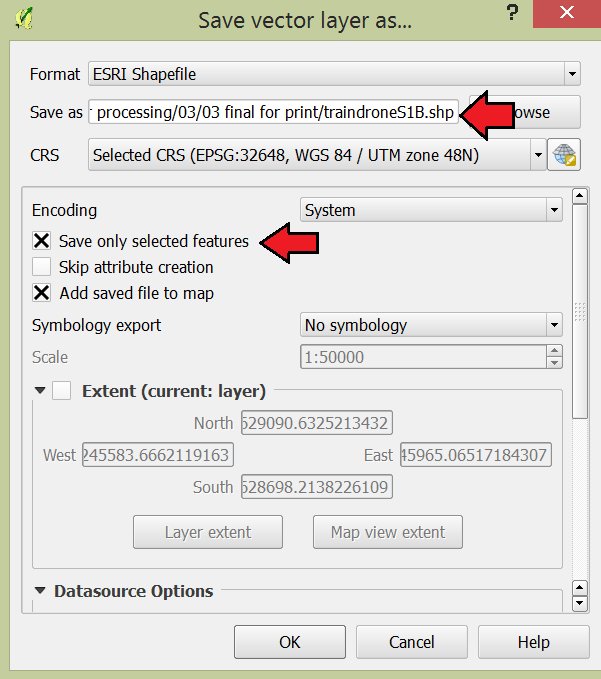


- 1. Click again on “traindrone1A” and click icon “toggle editing”
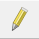
 on the digitizing toolbar. Click icon “delete”
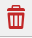
 to delete the selected polygons. Then click again on the icon “toggle editing”
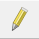
 to save the change. Now a pair of training sites is finished. Repeat the process to “traindrone2A” to create “trainedrone2B” and so on.

# Segmentation

This part is based on this tutorial

<http://wiki.awf.forst.uni-goettingen.de/wiki/index.php/Object-based_classification_(Tutorial)>

- 1. Open clipped/cropped raster image in QGIS (Add raster layer/ctrl-shift-R).
  2. On the Processing Toolbox panel, type “segmentation”. Click on the “Exact Large-Scale Mean-Shift segmentation, step 1”.


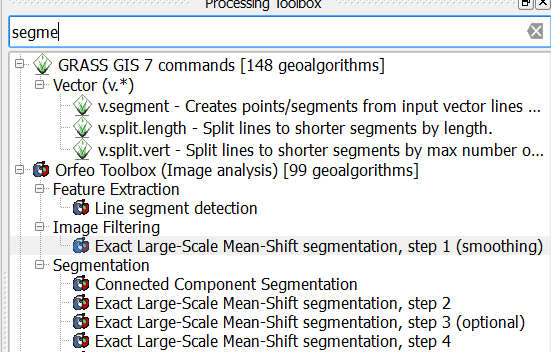


- 1. On the opened window, put the raster file name (“03clip”) and put the Available RAM (Mb) based on the computer’s capacity. The standard was 128 Mb, but higher RAM allocation will make the process runs faster. Click “Run”. Wait 3-4 hours until the process is finished, then a result called “Filtered output” will appears on the Layers panel.


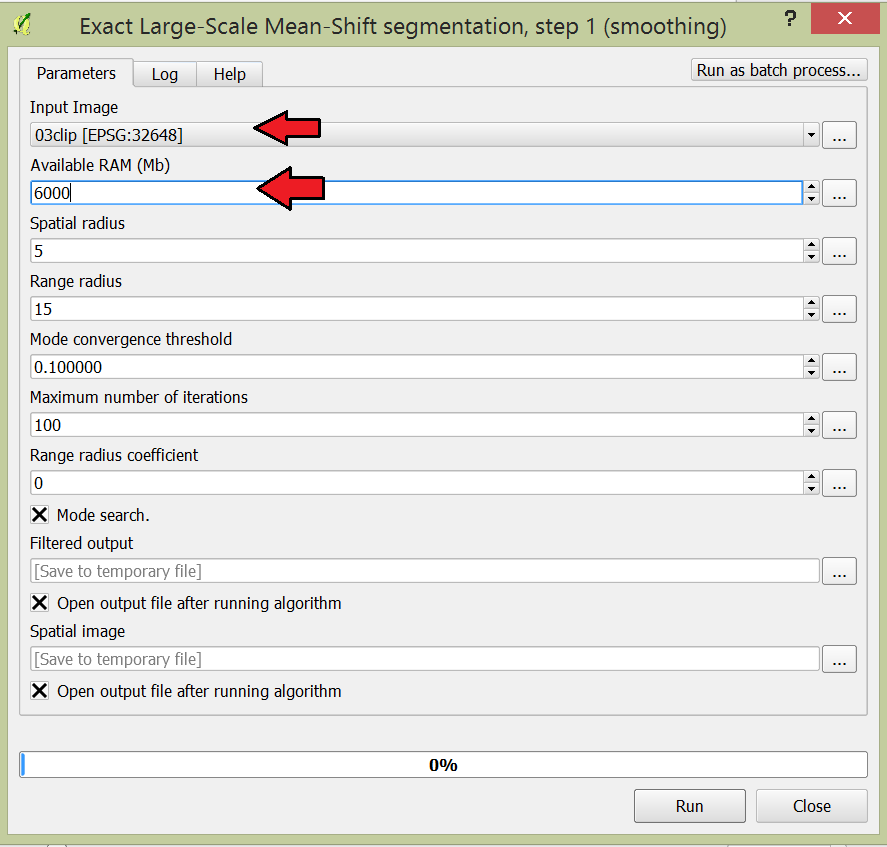


- 1. Click “Exact Large-Scale Mean-Shift segmentation, step 2”. Put the “Filtered Output” as filtered image. Then click Run. Wait around 1 hour until the process is finished, then a result called “Output image” will appear on the Layers panel.


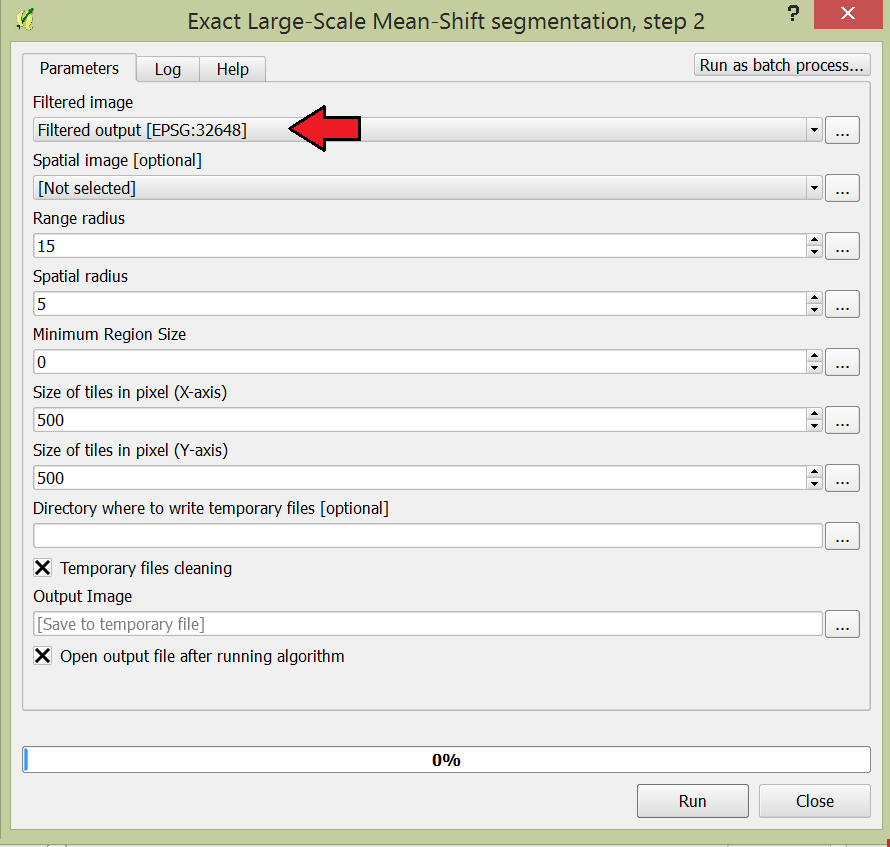


- 1. Click “Exact Large-Scale Mean-Shift segmentation, step3”. Put the “03clip” as Input image and “Output image” as segmented image. Then click “Run”. Wait around 1 hour until the process is finished, then a result called “Output image” will appears on the Layers Panel. As there is 2 “Output image”, delete the previous one from step 2.
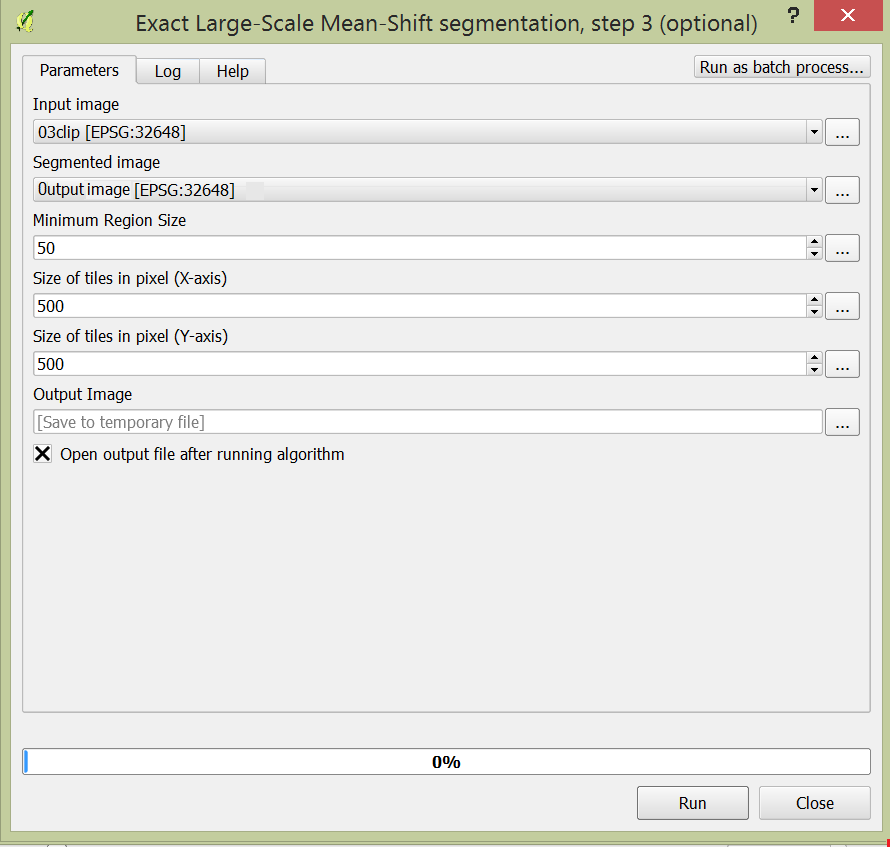

  2. Click “Exact Large-Scale Mean-Shift segmentation, step3”. Put the “03clip” as Input image and “Output image” as segmented image. Give the file name for segmentation result (“03droneseg”). Then click “Run”. Wait around 2 hours until the process is finished. Open the resulting segmentation file (Open vector layer/ctrl –shift-V).
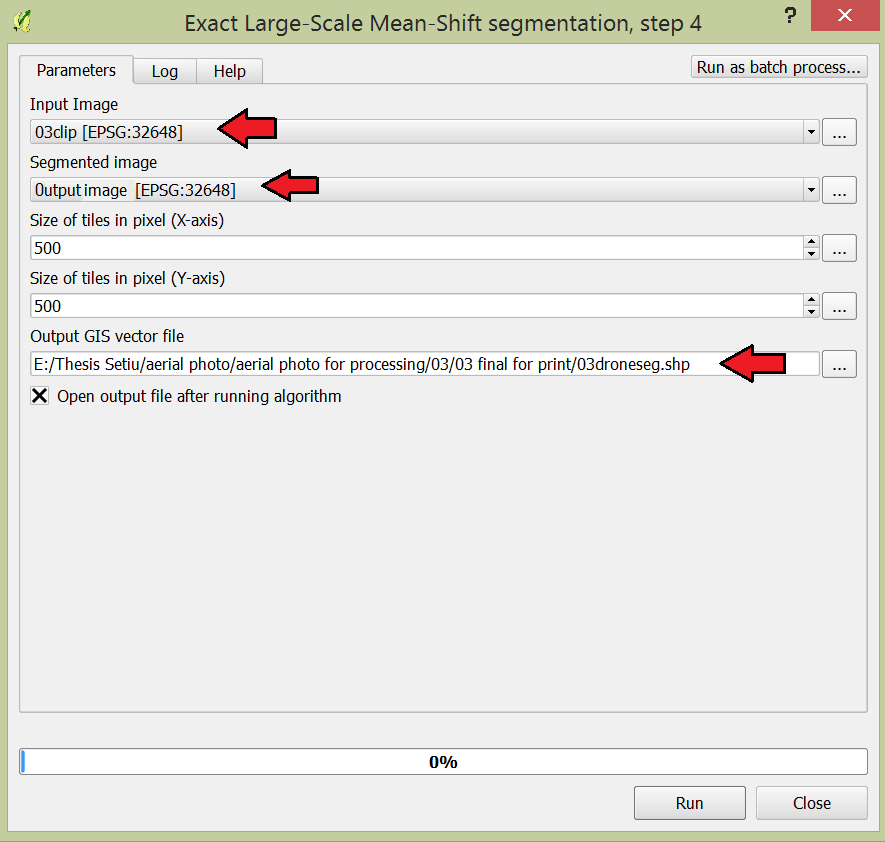

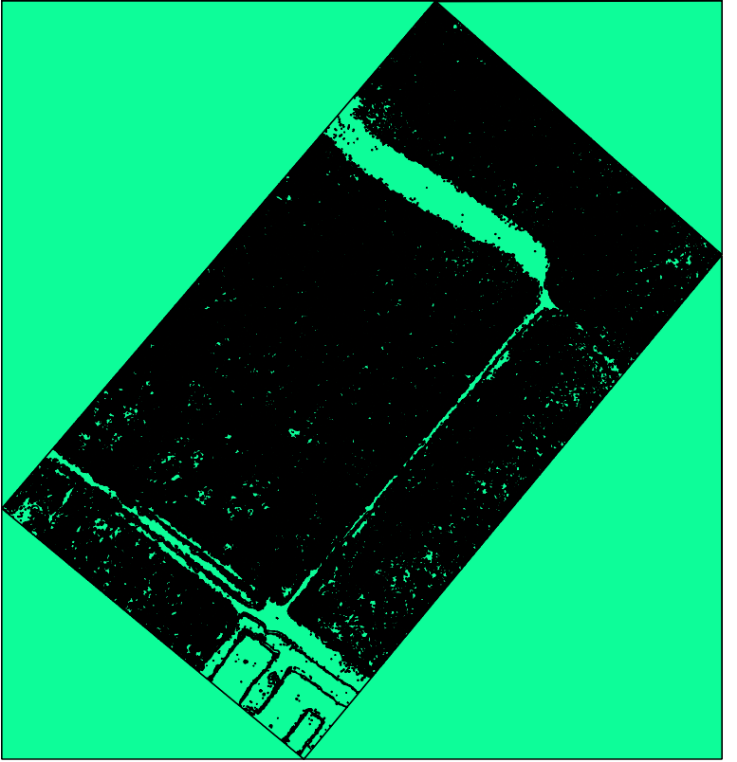

  3. Repeat the segmentation process on the satellite image.

# Object-based Manual Classification

- 1. Load the signature file “sigdroneall” on the “SCP: Classification” Panel and observe the signature plot. Use also the icon “identify features”
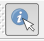
 and click on the segment to inquire the information of that particular certain segment.
  2. Open “droneseg03” properties. Click the tab Style and choose “Rule Based”. Start to write the rule from easiest feature to classify (e.g. water and land), until all designed features are covered. Increase the layer transparency. Click Apply to see the result. Add, refine and change the rule based on your visual judgment until a good classification is achieved.


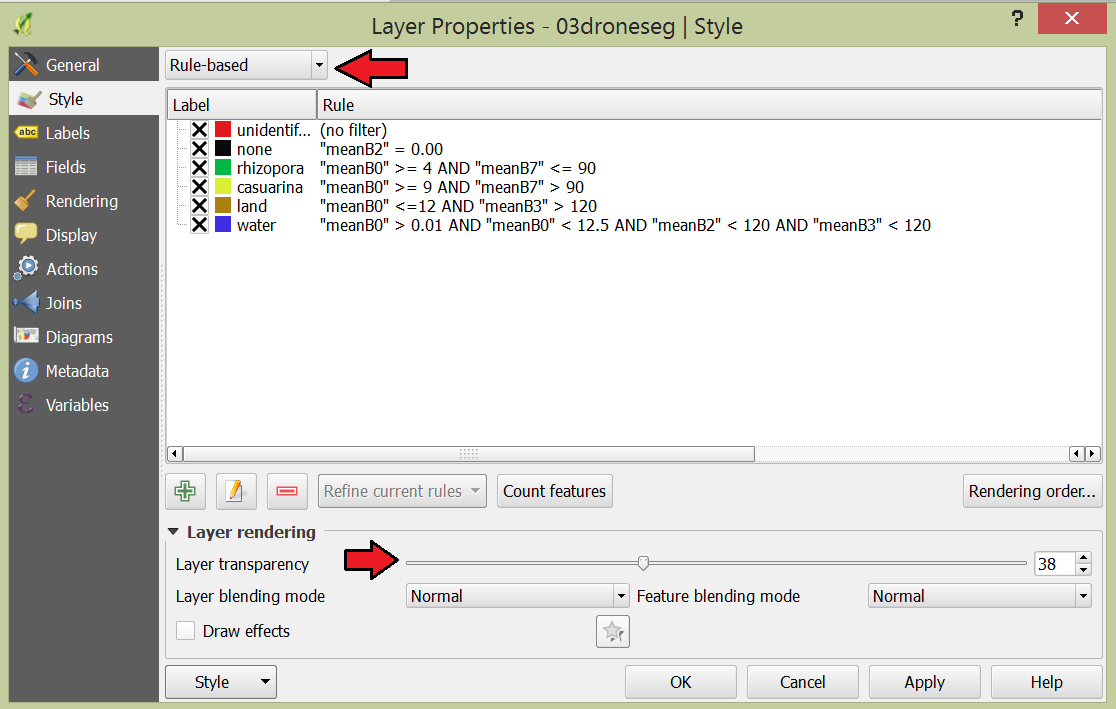

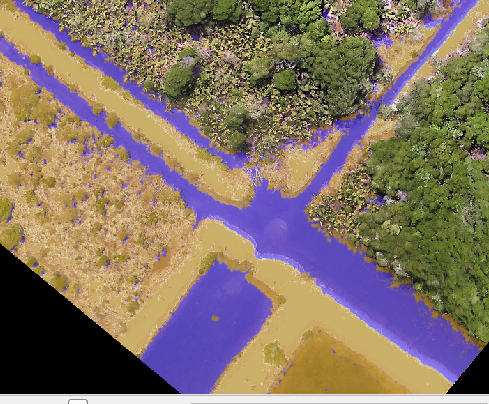


- 1. fter the rule is finished, click icon “Open Attribute Table”
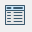
 to open attribute table of the segments. On the opened window, click icon “Open Field Calculator”
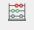
to open the Field Calculator


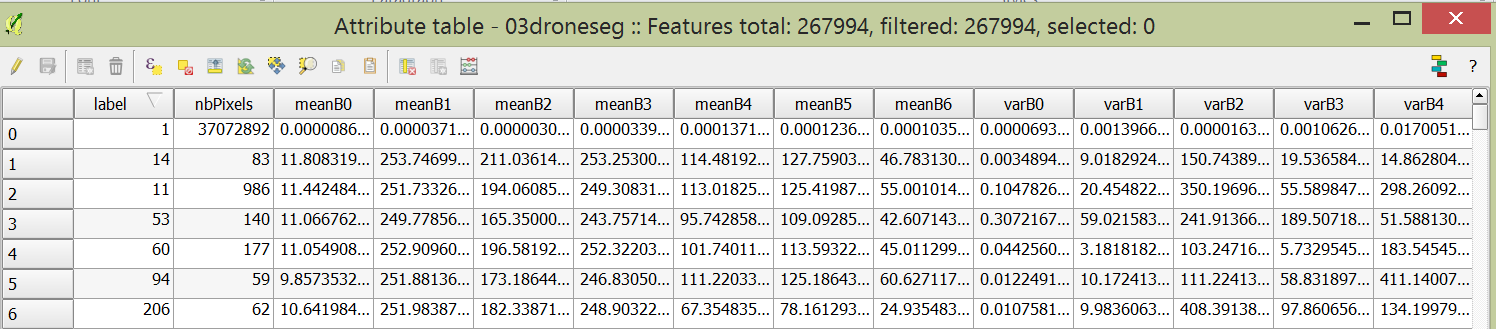


- 1. Put “classS” as Output field name. Choose “Text/String “as Output field type, and rewrite the rules from the previous step. As example:

CASE

WHEN "meanB1" <120 AND "meanB0" <12 THEN 'water'

WHEN "meanB1" > 120 AND "meanB0" < 12 THEN 'land'

WHEN "meanB0" >=12 AND "meanB0" <=18 THEN 'NF'

WHEN "meanB4" <160 AND "meanB0" > 18 THEN 'RA'

WHEN "meanB4" >=160 AND "meanB2" <160 AND "meanB0" >18 THEN 'deadtree'

WHEN "meanB4" >=160 AND "meanB0" > 18 THEN 'AA'

ELSE 'RA'

END

- 1. Click OK. Wait until the process is finished. A new column “classS” will appear. Click icon to save the change. Now the segment is classified based on the rule.
  2. Give the class integer code, using field calculator. This step is necessary because accuracy analysis only accept integer class label. Click icon to save the change. Now the segment is having integer label.

# Object-based Automatic Classification

We use satellite image in batch process as example here

- 1. Open all of the paired training files (trainsat1A-1B to trainsat5A-5B). Open also the segmentation result of satellite image (“03satseg”). On the “Processing Toolbox”, choose “Join attributes by location”

- 1. On the opened window, click “Run as batch process”. Click icon “add row / +” to add more rows. Open 10 rows in total.

- 1. Add “03satseg” as Target vector layer. Add paired training files (trainsat1A-1B to trainsat5A-5B) as Join vector layer.

- 1. Scroll right, tick “contains”, “equal”, “touches”, “overlaps”, and “within”.
  2. Scroll more to the right. In joined layer, give the file name “orgsat1A” to “orgsat5B”. Then click “Run”.
  3. Remove the joined layer. Open the vector file “orgsat1A” to “orgsat5B” and click icon Table Manager. If you do not have table manager, install this plugin.

- 1. On the opened window, delete the fields “MC_ID”, “MC_info” and “C_info”. Change the “classID” to “class”. Click “Save”.

- 1. Minimize the QGIS. Open software called OSGeo4WShell, which is installed together with QGIS.

- 1. Type otbgui_ComputeOGRLayersFeaturesStatistics and press enter.
  2. On the opened window, put the “03satseg.shp” as input shapefile, and create “03satseg.xml” as XML file containing mean and variance of each feature. Click “meanB0” to “meanB6” as features to consider. Click “Execute”.

- 1. Put the “orgsat1A.shp” as input shapefile, and create “orgsat1A.xml” as XML file containing mean and variance of each feature. Click “meanB0” to “meanB6” as feature to consider. Click Execute. Repeat this process to “orgsat1B” and so on. Close the ComputeOGR window.

- 1. Type otbgui_TrainOGRLayersClassifier and press enter.
  2. Put the “orgsat1A.shp” as input shapefile, and put “orgsat1A.xml” as XML file containing mean and variance of each feature. Create Output model filename and name it “orgsat1A.model”. Click “meanB0” to “meanB6” as features to consider. Click “Execute”. Repeat this process to orgsat1B and so on. Close the TrainOGR window.
  3. Type otbgui_OGRLayeClassifier and press enter.
  4. Put the “03satseg” as input shapefile, and put “03satseg.xml” as XML file containing mean and variance of each feature. Put “orgsat1A.model” as input model filename. Click “meanB0” to “meanB6” as features to consider. Type “class1A” on the “Field containing predicted class”. Click “Execute”. Repeat this process, using “orgsat1B.model” to create “class1B” and so on. Close the window and close OSGeo4WShell
  5. On QGIS, close and load 03satseg.shp. Click icon to open attribute file. Now the shapefile is containing class1A – class5B, which contain classification for each segments.

# Merging Classified Vector and Convert to Raster

In this example we merge the class in batch process.

1. On the “Processing Toolbox” panel, open Polygon dissolve (by attribute), then click “Batch Process”.
2. Add more rows (we need 10 rows). Put “03satseg” as Polygons, and put “class1A
    to “class5B” as attribute. Choose “No” for Keep inner boundaries.
3. Give the name of dissolved file “SOA1A” to “SOA5B”– for Satellite Object-based Automatic classification. Click Run.

1. Open the vector files “SOA1A” to “SOA5B”. On the “Processing Toolbox” panel, click “Rasterize (vector to raster)”
2. Click Batch Process. Put “SOA1A” to “SOA5B” as input layer, and “class1A” to “class 5B” as attribute field.
3. Scroll to the right. Put 1000 for Horizontal and Vertical value (for drone put 3000).
4. Scroll to the right. Give name to the raster .tif file “SOA1A” to “SOA5B”. Click “Run”
5. Also dissolve and convert (without using the batch process) the manual-rule result using same step.

# Pixel-based Maximum Likelihood and Spectral Angle Mapping Classification

This example is using drone image with 10 classes

- 1. Open the clipped drone image and ROI sets “traindroneS1A” to “traindroneS5B”

- 1. On “SCP: ROI creation” panel, Choose “traindroneS1A” as the training shapefile. Click “Add to signature”. Wait around 30 minutes to 1 hour.

- 1. On the “SCP: Classification” Panel, save the signature file and give a name (“sigdrone1A.xml”). On the “Select Classification algorithm”, choose “Maximum Likelihood”. Then click on the “Perform Classification”. Give the file name “DSPML1A”. Wait until the process is finished (around 45 minutes to 1 hour).

- 1. Repeat step 3, but change the classification algorithm to Spectral Angle Mapping and save the filename as “DSPSAM1A”.
  2. Repeat from step 2-4 for different ROI, from “traindroneS1B” to “traindroneS5B”

# Accuracy Analysis

In this example, we are assessing the Drone Manual-rule results.

- 1. Click on the icon “Post Processing” in SCP Toolbar.
  2. Choose classification result file (in this case named “DSOM”) as classification to assess, and the training site “traindroneS1A” as reference. Choose “C_ID” on the Shapefile field. Click Calculate Error Matrix. Give the filename to results “DSOM1A”
  3. This will resulting in an error matrix. Result also availablein a .csv document, located in the same directory. This “accDSOM1A.csv” can be opened in Microsoft Excel.

- 1. Repeat the process for all ROIs and all classified image. For Manual rule, test them with each ROI. For other classification (Automatic/OGR, ML and SAM), test them with complimentary training file (1A tested by 1B, 3B tested by 3A and so on)

# Pontius Matrix

1. Download the Pontius Matrix Excel file from <http://www2.clarku.edu/~rpontius/PontiusMatrix41.xlsx>
2. On the tab Sample Count, rename the category into the classes names (water, land etc)
3. Copy and paste the error matrix into Pontius.

1. For sample population, open QGIS and open the classified map (in this case “DSOM”). On the “Processing Toolbox”, chose “r.report” algorithm.

1. Select “DSOM” as raster layer to report on. Select “c” or cell count as unit. Click “Run”.
2. Copy the cell count (leave the value 0 as it is unclassified) then paste it into pontius matrix
3. On the tab “Figures Data”, Quantity, Exchange and Shift values can be obtained.
4. On the tab “Category Component”, we could see which class is over or underestimate. Here the dead tree quantity is overestimated and land quantity is underestimated.
