# Supplementary material for: The advantages of using drones over space-borne imagery in the mapping of mangrove forests
Source: PLoS One. 2018 Jul 18;13(7):e0200288. doi: 10.1371/journal.pone.0200288 (PMC6051606; doi:10.1371/journal.pone.0200288)
Supplement: S3 File — (PDF) [file pone.0200288.s003.pdf]

UNIVERSITI MALAYSIA TERENGGANU
21030 Kuala Terengganu,
Terengganu, Malaysia
Web : www.umt.edu.my
INSTITUT OSEANOGRAFI DAN SEKITARAN
Tel : 09-6683195 | Faks : 09-6692166
Higher Institution Centre of Excellence (HICoE) in Marine Science

Our Reference : UMT/INOS/2-2/0
Date : 18 March 2018

To whom ever it may concern,

AUTHORIZATION OF THE PLEIADES-1B SATELLITE DATA DATED 16 AUGUST 2013 (SPATIAL RESOLUTION: 2 M)

This is to authorise that the Pleiades-1B Satellite data dated 16 August 2013 (spatial resolution: 2 m) used for mapping the mangroves at Setiu wetland (in the manuscript entitled, "Like a drone come true: the advantages of using drone over space-borne imagery in the mapping of mangrove forests" submitted by Ruwaimana_etal_2017 to PLoS ONE PONE-D-17-43261) was procured by the Institute of Oceanography and Environment (INOS), Universiti Malaysia Terengganu (UMT), as part of the coastal resources monitoring and management in Peninsular Malaysia.

Therefore, if there are any inquiries further may kindly be forwarded to the Director of the INOS, UMT.

Thank you.

"BERKHIDMAT UNTUK NEGARA"
"Soaring Upwards"

Yours sincerely,

PROF. MADYA DR. AIDY @ MOHAMED SHAWAL BIN M MUSLIM
Director
Institute of Oceanography and Environment
Universiti Malaysia Terengganu
☎ 09-6683101/3102
✉ aidy@umt.edu.my

c.c 1) File
