# Supplementary material for: The advantages of using drones over space-borne imagery in the mapping of mangrove forests
Source: PLoS One. 2018 Jul 18;13(7):e0200288. doi: 10.1371/journal.pone.0200288 (PMC6051606; doi:10.1371/journal.pone.0200288)
Supplement: S4 File — (PDF) [file pone.0200288.s004.pdf]

UNIVERSITI MALAYSIA TERENGGANU
UMT
21030 Kuala Terengganu, Terengganu, Malaysia
Web : www.umt.edu.my
INSTITUT OSEANOGRAFI DAN SEKITARAN
Tel : 09-6683195 | Faks : 09-6692166
Higher Institution Centre of Excellence (HICoE) in Marine Science

Our Reference : UMT/INOS/P/2-2/0
Date : 10 June 2018

Dear Ms. MONIKA RUWAIMANA,

REF: PUBLICATION IN PLOS ONE - ***THE ADVANTAGES OF USING DRONES OVER SPACE-BORNE IMAGERY IN THE MAPPING OF MANGROVE FORESTS***

I refer to your application for Ethical Biosecurity Committee regarding the above publication purpose - to support your data collected from Setiu wetlands (Terengganu) be publishable, I am pleased to inform you the authorities at the Institute of Oceanography and Environment (INOS), UMT, have approved the request.

Please be aware that the approval is conditional on following –

1. Ethical Biosecurity Committee will require a copy of the publication, upon its acceptance in the PLoS ONE

If you have any queries, please don't hesitate to contact the INOS authorities.

Thank you.

"BERKHIDMAT UNTUK NEGARA"

Your faithfully,

ASSOCIATE PROF. DR. AIDY @ MOHAMED SHAWAL M MUSLIM
Director
Institute of Oceanography and Environment
Universiti Malaysia Terengganu
☎ +06 09-668 3101
✉ aidy@umt.edu.my

c.c 1) File

Ethical Biosecurity Committee
Institute of Oceanography and Environment
Universiti Malaysia Terengganu
T145, 21030 Kuala Terengganu, Terengganu, Malaysia
Ph. +60 9 668 4100, Fax: +60 9 669 2166

Associate Prof. Dr. Aidy Mohamed Shawal B. Muslin	Chairman
Associate Prof. Dr. Suhaimi B. Suratman	Member
Associate Prof. Dr. Mohd Fadzil B. M. Akhir	Member
Associate Prof. Dr. Zianudin Bachok	Member
Associate Prof. Dr. Behara Satyanarayana	Member
Dr. Le Quang Dung	Member
